# NHR-23 activity is necessary for *C. elegans* developmental progression and apical extracellular matrix structure and function

**DOI:** 10.1242/dev.201085

**Published:** 2023-05-22

**Authors:** Londen C. Johnson, An A. Vo, John C. Clancy, Krista M. Myles, Murugesan Pooranachithra, Joseph Aguilera, Max T. Levenson, Chloe Wohlenberg, Andreas Rechtsteiner, James Matthew Ragle, Andrew D. Chisholm, Jordan D. Ward

**Affiliations:** ^1^ Department of Molecular, Cell, and Developmental Biology, University of California Santa Cruz, Santa Cruz, CA 95064, USA; ^2^ Department of Cell and Developmental Biology, School of Biological Sciences, University of California San Diego, La Jolla, CA 92093, USA

**Keywords:** *C. elegans*, Molting, NHR-23, Nuclear hormone receptor, Apical extracellular matrix, Auxin-inducible degron

## Abstract

Nematode molting is a remarkable process where animals must repeatedly build a new apical extracellular matrix (aECM) beneath a previously built aECM that is subsequently shed. The nuclear hormone receptor NHR-23 (also known as NR1F1) is an important regulator of *C. elegans* molting. NHR-23 expression oscillates in the epidermal epithelium, and soma-specific NHR-23 depletion causes severe developmental delay and death. Tissue-specific RNAi suggests that *nhr-23* acts primarily in seam and hypodermal cells. NHR-23 coordinates the expression of factors involved in molting, lipid transport/metabolism and remodeling of the aECM. NHR-23 depletion causes dampened expression of a *nas-37* promoter reporter and a loss of reporter oscillation. The cuticle collagen ROL-6 and zona pellucida protein NOAH-1 display aberrant annular localization and severe disorganization over the seam cells after NHR-23 depletion, while the expression of the adult-specific cuticle collagen BLI-1 is diminished and frequently found in patches. Consistent with these localization defects, the cuticle barrier is severely compromised when NHR-23 is depleted. Together, this work provides insight into how NHR-23 acts in the seam and hypodermal cells to coordinate aECM regeneration during development.

## Teaser

**Summary:** Activity of NHR-23, a *C. elegans* transcription factor, is necessary in seam and hypodermal cells to promote molting and timely development.

## INTRODUCTION

Molting is a crucial developmental process required for the growth of all ecdysozoans: a clade comprising an estimated total of 4.5 million living species (Telford et al., 2008). Invertebrate molting involves conserved processes such as apical extracellular matrix (aECM) remodeling, intracellular trafficking and oscillatory gene expression (Lažetić and Fay, 2017). Molting is also a process of interest for developing new drugs against parasitic nematodes (Ghedin et al., 2007). Parasitic nematodes cause over a billion human infections each year and loss of livestock and crops (Ward, 2015b). However, very few drugs exist, and resistance to those drugs is emerging rapidly (Ward, 2015b). Although many genes involved in nematode molting have been identified in *C. elegans* (Frand et al., 2005)*,* little is known about how their gene products are coordinated to promote aECM remodeling and the generation and release of a new cuticle.

Nematodes progress through four periodic larval stages (L1-L4) before becoming a reproductive adult (Brenner, 1974). The end of each larval stage is punctuated by a molt that involves trafficking and secretion of aECM components, assembly of a new aECM underneath the old cuticle, followed by separation of the cuticle from the underlying aECM (apolysis) and shedding of the old cuticle (ecdysis) (Lažetić and Fay, 2017). Apolysis coincides with a sleep-like behavior called lethargus (Singh and Sulston, 1978). The cuticle is a collagenous exoskeleton secreted by hypodermal and seam epithelial cells (Page and Johnstone, 2007). The outermost layer (glycocalyx) is rich in carbohydrates and mucins (Nelson et al., 1983; Singh and Sulston, 1978). Beneath the glycocalyx is the glycolipid- and lipid-rich epicuticle, which is postulated to function as a hydrophobic surface barrier (Blaxter, 1993; Blaxter et al., 1992). Underlying the epicuticle is a layer comprising mainly cuticlin proteins. Between the epidermal membrane and the epicuticle are collagen-rich layers, including a fluid-filled medial layer with collagen struts (Lažetić and Fay, 2017; Page and Johnstone, 2007). Sets of collagens oscillate in expression over the course of each molt and are classified into three groups based on temporal expression: early, intermediate and late collagens (Johnstone and Barry, 1996; Page and Johnstone, 2007). Although the specific localization of most collagens is unknown, collagens from the same group are thought to be in the same layer (Lažetić and Fay, 2017). A transient structure, the sheath or pre-cuticle, is formed during each larval stage and is thought to pattern the cuticle (Cohen and Sundaram, 2020). Many of the components of this pre-cuticle are related to mammalian matrix proteins. The *C. elegans* pre-cuticle contains zona pellucida proteins, proteins related to small leucine-rich proteoglycans and lipid transporters in the lipocalin family (Cohen and Sundaram, 2020; Cohen et al., 2020; Forman-Rubinsky et al., 2017; Kelley et al., 2015; Sapio et al., 2005; Vuong-Brender et al., 2017).

Nuclear hormone receptor (NHR) transcription factors are key regulators of molting in insects and nematodes (King-Jones and Thummel, 2005; Taubert et al., 2011). NHRs are characterized by a ligand-binding domain (LBD) that has the potential to bind small molecules such as ligands and dietary-derived metabolites (Taubert et al., 2011). NHRs have a canonical zinc-finger DNA-binding domain (DBD) with an unstructured hinge region between the DBD and LBD that is subject to post-translational regulation (Antebi, 2015; Campbell et al., 2008; Ward et al., 2013). A single, conserved nuclear hormone receptor, NHR-23 (also known as NR1F1; hereafter referred to as NHR-23), which is an ortholog of DHR3 in insects and of RORα in mammals, is a key regulator of *C. elegans* molting. NHR-23 is also necessary for spermatogenesis (Ragle et al., 2020, 2022). *nhr-23* mutation or inactivation by RNAi leads to embryonic lethality, larval arrest, ecdysis defects and morphology defects (Frand et al., 2005; Gissendanner et al., 2004; Kostrouchova et al., 1998, 2001). *nhr-23* mRNA expression oscillates over the course of each larval stage, peaking at mid-larval stage and falling at the molt stage (Gissendanner et al., 2004; Kostrouchova et al., 2001). *nhr-23* is necessary for all four larval molts and it regulates microRNAs such as *let-7* and *lin-4* (Kostrouchova et al., 1998, 2001; Patel et al., 2022; Kinney et al., 2023 preprint). *let-7* also regulates *nhr-23,* suggesting that a feedback loop might coordinate molting with developmental timing (Patel et al., 2022). The NHR-23 insect ortholog (DHR3) is part of the molting gene regulatory network (Lam et al., 1997; Ruaud et al., 2010), and the mammalian ortholog (RORα) regulates circadian rhythms, lipid metabolism and immunity (Jetten, 2009). However, how NHR-23 regulates molting and whether it is part of the core oscillator that promotes rhythmic gene expression over each larval stage is poorly understood (Tsiairis and Großhans, 2021).

We show here that NHR-23 protein oscillates and rapid NHR-23 depletion via an auxin-inducible degron causes severe developmental delay and death. Analysis of NHR-23 target genes suggests a role in coordinating aECM assembly, remodeling and construction of specific cuticular structures. NHR-23 depletion causes aberrant localization of the collagens ROL-6 and BLI-1, and of the pre-cuticle factor NOAH-1. NHR-23 activity is necessary in seam and hypodermal cells to promote molting and timely development. Our work reveals when and where NHR-23 acts to promote molting.

## RESULTS

### NHR-23 protein oscillates during development

**Fig. 1.**
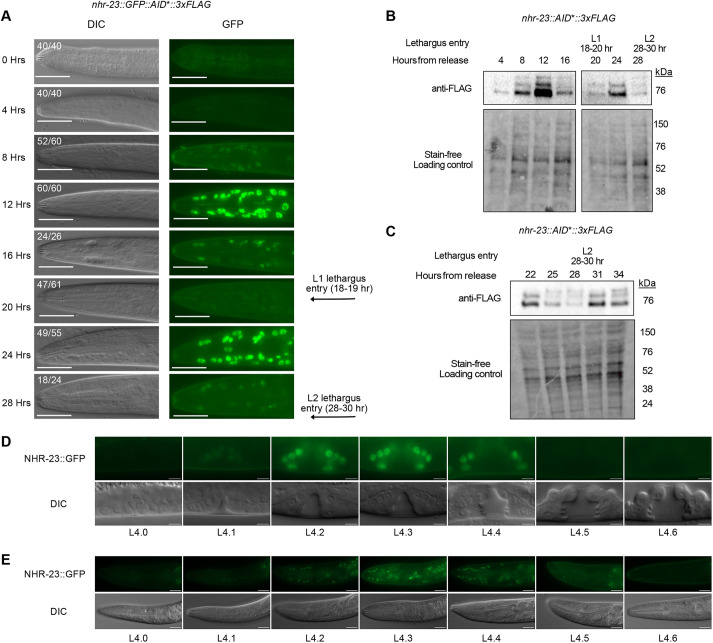
**NHR-23 protein oscillates during development.** (A) Representative images from a timecourse monitoring the expression of endogenous NHR-23::GFP protein. Two biological replicates were performed and the images are representative of the number of animals indicated at each timepoint. Scale bars: 20 µm. (B,C) Anti-FLAG immunoblot analysis of synchronized nhr-23::AID*::3xFLAG animals monitoring the expression of NHR-23 3xFLAG across two timecourses: 4-28 h (B) and 22-34 h (C). Stain-free analysis, which visualizes total protein on the membrane, is provided as a loading control. The blots are representative of three experimental replicates. The times at which animals were observed to enter lethargus is indicated in A-C. (D,E) NHR-23::GFP protein expression in the vulva (D) and head (E) during the L4 larval stage. Animals were staged based on vulval morphology (Mok et al., 2015). Images are representative of 20 animals examined over four independent experiments. Scale bars: 5 µm in D; 20 µm in E. nhr-23::GFP::AID*::3xFLAG (A,C,E) and nhr-23::AID*::3xFLAG (B,C) are previously described endogenous knock-ins that produce C-terminal translational fusions to all known *nhr-23* isoforms (Ragle et al., 2020; Zhang et al., 2015).

Expression of *nhr-23* mRNA oscillates throughout each *C. elegans* larval stage (Gissendanner et al., 2004; Hendriks et al., 2014; Kostrouchova et al., 2001; Meeuse et al., 2020). However, mRNA expression profiles do not always correlate with protein levels (de Sousa Abreu et al., 2009; Vogel and Marcotte, 2012). To determine whether NHR-23 protein oscillates, we monitored the expression of an endogenously tagged NHR-23::GFP, over 28 h (*wrd8* allele; Ragle et al., 2020). During L1, oscillating expression of NHR-23::GFP was observed in the nuclei of seam and hypodermal cells (Fig. S1). Expression of NHR-23 rises and falls before the completion of the 1st molt (Fig. 1A). As GFP tags can affect the expression or stability of proteins (Agbulut et al., 2006; Baens et al., 2006), we assayed the expression of NHR-23 through western blotting time-courses using an nhr-23::AID*::3xFLAG strain (*kry61* allele; Zhang et al., 2015). These experiments confirmed that NHR-23::AID*::3xFLAG also oscillates (Fig. 1B,C), similar to our NHR-23::GFP imaging experiments (Fig. 1A). We detected three distinct NHR-23 bands; the lowest band is consistent with the size of NHR-23b/f (Fig. 1B,C, Fig. S2). The upper two bands were larger than expected, which could reflect post-translational modification or that NHR-23 migrates aberrantly during SDS-PAGE. All observed NHR-23 bands oscillated, although the lowest band was expressed more strongly than the other bands (Fig. 1B,C). The peak in NHR-23 expression was earlier in each larval stage than previous qRT-PCR approaches suggested (Gissendanner et al., 2004; Kostrouchova et al., 2001) and more in line with recent RNA-seq and imaging data (Meeuse et al., 2020; Kinney et al., 2023 preprint). To understand how NHR-23 promotes molting, it is important to clarify when it peaks in expression. We examined NHR-23::GFP expression over the course of the 4th larval stage, as vulva morphology allows for more precise sub-staging (Mok et al., 2015). NHR-23::GFP expression was first detectable in L4.1 larvae, peaked in expression from L4.2-L4.3, disappeared in the vulva by L4.5 and in the head by L4.6, and then remained undetectable (Fig. 1D,E, Fig. S1B,C), similar to what the observations of Kinney et al. (2023) of NHR-23::mScarlet.

### NHR-23 depletion causes severe developmental delay

**Fig. 2.**
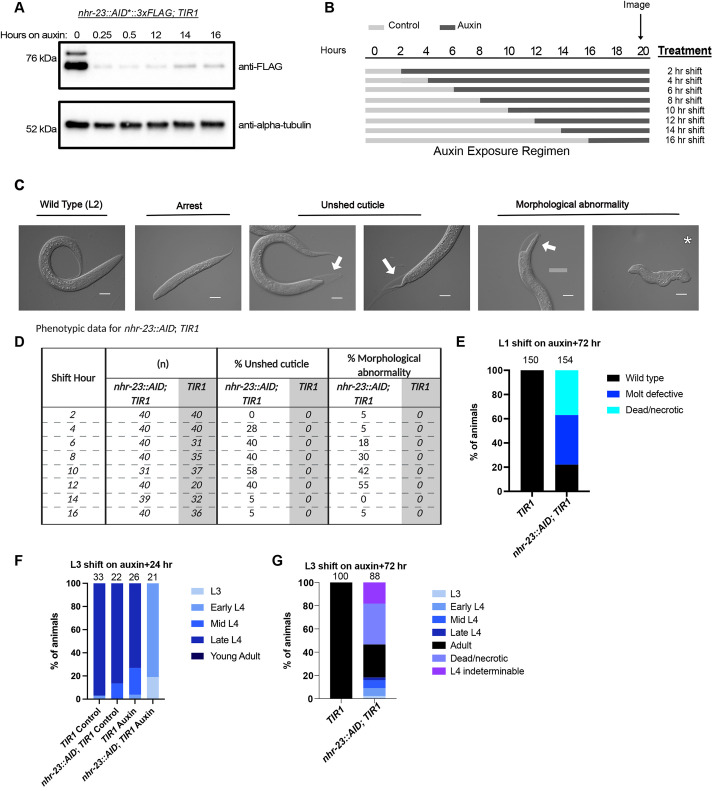
**Distinct phenotypes are observed when NHR-23 is depleted early or late in L1 larvae.** (A) An anti-FLAG immunoblot to monitor NHR-23 depletion. Synchronized nhr-23::AID*::3xFLAG; TIR1 and *TIR1* animals were grown on MYOB plates for 12 h and then shifted onto control or auxin plates. Lysates were collected at the indicated time points. An anti-α-tubulin immunoblot is provided as a loading control. The blots are representative of three experimental replicates. (B) Schematic of the experimental set-up. Synchronized L1 larvae were grown on control media (light-gray bands) and transferred to 4 mM auxin (dark-gray bands) every 2 h. Animals remained on auxin until they were imaged at 20 h post-release. (C) Representative images of phenotypes observed when nhr-23::AID*::3xFLAG; TIR1 animals were exposed to auxin in the first larval stage. (D) Percentage of animals of the indicated genotypes with unshed cuticles and morphological abnormalities after exposure to auxin. The number of animals scored (*n*) is provided. (E) Synchronized *nhr-23::AID; TIR1* L1 larvae were released on auxin and scored for molting defects and death 72 h post-release. (F) Animals of the indicated genotype were synchronized and shifted onto auxin at 25 h post-release and imaged 23 h later (48 h post-release). Animals were staged based on vulval morphology (Mok et al., 2015). L4 larval stages were grouped as early L4 (4.0-4.2), mid L4 (4.3-4.5) and late L4 (4.6-4.9). (G) *nhr-23::AID; TIR1* animals were treated as in F and scored after 72 h on auxin. The number of animals assayed for each genotype and condition is indicated at the top of the graphs in E-G.

Given that NHR-23 oscillates, we tested when NHR-23 was necessary for molting during the L1 larval stage. In a pilot experiment, we consistently found weaker depletion phenotypes using an nhr-23::GFP::AID*::3xFLAG strain (Ragle et al., 2020) compared with the nhr-23::AID*::3xFLAG strain (Zhang et al., 2015) (Table S1). We therefore transitioned to using the nhr-23::AID*::3xFLAG strain for all experiments involving phenotypic analysis. We first tested NHR-23::AID*::3xFLAG depletion kinetics and found robust depletion within 15 min of exposure to 4 mM auxin; levels remained low over the 16 h timecourse (Fig. 2A).

We performed timed depletion experiments in the L1 larval stage (Fig. 2B) using an *eft-3p::TIR1::mRuby2* control strain (henceforth referred to as *TIR1*) and a strain that permits somatic depletion of NHR-23 in the presence of auxin (eft-3p::TIR1::mRuby2; nhr-23::AID*::3xFLAG; henceforth referred to as *nhr-23::AID*; *TIR1*). L1 larvae were synchronized by starvation arrest and release onto MYOB plates. Animals were shifted onto either 4 mM auxin or control plates every 2 h and scored for molting defects at 20 h post-release from starvation (Fig. 2B). *TIR1* control animals shifted onto control or auxin and *nhr-23::AID*; *TIR1* animals shifted onto control plates all reached the L2 stage (Fig. 2C,D). In contrast, *nhr-23::AID*; *TIR1* animals shifted onto auxin displayed multiple phenotypes, including molting defects, internal vacuoles and developmental abnormalities such as disrupted tails and viable animals with a squashed morphology (Fig. 2C). We then quantified the molting defects, scoring animals unable to shed their cuticles or with morphological abnormalities (Fig. 2D). Animals shifted onto auxin within the first 4 h post-release tended to arrest as L1 larvae with few molting or morphological defects (Fig. 2D). To determine at what stage these animals arrested, we used an *hlh-8p::GFP* promoter reporter to monitor the M cell lineage (Harfe et al., 1998). Newly hatched L1 animals have one M cell that undergoes a stereotypical series of divisions to produce 16 M lineage cells by the L1 molt (Fig. S3A) (Sulston and Horvitz, 1977). *nhr-23::AID*; *TIR1* animals shifted onto auxin at 3 h post-release were late reaching L1 based on M cell number (Fig. S3B,C). When NHR-23 animals were scored 72 h post-auxin shift, we observed molting defects and necrotic animals (Fig. 2E, Fig. S4A), suggestive of a severe developmental delay. We also observed arrested animals with a wild-type morphology that were likely L1 larvae by size (Fig. 2E). Shifts between 6 and 12 h post-release resulted in increased morphological and molting defects (Fig. 2D). Animals shifted onto auxin at 9 h reached the end of the L1 stage by M cell number (Fig. S3B,C). Shifts at 14 and 16 h post-release resulted in wild-type L2 animals, as judged by size (Fig. 2D).

To test whether NHR-23 was similarly required in other larval stages, we performed depletion experiments later in development during which we could use vulval morphology to score progression through the L4 stage. We performed depletion experiments shifting early L3 animals onto auxin and monitored development by scoring vulva morphology 23 h later. The majority of *TIR1* animals grown on control or auxin plates and *nhr-23::AID*; *TIR1* animals grown on control plates reached late L4, with a fraction of the population in early or mid-L4 (Fig. 2F, Fig. S4B). In contrast, when *nhr-23::AID*; *TIR1* animals were shifted to auxin, the vast majority of animals were early L4 larvae with a fraction of the population remaining in L3 (Fig. 2F, Fig. S4B). Repeating these experiments scoring later timepoints revealed that NHR-23-depleted animals were slowly progressing through development. After 72 h on auxin, we observed adult animals, as evidenced by the presence of oocytes (Fig. 2G, Fig. S4C), while all control animals were adults. A subset of NHR-23-depleted L4 larvae could not be precisely staged due to aberrant vulval morphology (Fig. S4C). These animals had large vulval lumens reminiscent of a 4.3 stage vulva (Mok et al., 2015), but we also observed adults with a similar large vulval lumen (Fig. S4C). A fraction of *nhr-23::AID*; *TIR1* animals grown for 72 h on auxin were dead and appeared necrotic, with large fluid filled spaces within the animal (Fig. 2G, Fig. S4C). These depletion experiments indicate that NHR-23 is required for timely developmental progression through multiple larval stages, consistent with previous reports of *nhr-23* inactivation by RNAi (Macneil et al., 2013; Patel et al., 2022).

### NHR-23 regulates oscillating genes involved in aECM biogenesis and regulation of protease activity

**Fig. 3.**
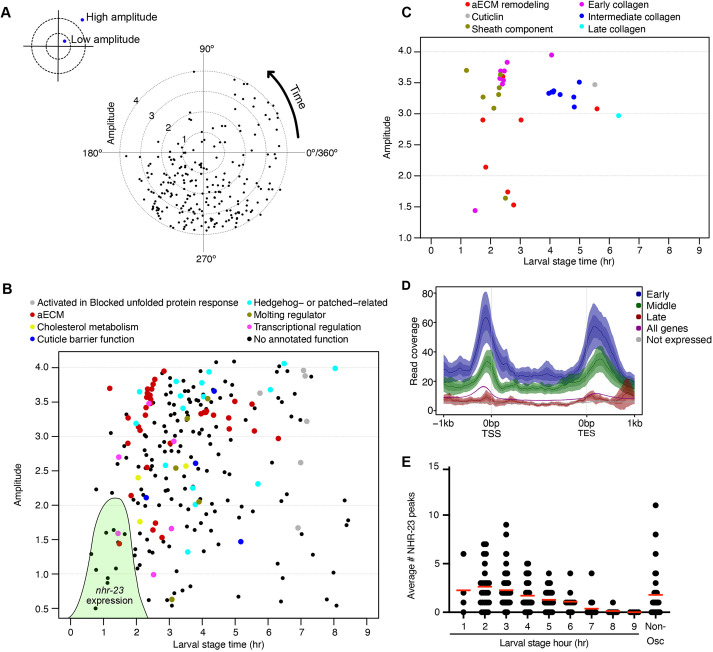
**NHR-23 regulates oscillating genes involved in aECM biogenesis.** (A) Radar chart plotting amplitude over the phase of peak expression of *nhr-23*-regulated genes from Kouns et al. (2011). The dotted circles indicate amplitude, with the innermost circle representing an amplitude of 1 and the outermost circle representing an amplitude of 4. Created with Biorender.com. (B) The data from A plotted as a scatter plot and functionally annotated (Table S2). We converted the 360° oscillation to developmental timing over a 9 h larval stage so that 1 hour=40°. The green shaded area represents NHR-23 expression based on RNA-seq (peak phase=178.11°, amplitude=2.11; Meeuse et al., 2020), imaging (Fig. 1A,D,E) and western blotting data (Fig. 1B,C). (C) The genes in the aECM component class were subdivided into more specific classes and plotted as in B. (D) Average signal from NHR-23 ChIP-seq data (Gerstein et al., 2010) for *nhr-23*-regulated oscillating genes with peak phases at 135°-254.9° (early), 255-14.9° (middle) and 15°-134.9 (late). Expression of *nhr-23* mRNA peaks at 178.11°. Signal is plotted relative to transcription start site (TSS) and transcription end site (TES). The average NHR-23 signal for all 21,600 *C. elegans* genes (all genes) and the 20% of genes with the lowest expression (no expression) are shown for reference. The mean signal is plotted with a line and the 95% confidence interval of the mean is indicated by the shaded area. (E) Number of NHR-23 peaks flanking and within *nhr-23-*regulated genes. The genes are binned by their peak phase relative to adjusted larval stage time. Non-oscillating (non-osc) *nhr-23* genes are also depicted.

To gain insight into how NHR-23 depletion causes developmental delay, we analyzed the expression of *nhr*-23-regulated genes by mining existing microarray (Kouns et al., 2011) and oscillatory gene expression (Meeuse et al., 2020) datasets (Table S2). Of the 265 *nhr-23-*regulated genes in the microarray dataset, 236 (89%) were oscillatory with the bulk of genes peaking in expression between 180° and 360° (Fig. 3A, Table S2); *nhr-*23 mRNA peaks at 178.11° (Meeuse et al., 2020). A similar trend was observed in a recent analysis of these datasets (Tsiairis and Großhans, 2021). In contrast, 10-20% of *C. elegans* genes oscillate in expression (Hendriks et al., 2014; Kim et al., 2013; Meeuse et al., 2020) (two-tailed χ2 test *P*<0.0001). *nhr-23-*regulated oscillating genes were enriched in gene ontology classes such as ‘cuticle structure’, ‘regulation of endopeptidases’ and ‘metalloprotease activity’ (Table S3). These gene ontology classes are a specific subset of the functions enriched in all oscillating genes (Table S4). To provide biological context, we converted the 360° period to developmental time, assuming a 9 h larval stage and set the molt from the start of lethargus (45°) to the end of ecdysis (135°) as in Meeuse et al. (2023). Most *nhr-23*-regulated genes involved in aECM structure/function, cholesterol metabolism, molting regulation, transcriptional regulation and signal transduction had peak amplitudes within 3 h of the *nhr-23* expression peak (Fig. 3B). Genes involved in the blocked protein unfolding response, as well as some aECM genes, peaked later in each larval stage (Fig. 3B). Although the *C. elegans* genome encodes 181 collagen genes (Teuscher et al., 2019), *nhr-23* only regulated 13 collagens, including all of the furrow collagens implicated in epidermal damage sensing; these furrow collagens are in the ‘early’ collagen group (*dpy-2*, *dpy-3*, *dpy-7* and *dpy-10*; Table S2, Fig. 3C) (Johnstone and Barry, 1996; Page and Johnstone, 2007; Dodd et al., 2018). Other *nhr-23-*regulated intermediate collagens have well-described roles in body morphology (*sqt-1*, *sqt-2* and *rol-6*; Table S2, Fig. 3C) (Kramer and Johnson, 1993; Johnstone and Barry, 1996; Page and Johnstone, 2007). There are also several sets of genes involved in aECM biogenesis and remodeling, such as lipocalins, proteases, protease inhibitors, fibrillin, PAN and zona pellucida (ZP) domain-containing proteins, and leucine-rich repeat proteins (Fig. 3C). Although not enriched as a gene ontology class, NHR-23 also regulates several transcription factors implicated in molting or energy metabolism (*peb-1*, *nhr-91* and *dpy-20*; Table S2, Fig. 3B) (Clark et al., 1995; Fernandez et al., 2004; Kasuga et al., 2013).

To explore direct targets of NHR-23, we analyzed an NHR-23 L3 ChIP-seq dataset (Gerstein et al., 2010). As expected, NHR-23 was enriched in the promoter region of genes (Fig. 3D, Fig. S5, Table S5). There was also notable enrichment downstream of the transcriptional end site (Fig. 3D, Fig. S5, Table S5). We then examined whether NHR-23 was enriched near *nhr-23-*regulated oscillating genes (Table S2). Genes that peaked in expression in the hour after the *nhr-23* mRNA peak in expression (Fig. 3E; hour 2) had the highest average number of NHR-23 peaks flanking and within their gene body. The average number of NHR-23 peaks flanking and within gene bodies declined, and few peaks were detected flanking genes between larval stage hours 7-9 (Fig. 3E). Genes that peak in expression close to when *nhr-23* mRNA levels peak tend to have higher NHR-23 levels upstream, downstream and within their gene bodies (Fig. 3D, early genes).

**Fig. 4.**
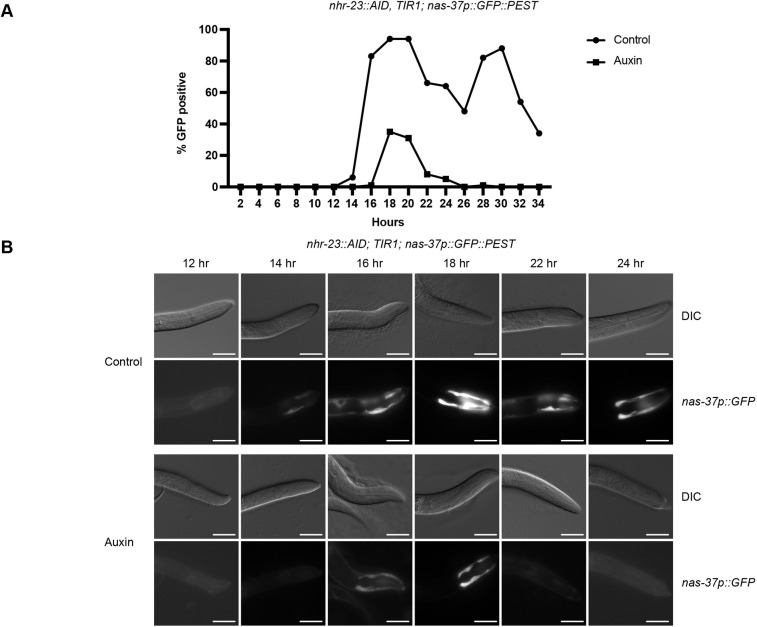
**NHR-23 depletion causes reduced expression of a *nas-37p::GFP::PEST* promoter reporter.** (A) *nas-37p::GFP::PEST* expression timecourse. Synchronized *nhr-23::AID, TIR1; nas-37p::GFP::PEST* L1 larvae were released on control or auxin plates and scored for head or hypodermal GFP expression every 2 h. Fifty animals per time point were scored in two independent experiments and the percentage of animals expressing GFP are presented. (B) Representative images of *nhr-23::AID, TIR1; nas-37p::GFP::PEST* animals grown on control or auxin plates at the indicated time points. Scale bars: 20 µm.

As NHR-23-regulated oscillating genes were enriched in proteases and protease inhibitors (Table S2), we tested how NHR-23 depletion affected expression of a *nas-37* promoter reporter; *nas-37* is a protease implicated in *C. elegans* ecdysis (Davis et al., 2004). We released synchronized *nhr-23::AID, TIR1* L1 larvae carrying a *nas-37p::GFP::PEST* reporter onto control and auxin plates, and monitored GFP expression over development. Animals growing on control media displayed oscillating reporter activity with peaks at 18 and 30 h (Fig. 4A,B). NHR-23 depleted animals exhibited a delayed onset of reporter expression and only a subset of animals expressed the reporter (Fig. 4A,B). There was only one pulse of *nas-37p::GFP::PEST* expression, after which we detected no reporter activity (Fig. 4A,B). Together, these data confirm that NHR-23 regulates *nas-37* expression and that, following NHR-23 depletion, there is a weaker pulse of target gene expression followed by a failure to express again.

### NHR-23 is necessary for NOAH-1 localization to the aECM

**Fig. 5.**
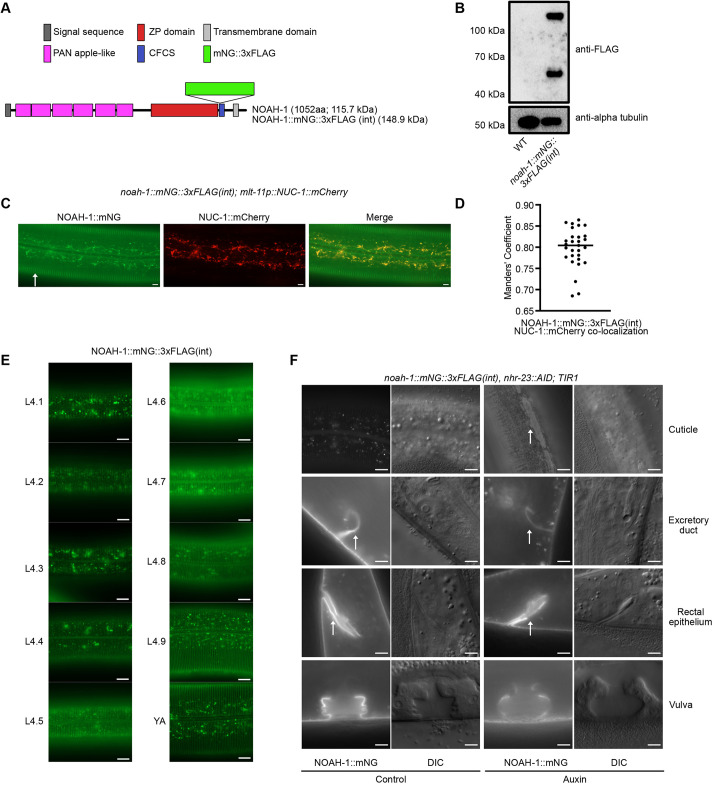
**NHR-23 depletion causes defective NOAH-1 localization.** (A) Domain prediction for NOAH-1. ZP, zona pellucida; CFCS, consensus furin cleavage site. Predicted size of NOAH-1 and NOAH-1 with an internal mNeonGreen::3xFLAG is provided in kDa. (B) Anti-FLAG immunoblots on lysates from *noah-1::mNG::3xFLAG(int)* mid-L4 animals. An age-matched wild-type control is included in each experiment. An anti-α-tubulin immunoblot was used as a loading control. (C) Representative images from a *noah-1::mNG::3xFLAG(int); mlt-11p::NUC-1::mCherry* mid-L4 animal. Arrow indicates cuticle localization. Scale bars: 5 µm. (D) Manders' co-localization analysis of NOAH-1::mNG::3xFLAG(int) and the lysosome marker NUC-1::mCherry. Three biological replicates were performed analyzing a total of 29 animals. The horizontal line indicates the median. (E) NOAH-1::mNG expression timecourse through L4; animals were staged by vulva morphology (Mok et al., 2015). 100 animals were examined over three independent replicates. Scale bars: 10 µm. (F) Representative NOAH-1::mNG and DIC images of the indicated tissues of *noah-1::mNG::3xFLAG(int)* and *nhr-23::AID; TIR1* animals grown on control or auxin plates. Arrows indicate aberrant NOAH-1::mNG localization in the aECM above the seam cells, and wild-type localization to excretory duct and rectum. Two biological replicates were performed; the images represent 100% of animals scored (*n*=51 for control and *n*=44 for auxin). Scale bars: 20 µm for all images except the *noah-1::mNG* excretory duct, rectal epithelium and vulva.

Inactivation of the *nhr-23*-regulated predicted protease inhibitor gene *mlt-11* produces embryonic disorganization with accompanying lethality reminiscent of *noah-1* mutants (Ragle et al., 2022; Vuong-Brender et al., 2017). NOAH-1 is a zona pellucida domain protein that is part of the pre-cuticular aECM in embryos and larvae, a transient structure thought to play a role in patterning the cuticle and in embryonic elongation (Cohen and Sundaram, 2020). We introduced an mNeonGreen::3xFLAG (mNG::3xFLAG) tag after the ZP domain, which is similar to the insertion site for a previously generated mCherry knock-in (Fig. 5A; Vuong-Brender et al., 2017). *noah-1::mNG::3xFLAG(int)* animals had a wild-type brood size (Fig. S6A) and in *noah-1::mNG::3xFLAG(int), nhr-23::AID; TIR1* lysates there was a band of the expected full-length protein size (∼150 kDa) as well as a lower ∼50 kDa band (Fig. 5B). *noah-1* is only predicted to have a single isoform so this might reflect a cleavage or degradation product. ZP proteins are frequently cleaved after their ZP domain (Gupta, 2021; Kiefer and Saling, 2002). Although there is a predicted furin RXXR cleavage site at the start of the CFCS domain, the smaller isoform is consistent with cleavage immediately after the ZP domain (Fig. 5A,B). It will be valuable in the future to create isoform-specific mNeonGreen knock-ins to determine where each localizes. NOAH-1::mNG was observed in the cuticle, and in punctate and tubular structures in the hypodermis (Fig. 5C). Hypodermal NOAH-1::mNG significantly overlapped with the lysosomal marker NUC-1::mCherry (Fig. 5C,D) (Guo et al., 2010; Clancy et al., 2023). NOAH-1::mNG was detected in lysosomes and weakly in the aECM from L4.1-L4.4, and then reached its maximum aECM intensity between L4.5 and L4.7, localizing to thick bands reminiscent of annuli (Fig. 5E). aECM expression decreased from L4.8 to young adulthood (Fig. 5E). NOAH-1::mNG also localized to alae from L4.6 onwards, similar to reports for NOAH-1::sfGFP (Katz et al., 2022).

We next examined the effect of NHR-23-depletion on NOAH-1 expression and localization. In L4 larvae, we observed the expected vulval lumen localization and aECM expression (Cohen et al., 2020; Katz et al., 2022), as well as localization to the rectum and excretory duct (Fig. 5F). NHR-23 depletion did not affect NOAH-1 localization in the excretory duct or rectum (Fig. 5F). Although vulval morphology is aberrant in NHR-23-depleted animals, NOAH-1 still localizes to cell surfaces and to lysosomes (Fig. 5F). In mid-L4, NOAH-1 is normally localized to the cuticle and lysosomes, and weakly localizes to alae (Fig. 5F). NHR-23 depletion caused a loss of NOAH-1::mNG localization to annuli, the formation of small irregular punctae and accumulation of NOAH-1::mNG in the aECM over the seam cells (Fig. 5F). Together, these data indicate that NHR-23 is dispensable for NOAH-1 expression, but necessary for correct localization in the cuticle.

### NHR-23 depletion causes defects in aECM structure and function

**Fig. 6.**
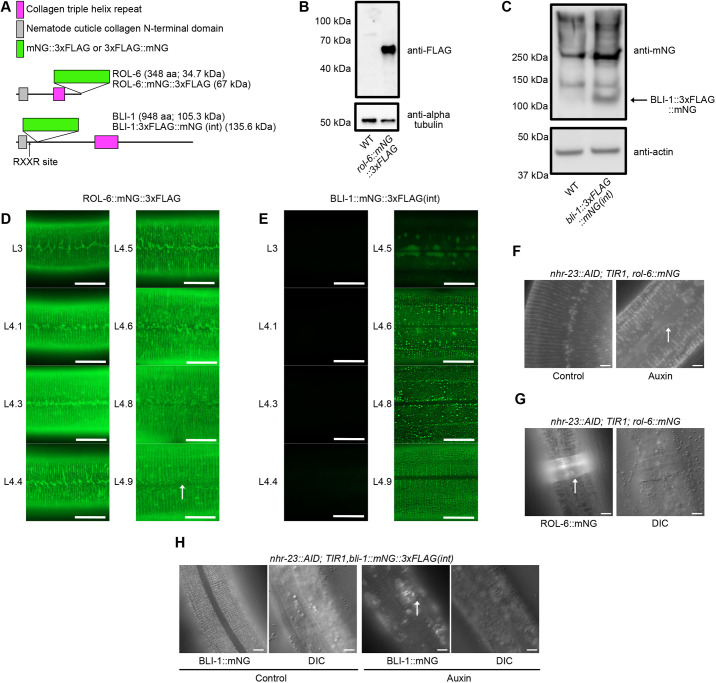
**NHR-23-depletion causes ROL-6 and NOAH-1 localization defect, and reduced expression and mislocalization of BLI-1.** (A) Cartoon of ROL-6 and BLI-1 domains with knock-in position of mNG::3xFLAG or 3xFLAG::mNG tags. Predicted protein size is provided in kDa. (B,C) Immunoblots on lysates from *rol-6::mNG::3xFLAG* mid-L4 animals (B) and *bli-1::3xFLAG::mNG(int)* mid-L4 animals (C). An age-matched wild-type control is included in each experiment. An anti-α-tubulin (B) or anti-actin (C) immunoblot was used as a loading control. For the blots in C, the anti-mNeonGreen blot was performed on a soluble cuticle fraction and the anti-actin control was performed on a soluble intracellular fraction (see Materials and Methods). An arrow indicates the position of the BLI-1::3xFLAG::mNG band. (D) ROL-6::mNG and (E) BLI-1::mNG expression timecourse through L4; animals were staged by vulva morphology (Mok et al., 2015). One hundred animals were examined over three independent replicates. Scale bars: 10 µm. Arrow indicates fibrous localization pattern in D. (F) Representative images of *nhr-23::AID; TIR1, rol-6::mNeonGreen::3xFLAG* (*rol-6::mNG*) animals grown on control or auxin plates. Arrow indicates mislocalized ROL-6::mNG in the aECM above the seam cells. Images from control and auxin-treated animals are representative of 40/40 animals observed in three independent experiments. (G) Representative images of *nhr-23::AID; TIR1, rol-6::mNG* animals grown on auxin with a corset phenotype. Corset is indicated by an arrow. 24/40 animals displayed this phenotype. (H) Representative images of *nhr-23::AID; TIR1, bli-1::mNeonGreen::3xFLAG* (*bli-1::mNG*) animals grown on control or auxin plates. Three experimental replicates were performed. Control images are representative of 42/42 animals scored. For auxin-treated animals, 38/38 exhibited dim expression of BLI-1::mNG and 30/38 had patchy BLI-1::mNG localization (arrow). Scale bars: 20 µm in F-H.

Given the aberrant NOAH-1 localization (Fig. 5) and *nhr-23*-mediated regulation of early and intermediate collagens (Table S2), we next examined whether NHR-23 depletion affected aECM structure. We used CRISPR/Cas9-mediated genome editing in *nhr-23::AID; TIR1* animals to introduce mNG::3xFLAG cassettes into the intermediate collagen *rol-6* (C-terminal translational fusion) and *bli-1*, a collagen that forms struts in the adult cuticle (internal translational fusion) (Fig. 6A). We also introduced a 3xFLAG::mNG into *bli-1* at the same location as the mNG::3xFLAG knock-in. *rol-6* is regulated by *nhr-23*, whereas *bli-1* is an adult-specific collagen expressed in L4 larvae, so its expression would not be interrogated in the microarray experiment (Table S2) (Kouns et al., 2011). We first confirmed that each knock-in had a wild-type brood size (Fig. S6A) and then tested fusion protein size by western blotting. Anti-FLAG immunoblotting on *nhr-23::AID; TIR1, rol-6::mNG::3xFLAG* lysates detected a single band of the expected size (∼70 kDa; Fig. 6B). In *nhr-23::AID; TIR1, bli-1::3xFLAG::mNG*, there was a single band consistent with N-terminal processing of a monomer at an RXXR furin cleavage site that would remove 10 kDa (125 kDa predicted size). We could only detect protein by extracting soluble cuticle components. BLI-1::3xFLAG::mNG and BLI-1::mNG::3xFLAG had identical localization to one another (Fig. S6B), and were similar to an extrachromosomal transgenic BLI-1::GFP reporter (Tong et al., 2009); all subsequent experiments were performed using *bli-1::mNG::3xFLAG(int)*. Further details of BLI-1::mNG localization will be described elsewhere (Adams et al., submitted J. Adams and A.D.C., unpublished).

We first characterized expression of each translational fusion during the 4th larval stage, using vulval morphology to stage animals (Mok et al., 2015). ROL-6::mNG was first detected in thin bands reminiscent of furrows with a jagged pattern over seam cells (L4.1-L4.3; Fig. 6D). In L4.4-L4.5, some thicker aggregations began appearing; by L4.6-L4.9, ROL-6::mNG relocalized to thicker bands reminiscent of annuli (Fig. 6D). In L4.9, a fibrous pattern could also be observed (Fig. 6D). BLI-1::mNG expression was not detected until L4.5, when it was detected in hypodermal cells (Fig. 6E). In L4.4-L4.8, BLI-1::mNG was observed in a punctate localization in rows with some irregular brighter punctae (Fig. 6E). By L4.9, BLI-1::mNG was found in rows of regularly spaced punctae and was consistently excluded from the cuticle over seam cells throughout L4 and adulthood (Fig. 6E).

To examine the impact of NHR-23 depletion on BLI-1 and ROL-6 localization, we shifted *nhr-23::AID; TIR1; rol-6::mNG* or *nhr-23::AID; TIR1, bli-1::mNG* animals onto control or auxin plates in early L3. We examined animals after 23 h on control plates when animals were mid-late L4s. We scored NHR-23-depleted animals after 47 h on auxin plates due to the developmental delay; this approach ensured that control- and auxin-treated animals were stage matched. ROL-6::mNG expression appeared unaffected by NHR-23-depletion, but the furrow localization was irregular and thicker, and we observed gaps in the annular ROL-6::mNG over the seam cells (Fig. 6F). Many animals had a corset of unshed cuticle to which ROL-6::mNG localized (Fig. 6G). We next addressed whether NHR-23 depletion affected the formation of struts in the cuticle medial layer. NHR-23 depletion caused reduced expression of BLI-1::mNG and a loss of the punctate pattern (Fig. 6H). BLI-1::mNG weakly localized to annuli and animals displayed bright disorganized patches of BLI-1::mNG in the cuticle (Fig. 6H). BLI-1::mNG expression appeared dimmed in the *nhr-23::AID; TIR1* background compared with a wild-type background, which would be consistent with low level auxin-independent NHR-23 depletion (Fig. S6B).

**Fig. 7.**
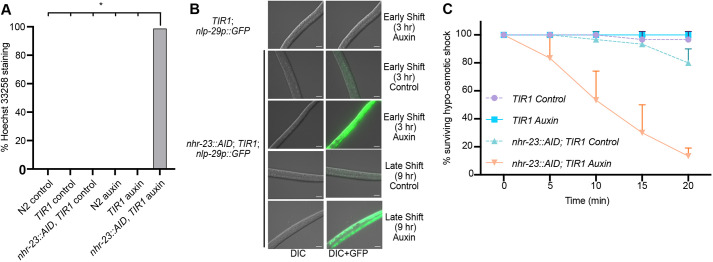
**Depletion of NHR-23 leads to a defective cuticle barrier.** (A) Permeability to Hoechst 33258 dye. Synchronized animals of the indicated genotype were shifted onto control or auxin plates at 25 h post-release and grown for 23 h. Animals were washed off plates and incubated with the cuticle impermeable/membrane permeable Hoechst 33258 dye and scored for nuclear staining in the body. The data are from two biological replicates. *P<0.000001 (two-tailed Student's *t*-test). (B) Representative images of GFP expression in animals of the indicated genotype after an early and late shift to control or auxin plates. The images are representative of 50 animals observed during each of two experimental replicates. (C) *nhr-23::AID; TIR1* and *TIR1* animals were subjected to hypo-osmotic shock and scored every 5 min. Ten animals were assayed for each genotype and condition, with three biological replicates. Data are averages of the three biological replicates±s.d.

Given the defects in aECM structure after NHR-23 depletion, we tested whether the epidermal barrier was intact. First, we incubated control and auxin-treated *nhr-23::AID, TIR1* animals with the cuticle-impermeable, cell membrane-permeable Hoechst 33258 dye, which stains nuclei. N2 and *TIR1* animals grown on control or auxin plates and *nhr-23::AID, TIR1* animals grown on control plates exhibited no staining, whereas almost all animals on auxin plates displayed Hoechst staining (Fig. 7A). Animals shifted onto auxin early or late in L1 robustly expressed an *nlp-29p::GFP* reporter (Fig. 7B, Fig. S7), which can be activated by infection, acute stress and physical damage to the cuticle (Pujol et al., 2008; Zugasti and Ewbank, 2009). We further tested the cuticle integrity in a hypo-osmotic shock assay in which animals with defective cuticle barriers rapidly die after exposure to water (Kage-Nakadai et al., 2010). *TIR1* animals grown on control media or auxin were completely viable in water (Fig. 7C). *nhr-23::AID, TIR1* control animals had a weakly penetrant sensitivity to hypo-osmotic shock, while the same strain grown on auxin rapidly died after hypo-osmotic shock (Fig. 7C). These data suggest that NHR-23 is required for cuticle barrier establishment or maintenance.

### *nhr-23* is necessary in seam and hypodermal cells for larval development

**Fig. 8.**
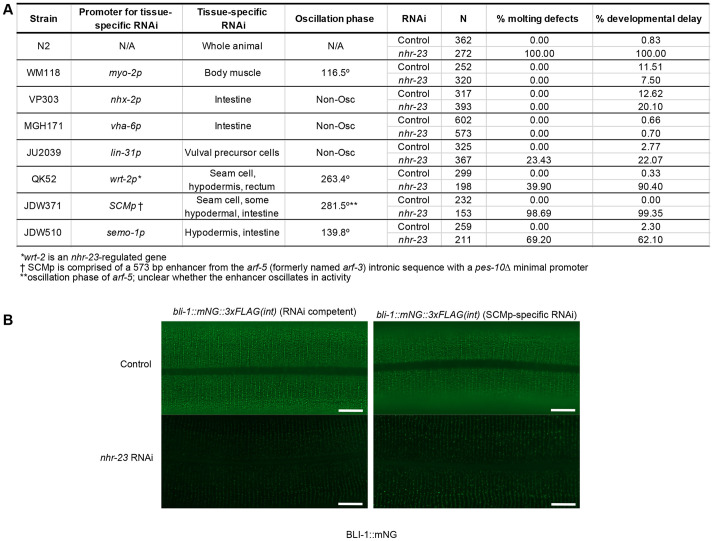
***nhr-23* is necessary in seam cells for developmental progression, molting and BLI-1 localization.** (A) Tissue-specific RNAi. A timed egg lay of animals of the indicated strains was performed on control or *nhr-23* RNAi plates and plates were scored 3 days later. The promoters used to reconstitute RNAi, their tissue specificity and oscillation phase (when applicable) are provided. The number of progeny scored from two biological replicates is indicated. Developmental delay was scored as a failure to reach adulthood after 72 h of growth. Molting defects included animals trapped in cuticles, animals dragging cuticles and animals with cuticle corsets. (B) L2 *bli-1::mNG* (RNAi competent) and *bli-1::mNG; SCMp::rde-1; rde-1(ne300)* (*SCMp*-specific RNAi) animals were shifted onto control or *nhr-23* RNAi plates at L2 and grown until stage L4.8. Equal exposure times were used for all images. Scale bars: 10 µm. Images are representative of 20 animals over two independent experimental replicates.

Finally, we used a tissue-specific RNAi approach to determine in which cells *nhr-23* was necessary for molting. In RNAi-proficient N2 animals, 100% of *nhr-23(RNAi)* animals exhibited developmental delay and molting defects (Fig. 8A). *nhr-23* knockdown in body-wall muscle and intestinal cells produced no molting defects and we observed only mild developmental delay (Fig. 8A). Vulval precursor cell-specific *nhr-23(RNAi)* caused moderate molting defects and developmental delay (Fig. 8A). We observed highly penetrant developmental delay and molting defects when we performed *nhr-23* RNAi in a strain using a *wrt-2* promoter for tissue-specific RNAi (Fig. 8A). *wrt-2* is expressed in seam cells, rectal cells and hypodermal cells (Fig. S8A,B; Aspöck et al., 1999). Given this result and the defective ROL-6::mNG and NOAH-1::mNG (Figs 5 and 6) localization over seam cells, we generated more tissue-restricted RNAi strains. We used a minimal seam cell-specific enhancer with a *pes-10* minimal promoter (Ashley et al., 2021), which is robustly expressed in seam cells with some weak hypodermal expression (Fig. S8C,D). To test the specificity of this strain, we introduced a *his-72::mNG* knock-in. In animals treated with control RNAi, we observed nuclear expression in seam, hypodermal, intestinal, vulval and germline cells, as expected for a histone H3 fusion (Fig. S9). *mNeonGreen* RNAi caused reduced HIS-72::mNG expression in seam, hypodermal syncytium and intestinal nuclei. *nhr-23(RNAi)* in this *SCMp* RNAi strain phenocopied *nhr-23(RNAi*) in N2 animals with almost all animals exhibiting developmental delay and molting defects (Fig. 8A). Notably, intestine-specific *nhr-23* RNAi did not cause phenotypes (Fig. 8A). We then constructed a hypodermis-specific RNAi strain using the *semo-1* promoter (Kaletsky et al., 2018; Köhnlein et al., 2020). *nhr-23* RNAi in this strain produced molting defects and developmental delay, although with less penetrance compared with our *SCMp-*specific RNAi strain (Fig. 8A). To test the effect of *SCMp*-specific *nhr-23* knockdown on the aECM, we introduced a BLI-1::mNG knock-ins in our seam cell-specific RNAi strain. *nhr-23* depletion in this strain caused reduced BLI-1 levels relative to control RNAi (Fig. 8B). This BLI-1::mNG reduction was comparable with both *nhr-23* RNAi in a wild-type control (Fig. 8B) and with NHR-23 protein depletion in the soma (Fig. 6H). Together, these data indicate that *nhr-23* is necessary in the seam and hypodermal cells for timely developmental progression, completion of molting and aECM formation.

## DISCUSSION

*C. elegans* molting is a powerful system for understanding developmentally programmed aECM regeneration. In this study, we determine when and where NHR-23 acts to promote molting. NHR-23 oscillates and its depletion causes severe developmental delay. *nhr-23*-regulated genes are enriched for cuticle components and protease inhibitors, and NHR-23 is enriched at the transcription start and end sites of target genes. NHR-23 depletion causes aberrant localization of the early collagen ROL-6 and the pre-cuticle component NOAH-1. These cuticle defects are correlated with a loss of the cuticle permeability barrier function. Loss of NHR-23 function in L4 stages also causes severely reduced levels of the adult collagen BLI-1, which is also mislocalized. These cuticle defects are correlated with a loss of the cuticle barrier. Tissue-specific RNAi suggests that *nhr-23* is necessary in seam and hypodermal cells.

### NHR-23 is necessary for cuticle structure and function

NHR-23 binds more robustly in the promoter and transcription end site of genes that peak in expression closer to the *nhr-23* mRNA peak in expression (Fig. 3). One intriguing possibility is that the earlier NHR-23-regulated genes may be more sensitive to NHR-23 levels. Consistent with this model, there appear to be more NHR-23 peaks flanking and within early *nhr-23-*regulated genes (Fig. 3, Tables S2, S5). This model would align with *E. coli* amino acid biosynthesis gene regulation where enzymes earlier in the pathway have more responsive promoters with higher activity (Zaslaver et al., 2004). Interestingly, the non-oscillating *nhr-23-*regulated genes had a range in number of NHR-23 peaks. It is unclear why NHR-23 oscillation is failing to drive oscillation of these target genes. Some possible explanations include more stable mRNA transcripts in comparison with the transcripts of the oscillating genes or combinatorial gene regulation involving a non-oscillating transcription factor.

NHR-23 regulates the expression of *nas-37*, a protease implicated in molting (Fig. 4; Frand et al., 2005). Curiously, two other targets (*noah-1* and *rol-6*) seemed to be expressed at near wild-type levels but displayed aberrant localization (Figs 5 and 6). These data highlight a potential limitation of a single timepoint gene expression study. A shift in phase of an oscillating gene without change in amplitude could create the appearance of up- or downregulation, depending on the timepoint sampled (Tsiairis and Großhans, 2021). The *nhr-23(RNAi)* microarray is a useful starting point to understand how NHR-23 promotes molting, but an RNA-seq timecourse on NHR-23 depleted animals may be necessary to identify regulated genes. Such an approach was necessary to determine how the pioneer factor, BLMP-1, promotes developmental timing (Hauser et al., 2022 preprint).

NHR-23 depletion caused aberrant localization of NOAH-1 and ROL-6, lower levels of BLI-1 and a severe barrier defect (Figs 5-7). There are numerous *nhr-23*-regulated genes that are implicated in the epithelial barrier. The cuticle furrow formed by six *nhr-23-*regulated collagens (*dpy-2*, *dpy-3*, *dpy-7*, *dpy-8*, *dpy-9* and *dpy-10*) is thought to be monitored by a sensor that coordinates several stress responses (Dodd et al., 2018). An RNAi screen of 91 collagens found that inactivation of only these six collagens caused a barrier defect (Sandhu et al., 2021). Inactivation of a subset of the furrow collagens causes permeability to Hoechst 33458 dye and elevated *nlp-29p::GFP* reporter activity (Dodd et al., 2018), consistent with our NHR-23-depletion data (Fig. 7). *bus-8* is a predicted glycosyltransferase that plays a role in the epithelial barrier and is also regulated by *nhr-23*; its peak expression follows that of the furrow collagens (Table S2). Screening *nhr-23*-regulated genes may reveal other genes implicated in the epithelial barrier and the peak phase could provide insight into how this barrier is constructed.

### NHR-23-depletion causes developmental delay and failed molting

What is driving NHR-23-depleted animals to eventually attempt to molt? One model is that NHR-23 depletion is incomplete and the remaining NHR-23 is sufficient to drive low levels of target gene expression. Consistent with this idea, NHR-23 depletion or knockdown results in developmental delay but not an arrest (Fig. 2; MacNeil et al., 2013; Patel et al., 2022). Low sustained NHR-23-levels could eventually allow the accumulation of factors that initiate apolysis but with insufficient expression for execution. Consistent with this idea, an NHR-23-regulated promoter reporter (*nas-37p::GFP::PEST*) peaked at a similar time in control and NHR-23-depleted animals (Fig. 4), but was expressed at lower levels. *nas-37* is necessary for *C. elegans* ecdysis and recombinant NAS-37 promotes the formation of retractile rings, a structure associated with molting, in the parasitic nematode *H. contortus* (Davis et al., 2004). Alternatively, other factors could eventually promote molting. Molting can be uncoupled from the stage-specific developmental events controlled by the heterochronic pathway, which leads to death when animals attempt molting before completion of cell division and differentiation (Ruaud and Bessereau, 2006). Molting is thought to be controlled by a recently discovered oscillator, although the mechanism remains to be fully elucidated (Tsiairis and Großhans, 2021). It is possible that other candidate components of this oscillator, such as BLMP-1, GRH-1, NHR-25, MYRF-1 or BED-1, could eventually drive animals to molt in the absence of NHR-23 (Hauser et al., 2022 preprint; Meeuse et al., 2023; Stec et al., 2021; Stojanovski et al., 2022).

### *nhr-23* is necessary in seam and hypodermal cells to promote molting

Seam cell-enriched *nhr-23* RNAi caused severe developmental delay and molting defects (Fig. 8). Hypodermal-enriched *nhr-23* knockdown produced less penetrant phenotypes (Fig. 8). The most severe defects in ROL-6 and NOAH-1 localization occur above the seam cells (Figs 5 and 6). Determining how NHR-23 regulates BLI-1 expression and formation of struts will provide insight into aECM assembly. *nhr-23* is expressed in other epidermal cells that produce the pre-cuticle and cuticle, such as vulval precursor cells, rectal cells and excretory duct cells. NHR-23 depletion did not produce obvious defects in localization of the pre-cuticle component NOAH-1 in these cells (Fig. 5). We did not see phenotypes such as excretory duct or pore lumen dilation, which would be indicative of excretory system defects (Gill et al., 2016). We observed vulval morphology defects after NHR-23 depletion, but it is not clear whether that is due to NHR-23 regulation of this specialized aECM or whether it is the consequence of developmental delay and failure to molt. Tissue-specific RNAi or protein depletion will be required to determine whether NHR-23 activity is necessary in these epithelial cells or whether it predominantly functions in seam and hypodermal cells. Our data indicate the importance of tissue-specific RNAi strain validation. *SCMp* drove reporter expression robustly in seam cells (Fig. S8). However, when we made a tissue-specific RNAi strain using *SCMp*, we observed depletion in seam, hypodermal and intestinal cells (Fig. S9). RNAi has an inherent amplification of dsRNA triggers by RNA-dependent RNA polymerases (Zhang and Ruvkun, 2012). Weak promoter activity in undesired tissues could reconstitute RNAi in these tissues. A systematic analysis of tissue-specific RNAi strains is an important future direction for interpreting studies using these strains.

### Future perspectives

This work highlights the power of timed protein depletion for dissecting the role of oscillating developmental regulators in development. As transcription factors coordinate the expression of batteries of genes in a given biological process, future work will reveal how NHR-23 coordinates apical ECM remodeling, apolysis and ecdysis.

## MATERIALS AND METHODS

### *C. elegans* strains and culture

*C. elegans* strains (see Table 1) were cultured as originally described (Brenner, 1974), except worms were grown on MYOB instead of NGM. MYOB was made as previously described (Church et al., 1995). Animals were cultured at 20°C for all assays, unless otherwise indicated. For general strain propagation, animals were grown at 15°C according to standard protocols. Brood sizes were controlled by picking L4 larvae to individual wells of a six-well plate seeded with OP50 and incubating the plate at 20°C. Animals were transferred to new plates daily over 4 days. Two days post-transfer, the number of hatched progeny and unfertilized eggs were scored.

**Table 1.**
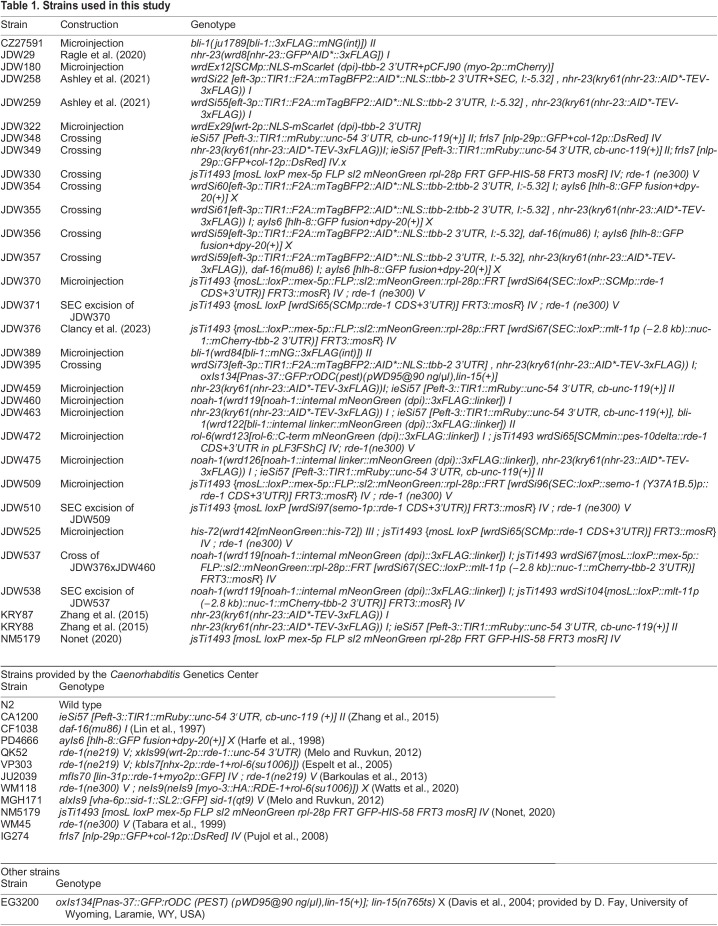
Strains used in this study

Strains provided by the *Caenorhabditis* Genetics Center	
Strain	Genotype
N2	Wild type
CA1200	*ieSi57 [Peft-3::TIR1::mRuby::unc-54 3′UTR, cb-unc-119 (+)] II* (Zhang et al., 2015)
CF1038	*daf-16(mu86) I* (Lin et al., 1997)
PD4666	*ayIs6 [hlh-8::GFP fusion+dpy-20(+)] X* (Harfe et al., 1998)
QK52	*rde-1(ne219) V; xkIs99(wrt-2p::rde-1::unc-54 3'UTR)* (Melo and Ruvkun, 2012)
VP303	*rde-1(ne219) V; kbIs7[nhx-2p::rde-1+rol-6(su1006)])* (Espelt et al., 2005)
JU2039	*mfIs70 [lin-31p::rde-1+myo2p::GFP] IV ; rde-1(ne219) V* (Barkoulas et al., 2013)
WM118	*rde-1(ne300) V ; neIs9(neIs9 [myo-3::HA::RDE-1+rol-6(su1006)]) X* (Watts et al., 2020)
MGH171	*alxIs9 [vha-6p::sid-1::SL2::GFP] sid-1(qt9) V* (Melo and Ruvkun, 2012)
NM5179	*jsTi1493 [mosL loxP mex-5p FLP sl2 mNeonGreen rpl-28p FRT GFP-HIS-58 FRT3 mosR] IV* (Nonet, 2020)
WM45	*rde-1(ne300) V* (Tabara et al., 1999)
IG274	*frIs7 [nlp-29p::GFP+col-12p::DsRed] IV* (Pujol et al., 2008)

Other strains	
Strain	Genotype
EG3200	*oxIs134[Pnas-37::GFP:rODC (PEST) (pWD95@90 ng/µl),lin-15(+)]; lin-15(n765ts)* X (Davis et al., 2004; provided by D. Fay, University of Wyoming, Laramie, WY, USA)

### Genome editing and transgenesis

*mNeonGreen::3xFLAG* knock-ins into *bli-1, noah-1* and *rol-6,* and *mNeonGreen* knock-ins into *his-72* were generated by injection of Cas9 ribonucleoprotein complexes [700 ng/µl Alt-R S.p. Cas9 Nuclease V3 (IDT), 115 ng/µl crRNA and 250 ng/µl IDT tracrRNA] and a dsDNA repair template (25-50 ng/μl) created by PCR amplification of a plasmid template (Paix et al., 2014, 2015). The *bli-1* and *noah-1* knock-ins were internal and the *mNG::3xFLAG* cassette was flanked by flexible glycine- and serine-rich linker sequences. Generation of the *BLI-1::3xFLAG::mNG(int)* used in Fig. 6C will be described elsewhere (J. Adams and A.D.C., unpublished). The C-terminal *rol-6::mNG::3xFLAG* fusion encoded a flexible linker between the end of *rol-6* and the start of mNG. The *noah-1::mNG::3xFLAG(int)* knock-in used a pJW2332 repair template, which will be described elsewhere (J.M.R. and J.D.W., unpublished). The PCR products were melted to boost editing efficiency, as previously described (Ghanta and Mello, 2020). crRNAs used are provided in Table S6. Oligonucleotides used for repair template generation from template pJW2172 (Ashley et al., 2021) and for genotyping are provided in Table S7. Plasmids used are provided in Table S8. To generate JDW371 and JDW510 (seam cell and hypodermal-specific tissue-specific RNAi), we crossed a *jsTi1493* landing pad for recombination-mediated cassette exchange (RMCE) (Nonet, 2020) into an *rde-1(ne300*) null mutant, generating JDW330. We used Gibson cloning to introduce a promoterless *rde-1* genomic coding sequence+3′UTR fragment into the RMCE integration vector pLF3FShC (Nonet, 2020), creating pJW2247. This vector can be linearized with AvrII+BsiWI double-digestion and promoters can be introduced by Gibson cloning (Gibson et al., 2009). We generated a *SCMp* (seam cell-specific) and *semo-1p* (hypodermis specific) by Gibson cloning of promoters into linearized pJW2247, constructing pJW2236 and pJW2264, respectively. These vectors were integrated into *jsTi1493* landing pad in JDW330 using RMCE, as described previously (Nonet, 2020). *wrt-2p* and *SCMp* promoter reporters were constructed in pJW1836 (*NLS::mScarlet::tbb-2 3′ UTR*) (Ashley et al., 2021) by Gibson cloning. Plasmids were injected into N2 animals at 50 ng/µl with a pCFJ90 co-injection marker at 10 ng/µl for the *SCMp* promoter reporter and a pRF4 co-injection marker at 50 ng/µl for the *wrt-2* promoter reporter (Frøkjaer-Jensen et al., 2008; Mello et al., 1991). Transgenic lines propagating extrachromosomal arrays were generated as previously described (Mello et al., 1991). Genomic and knock-in sequences are provided as GenBank-compatible .ape files on figshare.com (File S1, https://doi.org/10.6084/m9.figshare.22270525.v1). Sequence files for plasmids are provided as Genbank-compatible .ape files on figshare.com (File S2, https://doi.org/10.6084/m9.figshare.22270522.v1).

### Auxin treatment

Control and auxin media/plates were made as described by Ragle et al. (2020). Control media consisted of MYOB agar+0.25% ethanol. Auxin media was made by dissolving indole 3-acetic acid (IAA; Alfa Aesar, AAA1055622) in 100% ethanol to 1.6 M and then mixing it into melted MYOB agar at 55°C to a final concentration of 4 mM before pouring plates. Control plates contained 0.25% ethanol. Temperature of the media was monitored with a Lasergrip 1080 infrared thermometer gun (Etekcity). Plates were seeded with *E. coli* OP50 and incubated overnight at room temperature. Plates were stored for up to 1 month at 4°C before use. For most auxin treatment experiments, animals were synchronized by alkaline bleaching (www.protocols.io/view/ward-lab-alkaline-bleaching-protocol-cbq9smz61). The collected eggs were incubated in M9 buffer supplemented with 5 mg/ml cholesterol at 20°C for 24 h and arrested L1 larvae were released onto the indicated type of MYOB plate. For the experiment in Table S1, a timed egg lay was performed. Twenty adult hermaphrodites animals of the indicated genotype were picked onto control (0.25% ethanol) or auxin (4 mM IAA) plates and allowed to lay eggs for 2 h. The adult animals were removed and plates were incubated for 48 h at 25°C.

### Microscopy

Synchronized animals were collected from MYOB, control or auxin plates by either picking or washing off plates. For washing, 1000 µl of M9 and 0.05% gelatin was added to the plate or well, agitated to suspend animals in M9 and gelatin, and then transferred to a 1.5 ml tube. Animals were spun at 700 ***g*** for 1 min. The medium was then aspirated off and animals were resuspended in 500 µl M9 and 0.05% gelatin with 5 mM levamisole. 12 µl of animals in M9 and 0.05% levamisole solution were placed on slides with a 2% agarose pad and secured with a coverslip. For picking, animals were transferred to a 10 µl drop of M9+5 mM levamisole on a 2% agarose pad on a slide and secured with a coverslip. Images were acquired using a Plan-Apochromat 40×/1.3 Oil DIC lens, a Plan-Apochromat 63×/1.4 Oil DIC lens or a Plan-Apochromat 100×/1.4 OIL DIC lens on an AxioImager M2 microscope (Carl Zeiss Microscopy) equipped with a Colibri 7 LED light source and an Axiocam 506 mono camera. Acquired images were processed using Fiji software (version: 2.0.0- rc-69/1.52p) (Schindelin et al., 2012). For direct comparisons within a figure, we set the exposure conditions to avoid pixel saturation of the brightest sample and kept equivalent exposure for imaging of the other samples. Colocalization using a Manders' co-efficient (Manders et al., 1992, 1993) was performed as described previously (Clancy et al., 2023).

### Phenotypic analysis

For the phenotypic analysis in Fig. 2, synchronized CA1200 or KRY88 larvae were released onto MYOB plates and then shifted onto control or auxin plates every 2 h up to 16 h. Animals were collected as described in the Microscopy section and imaged by DIC microscopy to score for morphology and shedding of the L1 cuticles. For the M cell lineage experiments in Fig. S3, synchronized animals of the indicated genotypes were released onto MYOB plates and shifted onto control or auxin plates at 3 h (early shift) or 9 h (late shift) post-release and then imaged at 24 h post-release, as described in the Microscopy section. M cells were counted and recorded. For the analyses in Fig. 2E, synchronized *nhr-23::AID, TIR1* larvae were released on auxin plates and scored for viability and molting defects 72 h later. For the L3 shift experiments (Fig. 2F,G) synchronized animals of the indicated genotype were grown on MYOB. At 25 h post-release, they were shifted onto six-well control or auxin MYOB plates seeded with OP50. Animals were collected 23 or 72 h later, as described in the Microscopy section and imaged by DIC microscopy using a 63× lens to stage animals according to vulval morphology (Mok et al., 2015). For the reporter timecourse, synchronized *nhr-23::AID, TIR1, nas-37p::GFP::PEST* animals were released on control or auxin plates. They were then scored for GFP expression using a PlanApo 5.0×/0.5 objective on a M165 FC stereomicroscope (Leica) equipped with an X-cite FIRE LED lightsource (Excelitas) and long-pass GFP filter set (Leica, 10447407). We scored GFP expression in the head and hypodermis and did not score rectal GFP expression, as expression perdured in this tissue after head and hypodermal GFP expression ceased.

### Barrier assays

Hoechst 33258 staining was performed as described previously (Moribe et al., 2004), except that we used 10 µg/ml of Hoechst 33258 as in Ward et al. (2014). Two biological replicates were performed examining 50 animals per experiment. The number of animals with either head or hypodermal nuclei staining with Hoechst 33258 were scored under a 10× DIC objective. Representative images were then taken with equivalent exposures using a 63× Oil DIC lens, as described in the imaging section. Hypo-osmotic stress sensitivity assays were performed on L4 stage larvae as described previously (Ward et al., 2014), except we used 20 µl of deionized H_2_0. Each strain was assayed in triplicate.

### Western blots

Animals were synchronized by alkaline bleaching, as described in the Auxin treatment section. For the blots in Figs 5B and 6B, 30 animals of the indicated genotype and stage were picked into 30 µl of M9+0.05% gelatin and 10 µl of 4×Laemmli sample buffer was added and then samples were heated at 95°C for 5 min and stored at −80°C until they were resolved by SDS-PAGE. For the blots in Fig. 6C, synchronized animals of the indicated genotype were collected at the L4 stage. Extracts of soluble cuticle proteins were prepared as described previously (Cox et al., 1981) with minor modifications. Briefly, worms were collected using M9J buffer in a 15 ml Falcon tube and washed three times with M9J buffer. Worm pellets were resuspended in 5 ml of sonication buffer and incubated on ice for 10 min and sonicated (Misonix XL-2000 Series Ultrasonic Liquid Processor, 10×10 s pulse with 10 s break) with 30 μl of 0.1 M PMSF. After sonication, samples were centrifuged at 6010 ***g*** for 2 min at 4°C and supernatants stored as fraction 1 (F1; intracellular proteins) at −20°C for the purpose of using it as loading control. Pellets were washed three times with sonication buffer and the resultant pellets were resuspended in 100 μl sonication buffer, boiled at 95°C for 2 min with 1 ml of ST buffer and incubated overnight with rotation at room temperature. After incubation, samples were centrifuged at 6010 ***g*** for 3 min and supernatants were stored as fraction 2 (F2) at −20°C. Subsequently, cuticle pellets were washed three times with 0.5% Triton X-100 and boiled at 95°C for 2 min with 300 μl of ST buffer with 5% β-mercaptoethanol and incubated with rotation at room temperature for 16 h. Finally, the samples were centrifuged at 6010 ***g*** for 3 min and supernatants collected and stored as fraction 3 (F3; soluble cuticle proteins). F3 was precipitated with methanol/chloroform and resuspended in 100 μl of rehydration buffer.

For the remaining blots, animals of the indicated genotype were washed out of wells on a six-well plate at the indicated timepoints with M9+0.05% gelatin (VWR, 97062-620), transferred to a 1.5 ml tube and washed twice more with M9+0.05% gelatin, as previously described (Vo et al., 2021). Animals were pelleted, transferred into a 100 µl volume to a new 1.5 ml tube, and flash frozen in liquid nitrogen, then stored at −80°C. Before SDS-PAGE, samples were freeze-cracked twice in liquid nitrogen or on dry ice, Laemmli sample buffer was added to 1× and samples were heated to 95°C for 5 min. For the western blots in Fig. 1C,D, 6000 KRY88 animals per well were transferred onto a six-well MYOB plate seeded with OP50. For the western blot in Fig. 2B, 6000 synchronized KRY88 animals were plated onto control or auxin plates. Control animals were harvested at peak NHR-23 expression (12 h of growth on control plates) and auxin-treated animals were shifted onto auxin at this time; samples were taken at the indicated time points, as described above. For the western blots in Fig. S7, 500 animals of the indicated genotype were transferred into wells of a six-well control or auxin plate seeded with OP50. Animals were incubated for 24 h at 20°C and collected as described above.

For the blots in Fig. 1C,D, 10 µl of lysate and 7.5 µl of a 1:1 mix of Amersham ECL Rainbow Molecular Weight Markers (95040-114) and Precision Plus Protein Unstained Standards (1610363) was resolved by SDS-PAGE using precast 4-20% MiniProtean TGX Stain Free Gels (Bio-Rad). The blot in Fig. S7, the lysates had much more variable levels of total protein, particularly the NHR-23-depleted samples that produced an early larval arrest. Therefore, we quantified the signal of the most intense band by Stain Free imaging (Posch et al., 2013) using a Bio-Rad ChemiDoc imaging system, and ran a new gel with normalized loading. The lysates in Figs 2A, 5B, 6B and S7 were resolved as described above except we used a Spectra Multicolor Broad Range Protein Ladder (ThermoFisher Scientific, 26623). Proteins were transferred to a polyvinylidene difluoride membrane by semi-dry transfer with a TransBlot Turbo (Bio-Rad). In Figs 1C and 7C, total protein, pre- and post-transfer, was monitored using the stain-free fluorophore as described (Posch et al., 2013; Ward, 2015a). Membranes were washed in TBST and blocked in TBST+5% milk (TBST-M; Nestle Carnation Instant Nonfat Dry Milk) for 1 h at room temperature. Blots were rocked in primary antibodies in TBST-M overnight at 4°C and then washed four times for 5 min each with TBST. For primary antibodies conjugated with horseradish peroxidase (HRP), the blots were developed after the last TBST wash. Otherwise, blots were incubated with HRP-conjugated secondary antibodies in TBST-M for 1 h at room temperature followed by four 5 min TBST washes and then developed as described below. The primary antibody used for Figs 1C,D, 2A, 5B and 6B was horseradish peroxidase (HRP)-conjugated anti-FLAG M2 (Sigma-Aldrich, A8592-5x1MG, Lot #SLCB9703) at a 1:2000 dilution. Precision Protein StrepTactin-HRP Conjugate (Bio-Rad, 1610381, Lot #64426657) was included with the primary antibody at a 1:10,000 dilution to visualize the protein size standard during blot imaging for Figs 1C,D. For the blot in Figs 2A, 5B and 6B, we used mouse anti-alpha-tubulin 12G10 [Developmental Studies Hybridoma Bank, the ‘-c’ concentrated supernatant at 1:2000 (Fig. 2A, Fig. S7) or 1:4000 (Figs 5B, 6B)]. The secondary antibodies were Digital anti-mouse (Kindle Biosciences LLC, R1005) diluted 1:10,000 (Fig. 2A, Fig. S7) or 1:20,000 (Fig. S6B,C). Blots were incubated for 5 min with 1 ml of Supersignal West Femto Maximum Sensitivity Substrate (ThermoFisher Scientific, 34095) and imaged using the ‘chemi high-resolution’ setting on a Bio-Rad ChemiDoc MP System.

For the blots in Fig. 6C, 20 μl was loaded on to NuPAGE 4 to 12% protein gels with 7 μl of 4× NuPAGE LDS sample buffer as described by the manufacturer (Invitrogen, NP0322BOX) and run for 2 h at 100 V, transferred to 0.45 μm nitrocellulose membrane (Bio-Rad) at 4°C for 90 min using Bio-Rad WET transfer system. After transfer, membranes were blocked in Intercept blocking buffer (LI-COR: 927-60001) for 1 h. Later, the membrane was incubated overnight in anti-mNeonGreen primary antibody (ChromoTek, 32F6) at 1:5000 dilution at 4°C on rocker. Membranes were washed three times with TBST and incubated with secondary antibody (Sigma 12-349) at 1:100,000 dilution at room temperature on rocker for 1 h and washed three times with TBST. Blots were visualized with Supersignal West Femto detection kit (Thermo Fisher) using a LI-COR Odyssey imager. Similarly, to test the loading control, 10 μl of F1 samples were loaded and, after transfer to nitrocellulose membrane, probed with monoclonal mouse anti-actin clone C4 (ThermoFisher Scientific: ICN691001).

### Bioinformatics

Fig. 3A was generated in R (R Core Team). Phase and amplitude in Table S2 were converted to *x* and *y* coordinates for each gene by calculating *x*=amplitude×cos(phase) and *y*=amplitude×sin(phase). For Figs 3D and S5, L3 NHR-23 ChIP-Seq data was downloaded from http://www.modencode.org (accession ID modEncode_3837). Wig files were converted to bigwig format and loaded into the Bioconductor package SeqPlots (Huber et al., 2015; Stempor and Ahringer, 2016). A bed file with the start and end coordinates of the CDS of all genes was generated (Wormbase WS220) and loaded into SeqPlots. SeqPlots aligned all genes by the start and end position, and scaled the coding sequence of each gene to 2 kb. The average signal over all aligned genes was calculated in 25 bp windows from 1 kb upstream of the start site to 1 kb downstream of the end site. Peak calls for NHR-23 in L3 (Fig. 4C) were obtained from GEO (accession numbers: GSM1183659, GSM1183660, GSM1183661 and GSM118366). Only peak calls validated by both replicates were considered. Peaks were assigned to the following genome features: gene body (WormBase WS220); 1 kb upstream of the start site of coding genes; between 1 kb and 3 kb upstream of the gene start site; and between 3 kb and 5 kb upstream of the gene start site. The same intervals were chosen downstream of the gene end sites. An NHR-23 peak was assigned to a feature if it overlapped with it by at least 100 bp. The peak analysis data are in Table S5. The total number of peaks in all bins was tallied and is presented in Table S2 and in Fig. 3D. For Figs 3D and 4E, the phase for the oscillating genes in Table S1 was converted to time during the molting cycle by assuming a 9 h larval stage, which makes each hour=40°. We used the phasing from Meeuse et al. (2023) with lethargus starting at 45° and ecdysis ending at 135°. The phase in hours was then plotted along the *x*-axis and the amplitude of the oscillating genes was plotted on the *y*-axis. The gene annotation was based on the Concise Description and Automated Description attributes downloaded from Wormbase.

### RNAi

RNAi feeding plates were made by melting a solidified bottle of MYOB and adding carbenicillin (25 μg/ml) and IPTG (8 mM) once cooled to 55°C. dsRNA-expressing *E. coli* bacteria were streaked on LB plates with ampicillin (100 µg/ml) and grown overnight at 37°C. A single colony was picked into 25 ml LB with 100 µg/ml ampicillin and 12.5 µg/ml tetracycline and shaken overnight at 37°C at 220 RPM. The next day, the liquid culture was pelleted and resuspended in 1.25 ml LB with 100 µg/ml ampicillin, resulting in a 20× concentration from the original overnight liquid culture. 90 µl of the liquid culture was spread per small RNAi plate and allowed to dry. The plates were incubated at room temperature in the dark for 3 days. Ten adult worms from each strain were allowed to lay eggs on plates spread with *E. coli* bacteria containing either the control plasmid L4440 or the *nhr-23* RNAi knockdown plasmid for 1-2 h. The adults were removed and the eggs allowed to develop at 20°C for 2 or 3 days, depending on the experiment. For tissue-specific RNAi experiments, we used RNAi defective *rde-1* mutant animals carrying *rde-1* transgenes to rescue RNAi in specific tissues (Qadota et al., 2007) The one exception was strain MGH171, which used an intestinal-specific rescue of *sid-1* in an RNAi defective *sid-1* mutant (Melo and Ruvkun, 2012).

### Statistical analysis

Statistical tests and numbers of animals analyzed are detailed in figure legends.

## Supplementary Material

10.1242/develop.201085_sup1 Supplementary information

## References

[DEV201085C1] Agbulut, O., Coirault, C., Niederländer, N., Huet, A., Vicart, P., Hagège, A., Puceat, M. and Menasché, P. (2006). GFP expression in muscle cells impairs actin-myosin interactions: implications for cell therapy. *Nat. Methods* 3, 331. 10.1038/nmeth0506-331 16628201

[DEV201085C2] Antebi, A. (2015). Nuclear receptor signal transduction in *C. elegans*. *WormBook*, 1-49. 10.1895/wormbook.1.64.2 PMC5402207 26069085

[DEV201085C3] Ashley, G. E., Duong, T., Levenson, M. T., Martinez, M. A. Q., Johnson, L. C., Hibshman, J. D., Saeger, H. N., Palmisano, N. J., Doonan, R., Martinez-Mendez, R. et al. (2021). An expanded auxin-inducible degron toolkit for *Caenorhabditis elegans*. *Genetics* 217, iyab006. 10.1093/genetics/iyab006 33677541 PMC8045686

[DEV201085C4] Aspöck, G., Kagoshima, H., Niklaus, G. and Bürglin, T. R. (1999). *Caenorhabditis elegans* has scores of hedgehog related genes: sequence and expression analysis. *Genome Res.* 9, 909-923. 10.1101/gr.9.10.909 10523520

[DEV201085C5] Baens, M., Noels, H., Broeckx, V., Hagens, S., Fevery, S., Billiau, A. D., Vankelecom, H. and Marynen, P. (2006). The dark side of EGFP: defective polyubiquitination. *PLoS ONE* 1, e54. 10.1371/journal.pone.0000054 17183684 PMC1762387

[DEV201085C6] Barkoulas, M., van Zon, J. S., Milloz, J., van Oudenaarden, A. and Félix, M.-A. (2013). Robustness and Epistasis in the *C. elegans* vulval signaling network revealed by pathway dosage modulation. *Dev. Cell* 24, 64-75. 10.1016/j.devcel.2012.12.001 23328399

[DEV201085C8] Blaxter, M. L. (1993). Cuticle surface proteins of wild type and mutant *Caenorhabditis elegans*. *J. Biol. Chem.* 268, 6600-6609. 10.1016/S0021-9258(18)53293-2 8503957

[DEV201085C9] Blaxter, M. L., Page, A. P., Rudin, W. and Maizels, R. M. (1992). Nematode surface coats: actively evading immunity. *Parasitol. Today* 8, 243-247. 10.1016/0169-4758(92)90126-M 15463630

[DEV201085C10] Brenner, S. (1974). The genetics of *Caenorhabditis elegans*. *Genetics* 77, 71-94. 10.1093/genetics/77.1.71 4366476 PMC1213120

[DEV201085C11] Campbell, L. A., Faivre, E. J., Show, M. D., Ingraham, J. G., Flinders, J., Gross, J. D. and Ingraham, H. A. (2008). Decreased recognition of SUMO-sensitive target genes following modification of SF-1 (NR5A1). *Mol. Cell. Biol.* 28, 7476-7486. 10.1128/MCB.00103-08 18838537 PMC2593425

[DEV201085C12] Church, D. L., Guan, K. L. and Lambie, E. J. (1995). Three genes of the MAP kinase cascade, *mek-*2, *mpk-*1*/sur-*1 and *let-*60 *ras*, are required for meiotic cell cycle progression in *Caenorhabditis elegans*. *Development* 121, 2525-2535. 10.1242/dev.121.8.2525 7671816

[DEV201085C13] Clancy, J. C., Vo, A. A., Myles, K. M., Levenson, M. T., Ragle, J. M. and Ward, J. D. (2023). Experimental considerations for study of *C. elegans* lysosomal proteins. *G3* 13, jkad032. 10.1093/g3journal/jkad032 36748711 PMC10085801

[DEV201085C14] Clark, D. V., Suleman, D. S., Beckenbach, K. A., Gilchrist, E. J. and Baillie, D. L. (1995). Molecular cloning and characterization of the *dpy-*20 gene of *Caenorhabditis elegans*. *Mol. Gen. Genet.* 247, 367-378. 10.1007/BF00293205 7770042

[DEV201085C15] Cohen, J. D. and Sundaram, M. V. (2020). *C. elegans* apical extracellular matrices shape epithelia. *J. Dev. Biol.* 8, 23. 10.3390/jdb8040023 33036165 PMC7712855

[DEV201085C16] Cohen, J. D., Sparacio, A. P., Belfi, A. C., Forman-Rubinsky, R., Hall, D. H., Maul-Newby, H., Frand, A. R. and Sundaram, M. V. (2020). A multi-layered and dynamic apical extracellular matrix shapes the vulva lumen in *Caenorhabditis elegans*. *eLife* 9, e57874. 10.7554/eLife.57874 32975517 PMC7544507

[DEV201085C17] Cox, G. N., Kusch, M. and Edgar, R. S. (1981). Cuticle of *Caenorhabditis elegans*: its isolation and partial characterization. *J. Cell Biol.* 90, 7-17. 10.1083/jcb.90.1.7 7251677 PMC2111847

[DEV201085C18] Davis, M. W., Birnie, A. J., Chan, A. C., Page, A. P. and Jorgensen, E. M. (2004). A conserved metalloprotease mediates ecdysis in *Caenorhabditis elegans*. *Development* 131, 6001-6008. 10.1242/dev.01454 15539494

[DEV201085C19] de Sousa Abreu, R., Penalva, L. O., Marcotte, E. M. and Vogel, C. (2009). Global signatures of protein and mRNA expression levels. *Mol. Biosyst.* 5, 1512-1526. 10.1039/b908315d 20023718 PMC4089977

[DEV201085C20] Dodd, W., Tang, L., Lone, J.-C., Wimberly, K., Wu, C.-W., Consalvo, C., Wright, J. E., Pujol, N. and Choe, K. P. (2018). A damage sensor associated with the cuticle coordinates three core environmental stress responses in *Caenorhabditis elegans*. *Genetics* 208, 1467-1482. 10.1534/genetics.118.300827 29487136 PMC5887142

[DEV201085C21] Espelt, M. V., Estevez, A. Y., Yin, X. and Strange, K. (2005). Oscillatory Ca2+ signaling in the isolated *Caenorhabditis elegans* intestine: role of the inositol-1,4,5-trisphosphate receptor and phospholipases C beta and gamma. *J. Gen. Physiol.* 126, 379-392. 10.1085/jgp.200509355 16186564 PMC2266627

[DEV201085C22] Fernandez, A. P., Gibbons, J. and Okkema, P. G. (2004). *C. elegans peb-1* mutants exhibit pleiotropic defects in molting, feeding, and morphology. *Dev. Biol.* 276, 352-366. 10.1016/j.ydbio.2004.08.040 15581870

[DEV201085C23] Forman-Rubinsky, R., Cohen, J. D. and Sundaram, M. V. (2017). Lipocalins are required for apical extracellular matrix organization and remodeling in *Caenorhabditis elegans*. *Genetics* 207, 625-642. 10.1534/genetics.117.300207 28842397 PMC5629328

[DEV201085C24] Frand, A. R., Russel, S. and Ruvkun, G. (2005). Functional Genomic Analysis of *C. elegans* Molting. *PLoS Biol.* 3, e312. 10.1371/journal.pbio.0030312 16122351 PMC1233573

[DEV201085C25] Frøkjaer-Jensen, C., Davis, M. W., Hopkins, C. E., Newman, B. J., Thummel, J. M., Olesen, S.-P., Grunnet, M. and Jorgensen, E. M. (2008). Single-copy insertion of transgenes in *Caenorhabditis elegans*. *Nat. Genet.* 40, 1375-1383. 10.1038/ng.248 18953339 PMC2749959

[DEV201085C26] Gerstein, M. B., Lu, Z. J., Van Nostrand, E. L., Cheng, C., Arshinoff, B. I., Liu, T., Yip, K. Y., Robilotto, R., Rechtsteiner, A., Ikegami, K. et al. (2010). Integrative analysis of the *Caenorhabditis elegans* genome by the modENCODE Project. *Science* 330, 1775-1787. 10.1126/science.1196914 21177976 PMC3142569

[DEV201085C27] Ghanta, K. S. and Mello, C. C. (2020). Melting dsDNA donor molecules greatly improves precision genome editing in *Caenorhabditis elegans*. *Genetics* 216, 643-650. 10.1534/genetics.120.303564 32963112 PMC7648581

[DEV201085C28] Ghedin, E., Wang, S., Spiro, D., Caler, E., Zhao, Q., Crabtree, J., Allen, J. E., Delcher, A. L., Guiliano, D. B., Miranda-Saavedra, D. et al. (2007). Draft genome of the filarial nematode parasite *Brugia malayi*. *Science* 317, 1756-1760. 10.1126/science.1145406 17885136 PMC2613796

[DEV201085C29] Gibson, D. G., Young, L., Chuang, R.-Y., Venter, J. C., Hutchison, C. A. and Smith, H. O. (2009). Enzymatic assembly of DNA molecules up to several hundred kilobases. *Nat. Methods* 6, 343-345. 10.1038/nmeth.1318 19363495

[DEV201085C30] Gill, H. K., Cohen, J. D., Ayala-Figueroa, J., Forman-Rubinsky, R., Poggioli, C., Bickard, K., Parry, J. M., Pu, P., Hall, D. H. and Sundaram, M. V. (2016). Integrity of narrow epithelial tubes in the C. elegans excretory system requires a transient luminal matrix. *PLoS Genet.* 12, e1006205. 10.1371/journal.pgen.1006205 27482894 PMC4970718

[DEV201085C31] Gissendanner, C. R., Crossgrove, K., Kraus, K. A., Maina, C. V. and Sluder, A. E. (2004). Expression and function of conserved nuclear receptor genes in *Caenorhabditis elegans*. *Dev. Biol.* 266, 399-416. 10.1016/j.ydbio.2003.10.014 14738886

[DEV201085C32] Guo, P., Hu, T., Zhang, J., Jiang, S. and Wang, X. (2010). Sequential action of *Caenorhabditis elegans* Rab GTPases regulates phagolysosome formation during apoptotic cell degradation. *Proc. Natl. Acad. Sci. USA* 107, 18016-18021. 10.1073/pnas.1008946107 20921409 PMC2964220

[DEV201085C33] Gupta, S. K. (2021). Human Zona Pellucida glycoproteins: binding characteristics with human spermatozoa and induction of acrosome reaction. *Front. Cell Dev. Biol.* 9, 619868. 10.3389/fcell.2021.619868 33681199 PMC7928326

[DEV201085C34] Harfe, B. D., Gomes, A. V., Kenyon, C., Liu, J., Krause, M. and Fire, A. (1998). Analysis of a *Caenorhabditis elegans* Twist homolog identifies conserved and divergent aspects of mesodermal patterning. *Genes Dev.* 12, 2623-2635. 10.1101/gad.12.16.2623 9716413 PMC317087

[DEV201085C35] Hauser, Y. P., Meeuse, M. W. M., Gaidatzis, D. and Großhans, H. (2022). The BLMP-1 transcription factor promotes oscillatory gene expression to achieve timely molting. *bioRxiv* 2021.07.05.450828.

[DEV201085C36] Hendriks, G.-J., Gaidatzis, D., Aeschimann, F. and Großhans, H. (2014). Extensive oscillatory gene expression during *C. elegans* larval development. *Mol. Cell* 53, 380-392. 10.1016/j.molcel.2013.12.013 24440504

[DEV201085C37] Huber, W., Carey, V. J., Gentleman, R., Anders, S., Carlson, M., Carvalho, B. S., Bravo, H. C., Davis, S., Gatto, L., Girke, T. et al. (2015). Orchestrating high-throughput genomic analysis with Bioconductor. *Nat. Methods* 12, 115-121. 10.1038/nmeth.3252 25633503 PMC4509590

[DEV201085C38] Jetten, A. M. (2009). Retinoid-related orphan receptors (RORs): critical roles in development, immunity, circadian rhythm, and cellular metabolism. *Nucl. Recept. Signal.* 7, e003. 10.1621/nrs.07003 19381306 PMC2670432

[DEV201085C39] Johnstone, I. L. and Barry, J. D. (1996). Temporal reiteration of a precise gene expression pattern during nematode development. *EMBO J.* 15, 3633-3639. 10.1002/j.1460-2075.1996.tb00732.x 8670866 PMC451985

[DEV201085C40] Kage-Nakadai, E., Kobuna, H., Kimura, M., Gengyo-Ando, K., Inoue, T., Arai, H. and Mitani, S. (2010). Two very long chain fatty acid acyl-CoA synthetase genes, *acs-*20 and *acs-*22, have roles in the cuticle surface barrier in *Caenorhabditis elegans*. *PLoS ONE* 5, e8857. 10.1371/journal.pone.0008857 20111596 PMC2810326

[DEV201085C41] Kaletsky, R., Yao, V., Williams, A., Runnels, A. M., Tadych, A., Zhou, S., Troyanskaya, O. G. and Murphy, C. T. (2018). Transcriptome analysis of adult *Caenorhabditis elegans* cells reveals tissue-specific gene and isoform expression. *PLoS Genet.* 14, e1007559. 10.1371/journal.pgen.1007559 30096138 PMC6105014

[DEV201085C42] Kasuga, H., Fukuyama, M., Kitazawa, A., Kontani, K. and Katada, T. (2013). The microRNA miR-235 couples blast-cell quiescence to the nutritional state. *Nature* 497, 503-506. 10.1038/nature12117 23644454

[DEV201085C43] Katz, S. S., Barker, T. J., Maul-Newby, H. M., Sparacio, A. P., Nguyen, K. C. Q., Maybrun, C. L., Belfi, A., Cohen, J. D., Hall, D. H., Sundaram, M. V. et al. (2022). A transient apical extracellular matrix relays cytoskeletal patterns to shape permanent acellular ridges on the surface of adult *C. elegans*. *PLoS Genet.* 18, e1010348. 10.1371/journal.pgen.1010348 35960773 PMC9401183

[DEV201085C44] Kelley, M., Yochem, J., Krieg, M., Calixto, A., Heiman, M. G., Kuzmanov, A., Meli, V., Chalfie, M., Goodman, M. B., Shaham, S. et al. (2015). FBN-1, a fibrillin-related protein, is required for resistance of the epidermis to mechanical deformation during *C. elegans* embryogenesis. *eLife* 4, 1629. 10.7554/eLife.06565 PMC4395870 25798732

[DEV201085C45] Kiefer, S. M. L. and Saling, P. (2002). Proteolytic processing of human zona pellucida proteins. *Biol. Reprod.* 66, 407-414. 10.1095/biolreprod66.2.407 11804956

[DEV201085C46] Kim, D., Grün, D. and van Oudenaarden, A. (2013). Dampening of expression oscillations by synchronous regulation of a microRNA and its target. *Nat. Genet.* 45, 1337-1344. 10.1038/ng.2763 24036951 PMC3812263

[DEV201085C47] King-Jones, K. and Thummel, C. S. (2005). Nuclear receptors-a perspective from *Drosophila*. *Nat. Rev. Genet.* 6, 311-323. 10.1038/nrg1581 15803199

[DEV201085C48] Kinney, B., Sahu, S., Stec, N., Hills-Muckey, K., Adams, D. W., Wang, J., Jaremko, M., Joshua-Tor, L., Keil, W. and Hammell, C. M. (2023). A circadian-like gene network regulates heterochronic miRNA transcription in *C. elegans*. *bioRxiv* 2022.09.26.509508. 10.1016/j.devcel.2023.08.006 PMC10840721 37643611

[DEV201085C49] Köhnlein, K., Urban, N., Guerrero-Gómez, D., Steinbrenner, H., Urbánek, P., Priebs, J., Koch, P., Kaether, C., Miranda-Vizuete, A. and Klotz, L.-O. (2020). A *Caenorhabditis elegans* ortholog of human selenium-binding protein 1 is a pro-aging factor protecting against selenite toxicity. *Redox Biol.* 28, 101323. 10.1016/j.redox.2019.101323 31557719 PMC6812014

[DEV201085C50] Kostrouchova, M., Krause, M., Kostrouch, Z. and Rall, J. E. (1998). CHR3: a *Caenorhabditis elegans* orphan nuclear hormone receptor required for proper epidermal development and molting. *Development* 125, 1617-1626. 10.1242/dev.125.9.1617 9521900

[DEV201085C51] Kostrouchova, M., Krause, M., Kostrouch, Z. and Rall, J. E. (2001). Nuclear hormone receptor CHR3 is a critical regulator of all four larval molts of the nematode *Caenorhabditis elegans*. *Proc. Natl. Acad. Sci. USA* 98, 7360-7365. 10.1073/pnas.131171898 11416209 PMC34673

[DEV201085C52] Kouns, N. A., Nakielna, J., Behensky, F., Krause, M. W., Kostrouch, Z. and Kostrouchova, M. (2011). NHR-23 dependent collagen and hedgehog-related genes required for molting. *Biochem. Biophys. Res. Commun.* 413, 515-520. 10.1016/j.bbrc.2011.08.124 21910973 PMC3196369

[DEV201085C53] Kramer, J. M. and Johnson, J. J. (1993). Analysis of mutations in the sqt-1 and rol-6 collagen genes of *Caenorhabditis elegans*. *Genetics* 135, 1035-1045. 10.1093/genetics/135.4.1035 8307321 PMC1205736

[DEV201085C54] Lam, G. T., Jiang, C. and Thummel, C. S. (1997). Coordination of larval and prepupal gene expression by the DHR3 orphan receptor during *Drosophila* metamorphosis. *Development* 124, 1757-1769. 10.1242/dev.124.9.1757 9165123

[DEV201085C55] Lažetić, V. and Fay, D. S. (2017). Molting in *C. elegans*. *Worm* 6, e1330246. 10.1080/21624054.2017.1330246 28702275 PMC5501215

[DEV201085C56] Lin, K., Dorman, J. B., Rodan, A. and Kenyon, C. (1997). *daf-16*: an HNF-3/forkhead family member that can function to double the life-span of *Caenorhabditis elegans*. *Science* 278, 1319-1322. 10.1126/science.278.5341.1319 9360933

[DEV201085C57] Macneil, L. T., Watson, E., Arda, H. E., Zhu, L. J. and Walhout, A. J. M. (2013). Diet-induced developmental acceleration independent of TOR and insulin in *C. elegans*. *Cell* 153, 240-252. 10.1016/j.cell.2013.02.049 23540701 PMC3821073

[DEV201085C58] Manders, E. M., Stap, J., Brakenhoff, G. J., van Driel, R. and Aten, J. A. (1992). Dynamics of three-dimensional replication patterns during the S-phase, analysed by double labelling of DNA and confocal microscopy. *J. Cell Sci.* 103, 857-862. 10.1242/jcs.103.3.857 1478975

[DEV201085C59] Manders, E. M. M., Verbeek, F. J. and Aten, J. A. (1993). Measurement of co-localization of objects in dual-colour confocal images. *J. Microsc.* 169, 375-382. 10.1111/j.1365-2818.1993.tb03313.x 33930978

[DEV201085C60] Meeuse, M. W. M., Hauser, Y. P., Morales Moya, L. J., Hendriks, G. J., Eglinger, J., Bogaarts, G., Tsiairis, C. and Großhans, H. (2020). Developmental function and state transitions of a gene expression oscillator in *Caenorhabditis elegans*. *Mol. Syst. Biol.* 16, e9975. 10.15252/msb.20209498 33438821 PMC7588024

[DEV201085C61] Meeuse, M. W. M., Hauser, Y. P., Nahar, S., Smith, A. A. T., Braun, K., Azzi, C., Rempfler, M. and Großhans, H. (2023). *C. elegans* molting requires rhythmic accumulation of the Grainyhead/LSF transcription factor GRH-1. *EMBO J.* 42, e111895. 10.15252/embj.2022111895 36688410 PMC9929640

[DEV201085C62] Mello, C. C., Kramer, J. M., Stinchcomb, D. and Ambros, V. (1991). Efficient gene transfer in *C. elegans*: extrachromosomal maintenance and integration of transforming sequences. *EMBO J.* 10, 3959-3970. 10.1002/j.1460-2075.1991.tb04966.x 1935914 PMC453137

[DEV201085C63] Melo, J. A. and Ruvkun, G. (2012). Inactivation of conserved *C. elegans* genes engages pathogen- and xenobiotic-associated defenses. *Cell* 149, 452-466. 10.1016/j.cell.2012.02.050 22500807 PMC3613046

[DEV201085C64] Mok, D. Z. L., Sternberg, P. W. and Inoue, T. (2015). Morphologically defined sub-stages of *C. elegans* vulval development in the fourth larval stage. *BMC Dev. Biol.* 15, 26. 10.1186/s12861-015-0076-7 26066484 PMC4464634

[DEV201085C65] Moribe, H., Yochem, J., Yamada, H., Tabuse, Y., Fujimoto, T. and Mekada, E. (2004). Tetraspanin protein (TSP-15) is required for epidermal integrity in *Caenorhabditis elegans*. *J. Cell Sci.* 117, 5209-5220. 10.1242/jcs.01403 15454573

[DEV201085C66] Nelson, F. K., Albert, P. S. and Riddle, D. L. (1983). Fine structure of the *Caenorhabditis elegans* secretory-excretory system. *J. Ultrastruct. Res.* 82, 156-171. 10.1016/S0022-5320(83)90050-3 6827646

[DEV201085C67] Nonet, M. L. (2020). Efficient transgenesis in *Caenorhabditis elegans* using Flp recombinase-mediated cassette exchange. *Genetics* 215, 903-921. 10.1534/genetics.120.303388 32513816 PMC7404237

[DEV201085C68] Page, A. P. and Johnstone, I. L. (2007). The cuticle. *WormBook*, 1-15. 10.1895/wormbook.1.138 PMC4781593 18050497

[DEV201085C69] Paix, A., Wang, Y., Smith, H. E., Lee, C.-Y. S., Calidas, D., Lu, T., Smith, J., Schmidt, H., Krause, M. W. and Seydoux, G. (2014). Scalable and versatile genome editing using linear DNAs with microhomology to Cas9 Sites in *Caenorhabditis elegans*. *Genetics* 198, 1347-1356. 10.1534/genetics.114.170423 25249454 PMC4256755

[DEV201085C70] Paix, A., Folkmann, A., Rasoloson, D. and Seydoux, G. (2015). High efficiency, homology-directed genome editing in *Caenorhabditis elegans* using CRISPR-Cas9 Ribonucleoprotein complexes. *Genetics* 201, 47-54. 10.1534/genetics.115.179382 26187122 PMC4566275

[DEV201085C110] Patel, R., Galagali, H., Kim, J. K. and Frand, A. R. (2022). Feedback between a retinoid-related nuclear receptor and the let-7 microRNAs controls the pace and number of molting cycles in C. elegans. *eLife* 11, e80010. 10.7554/eLife.80010 35968765 PMC9377799

[DEV201085C71] Posch, A., Kohn, J., Oh, K., Hammond, M. and Liu, N. (2013). V3 stain-free workflow for a practical, convenient, and reliable total protein loading control in western blotting. *J. Vis. Exp.* 82, 50948. 10.3791/50948 PMC4094170 24429481

[DEV201085C72] Pujol, N., Cypowyj, S., Ziegler, K., Millet, A., Astrain, A., Goncharov, A., Jin, Y., Chisholm, A. D. and Ewbank, J. J. (2008). Distinct innate immune responses to infection and wounding in the *C. elegans* epidermis. *Curr. Biol.* 18, 481-489. 10.1016/j.cub.2008.02.079 18394898 PMC2394561

[DEV201085C73] Qadota, H., Inoue, M., Hikita, T., Köppen, M., Hardin, J. D., Amano, M., Moerman, D. G. and Kaibuchi, K. (2007). Establishment of a tissue-specific RNAi system in *C. elegans*. *Gene* 400, 166-173. 10.1016/j.gene.2007.06.020 17681718 PMC3086655

[DEV201085C74] R Core Team (2007). *R: A language and environment for statistical computing*. R Foundation for Statistical Computing, Vienna, Austria. https://www.R-project.org/

[DEV201085C75] Ragle, J. M., Aita, A. L., Morrison, K. N., Martinez-Mendez, R., Saeger, H. N., Ashley, G. A., Johnson, L. C., Schubert, K. A., Shakes, D. C. and Ward, J. D. (2020). The conserved molting/circadian rhythm regulator NHR-23/NR1F1 serves as an essential co-regulator of *C. elegans* spermatogenesis. *Development* 147, dev193862. 10.1242/dev.193862 33060131 PMC7710015

[DEV201085C76] Ragle, J. M., Morrison, K. N., Vo, A. A., Johnson, Z. E., Hernandez Lopez, J., Rechtsteiner, A., Shakes, D. C. and Ward, J. D. (2022). NHR-23 and SPE-44 regulate distinct sets of genes during *C. elegans* spermatogenesis. *G3* 12, jkac256. 10.1093/g3journal/jkac256 36135804 PMC9635660

[DEV201085C78] Ruaud, A.-F. and Bessereau, J.-L. (2006). Activation of nicotinic receptors uncouples a developmental timer from the molting timer in *C. elegans*. *Development* 133, 2211-2222. 10.1242/dev.02392 16672334

[DEV201085C79] Ruaud, A.-F., Lam, G. and Thummel, C. S. (2010). The *Drosophila* nuclear receptors DHR3 and βFTZ-F1 control overlapping developmental responses in late embryos. *Development* 137, 123-131. 10.1242/dev.042036 20023167 PMC2796934

[DEV201085C80] Sandhu, A., Badal, D., Sheokand, R., Tyagi, S. and Singh, V. (2021). Specific collagens maintain the cuticle permeability barrier in *Caenorhabditis elegans*. *Genetics* 217, iyaa047. 10.1093/genetics/iyaa047 33789349 PMC8045729

[DEV201085C81] Sapio, M. R., Hilliard, M. A., Cermola, M., Favre, R. and Bazzicalupo, P. (2005). The Zona Pellucida domain containing proteins, CUT-1, CUT-3 and CUT-5, play essential roles in the development of the larval alae in *Caenorhabditis elegans*. *Dev. Biol.* 282, 231-245. 10.1016/j.ydbio.2005.03.011 15936343

[DEV201085C82] Schindelin, J., Arganda-Carreras, I., Frise, E., Kaynig, V., Longair, M., Pietzsch, T., Preibisch, S., Rueden, C., Saalfeld, S., Schmid, B. et al. (2012). Fiji: an open-source platform for biological-image analysis. *Nat. Methods* 9, 676-682. 10.1038/nmeth.2019 22743772 PMC3855844

[DEV201085C84] Singh, R. N. and Sulston, J. E. (1978). Some observations on molting in *C. elegans*. *Nematologica* 24, 63-71. 10.1163/187529278X00074

[DEV201085C85] Stec, N., Doerfel, K., Hills-Muckey, K., Ettorre, V. M., Ercan, S., Keil, W. and Hammell, C. M. (2021). An epigenetic priming mechanism mediated by nutrient sensing regulates transcriptional output during *C. elegans* development. *Curr. Biol.* 31, 809-826.e6. 10.1016/j.cub.2020.11.060 33357451 PMC7904604

[DEV201085C86] Stempor, P. and Ahringer, J. (2016). SeqPlots - Interactive software for exploratory data analyses, pattern discovery and visualization in genomics. *Wellcome Open Res.* 1, 14. 10.12688/wellcomeopenres.10004.1 27918597 PMC5133382

[DEV201085C87] Stojanovski, K., Großhans, H. and Towbin, B. D. (2022). Coupling of growth rate and developmental tempo reduces body size heterogeneity in *C. elegans*. *Nat. Commun.* 13, 3132. 10.1038/s41467-022-29720-8 35668054 PMC9170734

[DEV201085C88] Sulston, J. E. and Horvitz, H. R. (1977). Post-embryonic cell lineages of the nematode, *Caenorhabditis elegans*. *Dev. Biol.* 56, 110-156. 10.1016/0012-1606(77)90158-0 838129

[DEV201085C89] Tabara, H., Sarkissian, M., Kelly, W. G., Fleenor, J., Grishok, A., Timmons, L., Fire, A. and Mello, C. C. (1999). The *rde-*1 gene, RNA interference, and transposon silencing in *C. elegans*. *Cell* 99, 123-132. 10.1016/S0092-8674(00)81644-X 10535731

[DEV201085C90] Taubert, S., Ward, J. D. and Yamamoto, K. R. (2011). Nuclear hormone receptors in nematodes: Evolution and function. *Mol. Cell. Endocrinol.* 334, 49-55. 10.1016/j.mce.2010.04.021 20438802 PMC3042524

[DEV201085C91] Telford, M. J., Bourlat, S. J., Economou, A., Papillon, D. and Rota-Stabelli, O. (2008). The evolution of the Ecdysozoa. *Philos. Trans. R. Soc. B Biol. Sci.* 363, 1529-1537. 10.1098/rstb.2007.2243 PMC2614232 18192181

[DEV201085C92] Teuscher, A. C., Jongsma, E., Davis, M. N., Statzer, C., Gebauer, J. M., Naba, A. and Ewald, C. Y. (2019). The *in-silico* characterization of the *Caenorhabditis elegans* matrisome and proposal of a novel collagen classification. *Matrix Biol. Plus* 1, 100001. 10.1016/j.mbplus.2018.11.001 33543001 PMC7852208

[DEV201085C93] Tong, A., Lynn, G., Ngo, V., Wong, D., Moseley, S. L., Ewbank, J. J., Goncharov, A., Wu, Y.-C., Pujol, N. and Chisholm, A. D. (2009). Negative regulation of *Caenorhabditis elegans* epidermal damage responses by death-associated protein kinase. *Proc. Natl. Acad. Sci. USA* 106, 1457-1461. 10.1073/pnas.0809339106 19164535 PMC2629440

[DEV201085C94] Tsiairis, C. and Großhans, H. (2021). Chapter Two - Gene expression oscillations in *C. elegans* underlie a new developmental clock. In *Current Topics in Developmental Biology* (ed. S. Jarriault and B. Podbilewicz), pp. 19-43. Academic Press. 10.1016/bs.ctdb.2020.11.001 33992153

[DEV201085C95] Vo, A. A., Levenson, M. T., Ragle, J. M. and Ward, J. D. (2021). Efficient generation of a single-copy *eft-3p::TIR1::F2A:: BFP::AID*::NLS* allele in the *C. elegans ttTi5605* insertion site through recombination-mediated cassette exchange. *MicroPublication Biol.* 2021, 10.17912/micropub.biology.000425. 10.17912/micropub.biology.000425 PMC8335552 34355140

[DEV201085C96] Vogel, C. and Marcotte, E. M. (2012). Insights into the regulation of protein abundance from proteomic and transcriptomic analyses. *Nat. Rev. Genet.* 13, 227-232. 10.1038/nrg3185 22411467 PMC3654667

[DEV201085C97] Vuong-Brender, T. T. K., Suman, S. K. and Labouesse, M. (2017). The apical ECM preserves embryonic integrity and distributes mechanical stress during morphogenesis. *Development* 144, 4336-4349. 10.1242/dev.150383 28526752 PMC5769628

[DEV201085C98] Ward, J. D. (2015a). Rapid and precise engineering of the *Caenorhabditis elegans* genome with lethal mutation co-conversion and inactivation of NHEJ repair. *Genetics* 199, 363-377. 10.1534/genetics.114.172361 25491644 PMC4317648

[DEV201085C99] Ward, J. D. (2015b). Rendering the intractable more tractable: tools from *Caenorhabditis elegans* ripe for import into parasitic nematodes. *Genetics* 201, 1279-1294. 10.1534/genetics.115.182717 26644478 PMC4676526

[DEV201085C100] Ward, J. D., Bojanala, N., Bernal, T., Ashrafi, K., Asahina, M. and Yamamoto, K. R. (2013). Sumoylated NHR-25/NR5A regulates cell fate during *C. elegans* vulval development. *PLoS Genet.* 9, e1003992. 10.1371/journal.pgen.1003992 24348269 PMC3861103

[DEV201085C101] Ward, J. D., Mullaney, B., Schiller, B. J., He, L. D., Petnic, S. E., Couillault, C., Pujol, N., Bernal, T. U., Van Gilst, M. R., Ashrafi, K. et al. (2014). Defects in the *C. elegans* acyl-CoA synthase, *acs-*3, and nuclear hormone receptor, *nhr-*25, cause sensitivity to distinct, but overlapping stresses. *PLoS ONE* 9, e92552. 10.1371/journal.pone.0092552 24651852 PMC3961378

[DEV201085C102] Watts, J. S., Harrison, H. F., Omi, S., Guenthers, Q., Dalelio, J., Pujol, N. and Watts, J. L. (2020). New strains for tissue-specific RNAi studies in *Caenorhabditis elegans*. *G3* 10, 4167-4176. 10.1534/g3.120.401749 32943454 PMC7642939

[DEV201085C103] Zaslaver, A., Mayo, A. E., Rosenberg, R., Bashkin, P., Sberro, H., Tsalyuk, M., Surette, M. G. and Alon, U. (2004). Just-in-time transcription program in metabolic pathways. *Nat. Genet.* 36, 486-491. 10.1038/ng1348 15107854

[DEV201085C104] Zhang, C. and Ruvkun, G. (2012). New insights into siRNA amplification and RNAi. *RNA Biol.* 9, 1045-1049. 10.4161/rna.21246 22858672 PMC3551858

[DEV201085C105] Zhang, L., Ward, J. D., Cheng, Z. and Dernburg, A. F. (2015). The auxin-inducible degradation (AID) system enables versatile conditional protein depletion in *C. elegans*. *Development* 142, 4374-4384. 10.1242/dev.129635 26552885 PMC4689222

[DEV201085C106] Zugasti, O. and Ewbank, J. J. (2009). Neuroimmune regulation of antimicrobial peptide expression by a noncanonical TGF-β signaling pathway in *Caenorhabditis elegans* epidermis. *Nat. Immunol.* 10, 249-256. 10.1038/ni.1700 19198592

