# Supplementary material for: NHR-23 activity is necessary for C. elegans developmental progression and apical extracellular matrix structure and function
Source: Development. 2023 May 22;150(10):dev201085. doi: 10.1242/dev.201085 (PMC10233720; doi:10.1242/dev.201085)
Supplement: Supplementary information [file develop-150-201085-s1.pdf]

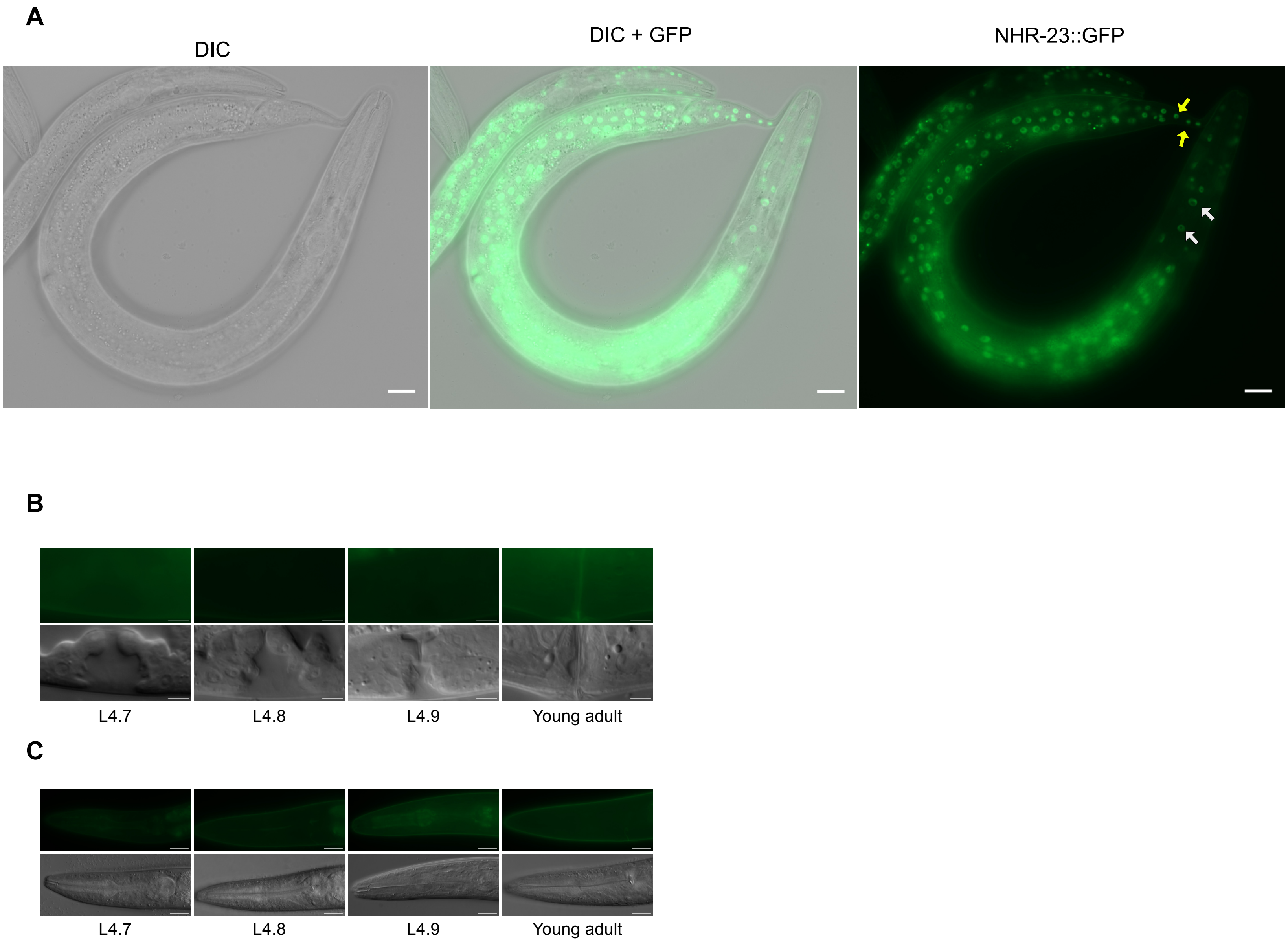

Fig. S1. NHR-23::GFP expression. (A) Representative image of NHR-23 expression in *nhr-23::GFP::AID*::3xFLAG; TIR-1* L4 animals. NHR-23 expression is seen in the hypodermis and developing germline, as described in Ragle et al. 2020. White arrows point to seam cells expressing GFP and yellow arrows point to hyp cells expressing GFP. NHR-23 expression in the vulva (B) and head (C) of *nhr-23::GFP::AID*::3xFLAG* animals of the indicated stages. Images are representative of 20 animals examined over 4 independent experiments. Scale bars=5 μm (B) or 20 μm (C). Animals were staged based on vulval morphology (Mok et al., 2015).

Development • Supplementary information

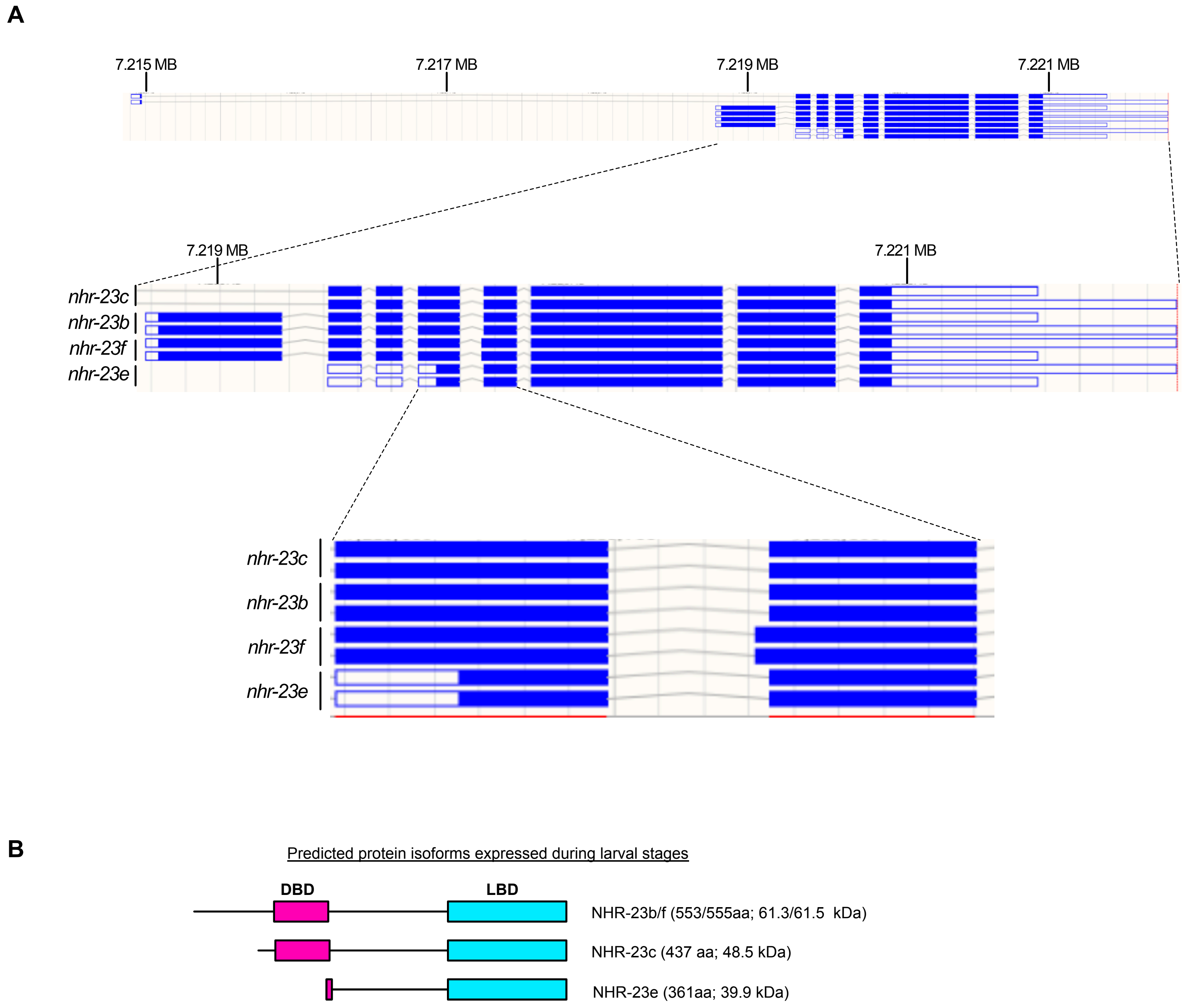

Fig. S2. *nhr-23* isoforms detected by Nanopore direct mRNA sequencing. (A) Gene model diagrams of the *nhr-23* isoforms from the "observed isoforms" track on the ENSEMBL genome track. These are isoforms confirmed by direct mRNA sequencing (Roach et al., 2020). The top tracks are of the full *nhr-23* gene, including the *nhr-23c* isoforms, which contain a large first intron. The middle tracks are a zoomed in view starting at the *nhr-23b* transcription start site. The bottom tracks are further zoomed in to depict the alternative 3' splice site usage that generates *nhr-23b* and *nhr-23f*. **(B)** Cartoons of predicted protein isoforms expressed during larval development based on Nanopore direct mRNA sequencing data (Roach et al., 2020). All of the predicted isoforms contain a Ligand Binding Domain (LBD) while only two of the three have a DNA Binding Domain (DBD). The predicted size of each isoform is listed to the right of the structure in amino acids (aa) and kDa. The AID*::3xFLAG fusion is predicted to add 9 kDa.

Development • Supplementary information

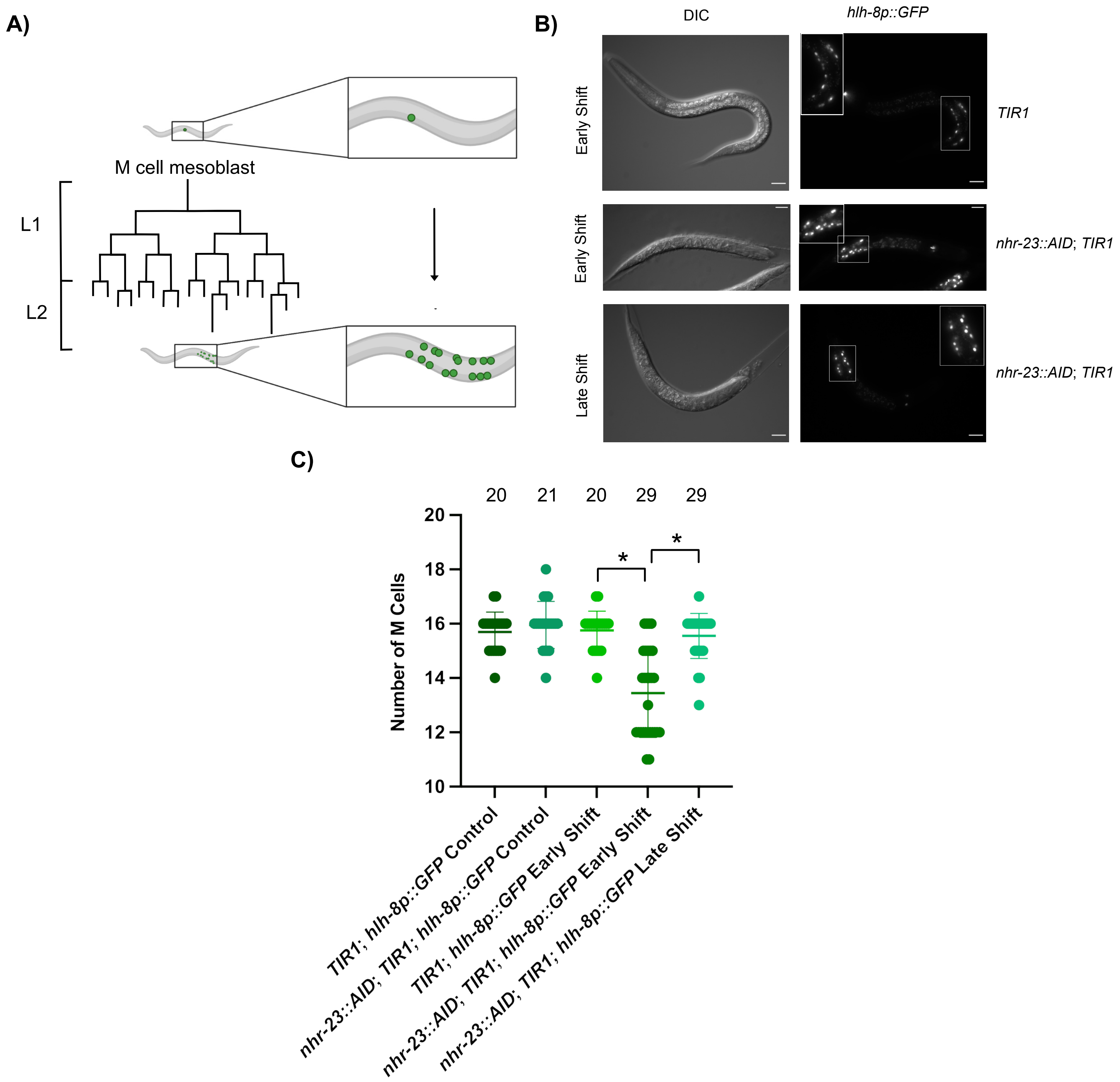

Fig. S3. NHR-23 depletion causes developmental delay (A) Depiction of the M cell mesoblast cell divisions in L1 larvae created with BioRender.com. Newly hatched animals are born with a single M cell, which undergoes the indicated set of divisions to produce 16 descendent cells at the L1/L2 molt. An *hlh-8p::GFP* reporter can be used to monitor M cell divisions in living animals. (B and C) Animals of the indicated genotype were synchronized and shifted to either auxin or control plates at either 3 (early shift) or 9 hours (late shift) post-release and imaged at 24 hours post-release. Two biological replicates were performed. (B) Representative images of animals of the indicated genotypes on auxin plates. (C) M cell quantification for animals of the indicated genotype shifted to control or auxin plates. Mean and standard deviation are labeled. The number of animals assayed for each genotype and condition is indicated at the top of the graph (* two-tailed T-test p<0.0001).

Development • Supplementary information

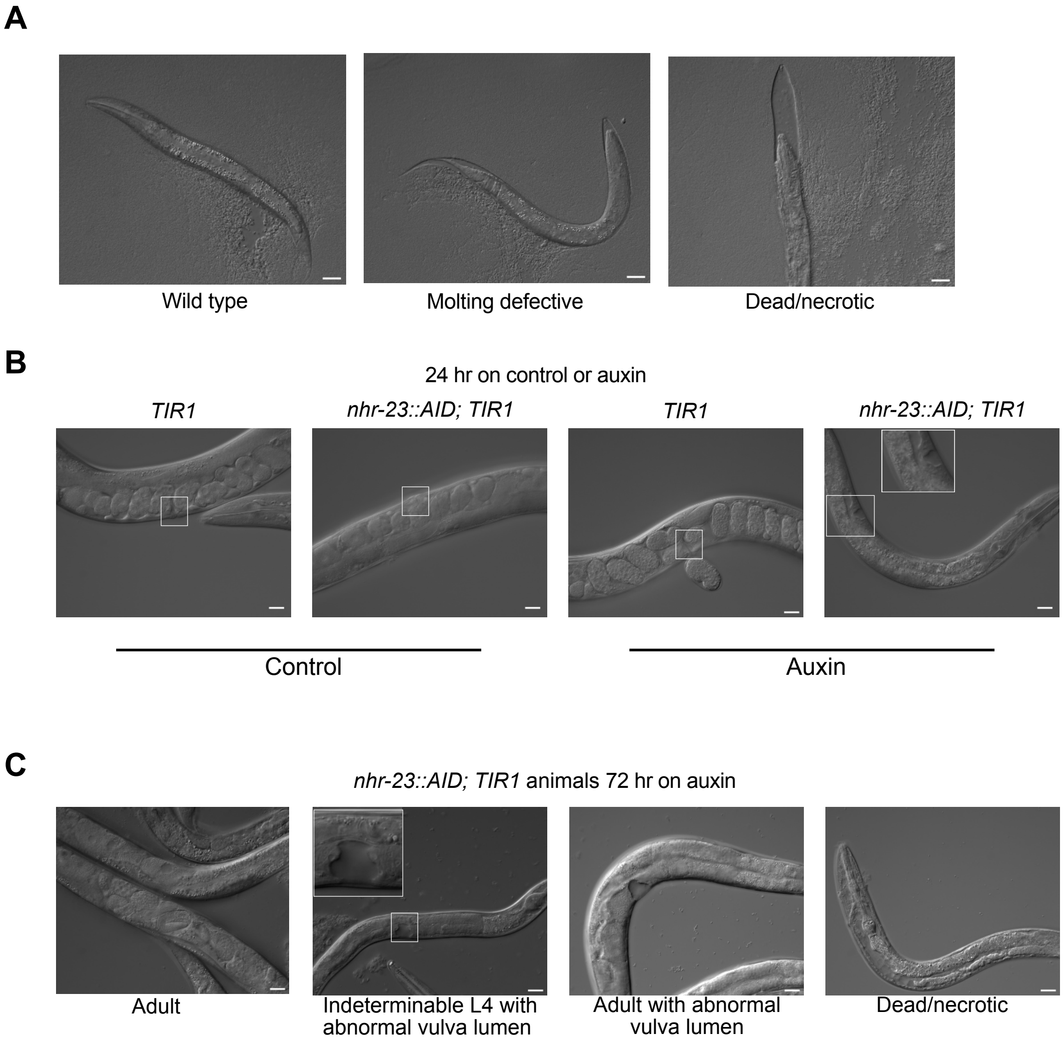

Fig. S4. NHR-23 depletion causes developmental delay. (A) Representative images of synchronized *nhr-23::AID; TIR1* L1 larvae released on auxin and scored for molting defects and death/necrosis 72 hours post-release. (B) Representative images of animals of the indicated genotype that were synchronized and shifted onto auxin at 25 hours post-release and imaged 23 hours later (48 hours post-release). The position of the vulva is highlighted with a white box. (C) Representative images of *nhr-23::AID; TIR1* animals treated as in (B) and scored after 72 hours on auxin. Scale bars=20 µm. A zoomed inset is provided for the auxin-treated *nhr-23::AID, TIR1* images in B and C to highlight vulval morphology.

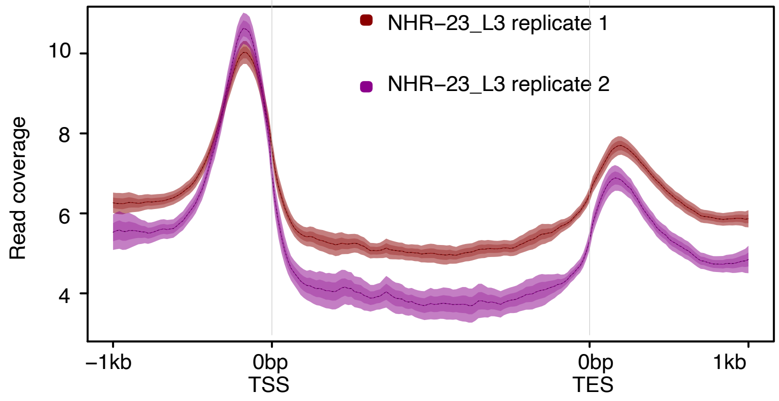

Fig. S5. NHR-23 enrichment around genes. Average signal over all aligned genes from two NHR-23 ChIP-seq replicates (Gerstein et al., 2010). The y-axis indicates non-normalized read coverage. The mean signal is plotted with a line and the mean's 95% confidence interval is indicated by the shaded area. TSS=transcription start site; TES=transcription end site.

A

Strain	Genotype	Brood size (±std. dev.)
N2	Wild type	290±48
JDW459	*nhr-23::AID; TIR1, rol-6::mNG::3xFLAG*	293±48
JDW463	*nhr-23::AID; TIR1, bli-1::mNG::3xFLAG(int)*	236±56
JDW475	*noah-1::mNG::3xFLAG(int), nhr-23::AID; TIR1*	246±27

B

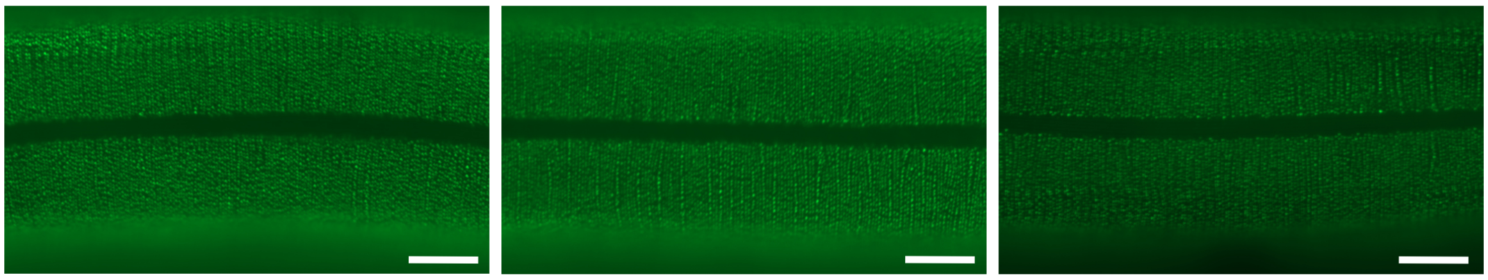

Fig. S6. Validation of *bli-1, noah-1,* and *rol-6* knock-in strains. (A) Brood size analysis of animals of the indicated genotype. (B) Representative images from L4.9 stage CZ27591 *bli-1(ju1789[bli-1::3xFLAG::mNG(int)]) II*, JDW389 *bli-1(wrd84[bli-1::mNG::3xFLAG(int)]) II*, and JDW463 *nhr-23(kry61(nhr-23::AID*-TEV-3xFLAG)) I; ieSi57 [Peft-3::TIR1::mRuby::unc-54 3'UTR, cb-unc-119(+)], bli-1(wrd84[bli-1::mNG::3xFLAG(int)])* animals. Equal 500 msec exposures from the same area of animals were taken using a 100x objective. Knock-ins are all at the same location in *bli-1* (see Fig. 6A). Scale bars=10 µm. Images are representative of 40 animals examined in two independent experiments.

Development • Supplementary information

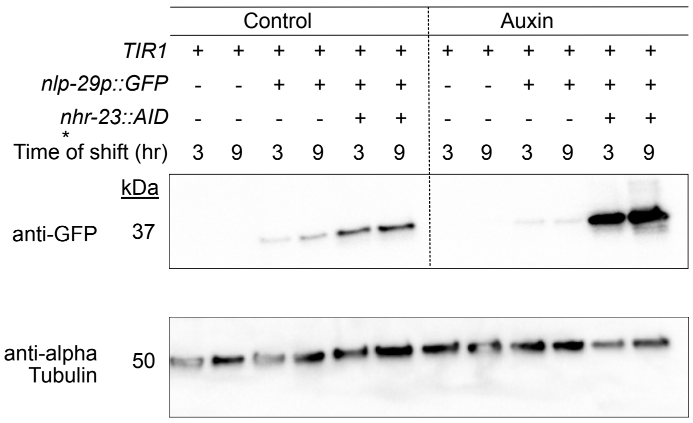

Fig. S7. Depletion of NHR-23 leads to a elevated *nlp-29p::GFP* reporter activity. Anti-GFP immunoblot analysis of *nlp-29::*GFP expression. Marker size (in kDa) is provided. An anti-tubulin immunoblot is provided as a loading control. The blots are representative of two experimental replicates.

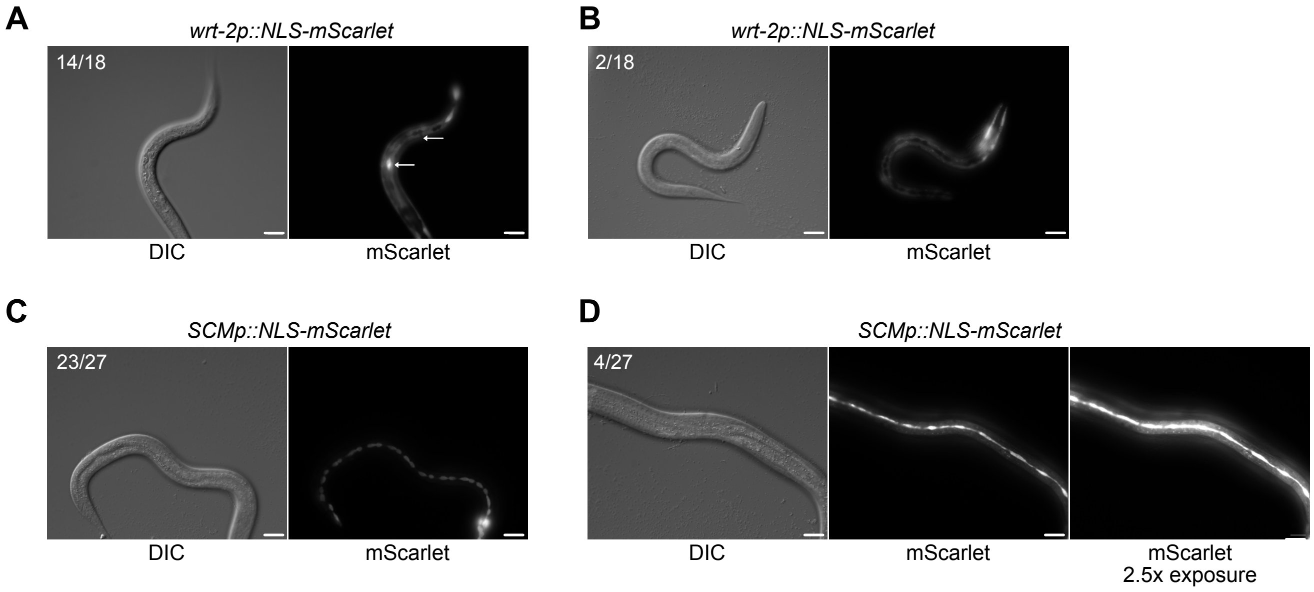

Fig. S8. Expression patterns of *SCMp* and *wrt-2* promoter reporters. *wrt-2p::NLS::mScarlet* is expressed in the hypodermis and seam cells (A and B). In some animals seam cell expression is lacking. In A, arrows point to two seam cells, one with reporter expression and one lacking it. *SCMp::NLS::mScarlet* promoter reporters express robustly in the seam cells (C) with weak expression in the hypodermis (D) in a subset of animals. Two biological replicates were performed and the number of animals for which each image is representative is indicated.

Development • Supplementary information

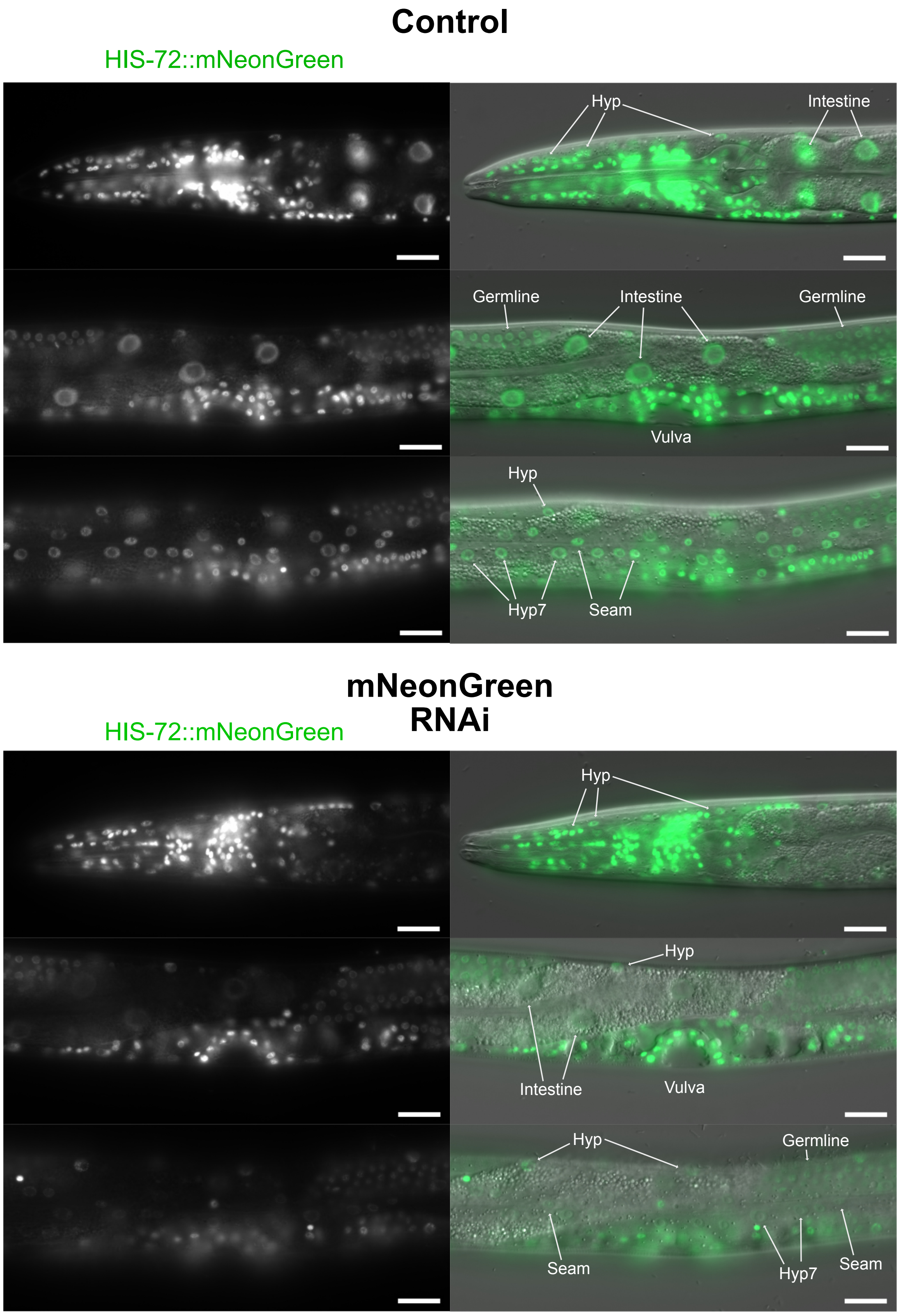

Fig. S9. JDW525 animals are permissive for RNAi knockdown in seam, hypodermal, and intestinal cells. JDW 525 [*his-72(wrd142[mNeonGreen::his-72]) III ; jsTi1493 {mosL loxP [wrdSi65(SCMp::rde-1 CDS +3'UTR)] FRT3::mosR} IV ; rde-1 (ne300) V]* animals grown on control RNAi display HIS-72::mNG expression in hypodermal, intestinal, germline, vulval, and seam cells. mNeonGreen RNAi caused reduction of HIS-72::mNG expression in seam, intestinal, and syncytial hypodermal (hyp7) cells. Vulval, germline, and head hypodermal expression was unaffected. Scale bars=20µm. Data are representative of forty animals examined over two biological replicates

Development • Supplementary information

Table S1. ***TIR1, nhr-23::GFP::AID*::3xFLAG*** **has milder developmental delay than** ***TIR1, nhr-23::AID*::3xFLAG*** **on auxin**

Genotype	Treatment	N	% Developmental delay
TIR1; nhr-23::AID::3xFLAG*	Control	296	0.34
	Auxin	237	100.00
TIR1; nhr-23::GFP::AID::3xFLAG*	Control	259	0.39
	Auxin	257	7.62

Animals were synchronized by a timed egg lay. Percent developmental delay is the number of animals that failed to reach L4 after 48 hours at 25ºC divided by the total number of animals scored, multiplied by 100%.

Table S2. ***nhr-23*****-regulated oscillating and non-oscillating genes**

Click here to download Table S2

Table S3. Gene ontology analysis of NHR-23-regulated oscillating genes

Gene Ontology (GO) term	Description	Enrichment	q-value
GO:0042302	structural constituent of cuticle	7.54	2.23E-07
GO:0042329	structural constituent of collagen and cuticulin-based cuticle	31.06	1.80E-05
GO:0005198	structural molecule activity	3.21	6.33E-03
GO:0004867	serine-type endopeptidase inhibitor activity	5.73	3.97E-01
GO:0008237	metallopeptidase activity	3.78	3.37E-01

Enrichment: observed/expected occurrence of a GO term in the *nhr-23*-regulated gene set
q value: p value adjusted for false discovery rate

Table S4. Gene ontology analysis of all oscillating genes

Click here to download Table S4

Table S5. NHR-23 ChIP-seq peaks broken down by position relative to coding sequence.

Click here to download Table S5

Development • Supplementary information

Table S6. crRNAs used for this study

Number	Target	crRNA sequence (5'-3', PAM not included)
1	*his-72* N-terminus	GGTACGAGCCATTGTTGTTC
51	*noah-1* internal	GTTGAGTGTGCTTTAACCGG
318	*bli-1* internal knock-in	AGATTCATCTTCTGCAGCTT
351	*rol-6* C-terminus	GTCATGAATTTGTCTACAAT

Table S7. PCR primers used for knock-ins and genotyping.

Click here to download Table S7

Table S8. Plasmids used in this study

Plasmid	Cloning approach	Source	Purpose
pLF3FShC	N/A	Nonet, 2020	Integration vector for RMCE
pJW849	Gibson	This study	*wrt-2p:: NLS-mScarlet (dpi)::tbb-2 3'UTR* promoter reporter
pJW1841	N/A	Ashley et al., 2021	promoterless *NLS::mScarlet (dpi)::tbb-2 3'UTR* vector
pJW1940	Gibson	This study	*SCMp::NLS-mScarlet (dpi)::tbb-2 3'UTR* promoter reporter
pJW2172	N/A	Ashley et al., 2021	30xlinker:mNeonGreen (dpi)::3xFLAG::10xlinker repair template
pJW2247	Gibson	This study	Promoterless rde-1 CDS+3'UTR in RMCE integration vector. Linearize vector with BsiWI and AvrII double digestion to Gibson clone in new promoters
pJW2236	Gibson	This study	*SCMp::rde-1 CDS+3'UTR* in RMCE integration vector for seam cell-specific RNAi
pJW2264	Gibson	This study	*semo-1p::rde-1 CDS+3'UTR* in RMCE integration vector for hypodermis-specific RNAi

Development • Supplementary information
