# Supplementary material for: NHR-23 activity is necessary for C. elegans developmental progression and apical extracellular matrix structure and function
Source: Development. 2023 May 22;150(10):dev201085. doi: 10.1242/dev.201085 (PMC10233720; doi:10.1242/dev.201085)
Supplement: Supplementary file 1 [file develop-150-201085_review_history.pdf]

NHR-23 activity is necessary for C. elegans developmental progression and apical extracellular matrix structure and function

Londen C. Johnson, An A. Vo, John C. Clancy, Krista M. Myles, Murugesan Pooranachithra, Joseph Aguilera, Max T. Levenson, Chloe Wohlenberg, Andreas Rechtsteiner, James Matthew Ragle, Andrew D. Chisholm and Jordan D. Ward
DOI: 10.1242/dev.201085

Editor: Swathi Arur

Review timeline

Original submission:	26 October 2021
Editorial decision:	29 November 2021
Resubmission:	7 July 2022
Editorial decision:	28 July 2022
First revision received:	13 March 2023
Editorial decision:	4 April 2023
Second revision received:	4 April 2023
Accepted:	11 April 2023

Original submission

First decision letter

MS ID#: DEVELOP/2021/200301

MS TITLE: Conditional depletion reveals temporal requirements for the oscillating transcription factor NHR-23/NR1F1 in *C. elegans* larval progression

AUTHORS: Londen C Johnson, Joseph L Aguilera, Max T Levenson, Andreas Rechtsteiner, An A Vo, James Matthew Ragle, and Jordan David Ward

I have now received all the referees' reports on the above manuscript, and have reached a decision. As you will see, the referees express interest in your work, but have some significant critiques and present recommendations that require both a careful rewrite of the manuscript, and also some additional experiments. In particular, reviewer 3's suggestion of driving NHR-23 in a continuous manner and uncoupling its expression from oscillation to directly test the role of oscillation on the phenotype observed is an important one to address. Reviewer 2 makes excellent suggestions on defining when, where and how NHR-23 may function to regulate molting. In particular, given the broad expression pattern of NHR-23, defining its autonomy in the tissue of interest is important, and as suggested by the reviewer connecting a network of targets identified to the define the "how" will greatly enhance our understanding. Both reviewer 1 and 2 make excellent suggestions on overall readability of the manuscript as well as better defining the experimental paradigms to increase the rigor in the analysis.
Your revised paper will be re-reviewed by one or more of the original referees, and acceptance of your manuscript will depend on your addressing satisfactorily the reviewers' major concerns.

Reviewer 1

Advance Summary and Potential Significance to Field

© 2023. Published by The Company of Biologists under the terms of the Creative Commons Attribution License (https://creativecommons.org/licenses/by/4.0/).

This manuscript by Johnson et al. describes significant advancements in understanding the function of oscillating expression of the nuclear hormone receptor NHR-23 in C. elegans larval development. NHR-23 was previously shown to be essential for embryonic development and larval molting, but the temporal requirement for NHR-23 was not explored. Here the authors provide compelling evidence that pulses of NHR-23 are necessary for molting and progression to the next larval stage. For these experiments, they use both constitutive and tissue-specific auxin-induced degradation of the protein product of a novel nhr-23 allele. They further demonstrate that NHR-23 functions independently of the well characterized DAF-16/insulin signaling pathway during larval development. Finally, they mine existing datasets of gene expression and NHR-23 ChIP assays to identify a large set of putative NHR-23 targets that also show oscillating expression. The work is generally very well done, and it will impact our understanding of the genes involved in periodic tissue remodeling.

Comments for the author

Essential comments:
The authors make the interesting observation that NHR-23 depletion beginning early in a given larval stage produces less severe defects than depletion beginning late in that stage. This is surprising given that once the auxin-induced depletion regimen was started in these experiments, it was maintained until the end of this larval stage. Therefore, animals depleted early should also be depleted late. While it is clear that auxin treatment leads to rapid NHR-23 depletion (Figure 2B), the authors should show using similar western blot analysis that NHR-23 levels remain low in these early auxin-treated L1 animals throughout the remainder of this larval stage. If so, I hope that the authors would speculate in the discussion on possible mechanisms for this surprising observation.

There were also formatting problems with the supplementary Tables S2 and S3 in the pdf of the Supplementary Material. This made it difficult to follow the section in the Discussion beginning on line 458, and I suggest including these Tables as Excel worksheets (similar to Table S1).

Additional minor comments:
The authors state in the abstract and elsewhere that C. elegans essentially build a new epidermis or skin at each larval stage, whereas the epidermis is actually remodeled to produce a new cuticle. I feel a more precise description of these events would be beneficial.

Figure 1: The NHR-23::AID::3xFLAG protein bands seem to migrate at a higher MW than I expected, given that the MW of the untagged NHR-23 isoforms are predicted to be ~40-65 kDal. How big is the AID::3x FLAG tag?

Figure 2B: The time labels in the auxin treated samples are misaligned in the pdf. It looks like it takes 30 minutes for NHR-23 to be degraded.

Results section beginning line 185 Table 1: The results for gut TIR expression using ges-1p are not shown in Table 1. Could you examine larval arrest and molt defects in strains expressing TIR in combinations or tissues (e.g., gut and seam cells) in strains with multiple TIR expressing constructs?

Figure 3B: Add labels indicating early or late auxin treatment. I think that's why there are 2 lanes for each condition, but it's not clear.

Figure 4B: It's hard to distinguish between some of the larval stages in the bar graph using the different color shades of blue. Maybe use a different set of colors?

Figure 6A: not referred to in the text.

Figure 6B: Indicate what the TSP and TES abbreviations mean in the figure legend. I saw that you did this in the supplemental figures.

Figure S1: The DIC and DIC+GFP labels are switched in the figure.

© 2023. Published by The Company of Biologists under the terms of the Creative Commons Attribution License (https://creativecommons.org/licenses/by/4.0/).

Reviewer 2

Advance Summary and Potential Significance to Field

C. elegans NHR-23 is a nuclear hormone receptor of the RORalpha family - both NHR-23 and its insect relative DHR3 have long been known to affect molting and the expression of various matrix genes. However, many questions remain about how these NHRs fit into regulatory networks that coordinate all the different steps of matrix assembly and disassembly so that they happen at the appropriate times. Here Johnson et al look more closely into NHR-23 expression patterns and genetic requirements by building an endogenously tagged strain and a degron allele. The new insights described are: 1) NHR-23 protein levels oscillate in a pattern similar to that previously described for the mRNA; 2) NHR-23 depletion at different points in the molt cycle can result in different morphological or arrest phenotypes; 3) An annotated set of candidate nhr-23 target genes is generated based on re-analysis of previously published RNA profiling and ChIP-Seq data.

Comments for the author

As detailed below, there are some issues with data documentation and clarity that should be easily addressable by the authors. Most experiments appear to be performed rigorously, with appropriate controls, as I would expect from the senior author's track record. My main concern, especially with respect to this journal, is that the advance is still fairly small and doesn't yet allow strong conclusions to be drawn about when, where, or how NHR-23 functions to promote molting progression. As the authors point out, their tools will be valuable for future studies, but tackling one or more of those key questions here would increase impact.

Major points

1. When? The data here confirm that NHR-23 protein levels peak in the middle of each larval stage, and that mRNAs for most NHR-23-regulated genes peak sometime after that, as should be expected. However, it would be nice if the ChIPseq analyses were better integrated with the other data (e.g. in Figure 6 and Table S1), so that we could see which NHR-23 regulated genes are most likely to be direct vs. indirect targets. This might give us a clearer idea of whether NHR-23 directly regulates different biologically related sets of genes at different timepoints (as the authors seem to speculate in Discussion), and also help with interpretation of the phenotypes seen in the depletion experiments (related to point 3).

2. Where? nhr-23 is expressed fairly broadly, and the authors don't address cell autonomy vs. non-autonomy or where nhr-23 acts to affect developmental progression or molting. This seems particularly important given some puzzling negative data with the degron allele. That data should probably be removed if the explanation can't be determined. Tissue-specific rescue experiments or mosaic analysis could get at the question.

3. How? The phenotypes seen after nhr-23 depletion are not examined in much detail, and although a variety of nhr-23 targets have been known for years (and some particularly good candidates are identified here), the authors do not demonstrate failure to express any of them to try to tie any target gene(s) to the different morphological phenotypes observed after nhr-23 knockdown. This makes different parts of the paper feel a bit disconnected or speculative and hampers a clear take home message. Examining some target genes after the different knockdown regimens could add a lot to the paper.

4. There are many places where n= information for images or Western blot replicates is missing from the legend, statistical tests are unexplained, or where data are described as "not shown". Specific examples are listed in the detailed comments below.

Specific points (in order of appearance)

1. Lines 24-25 (Abstract): "Nematode molting is a remarkable process where the animals must essentially build a new epidermis underneath the old skin and then rapidly shed the old skin."

© 2023. Published by The Company of Biologists under the terms of the Creative Commons Attribution License (https://creativecommons.org/licenses/by/4.0/).

This sentence is misleading and should be modified - the cuticle is an extracellular matrix distinct from the cellular epidermis - and I am not aware of any evidence that the cells in the epidermis must be re-built.

2. The introduction raises many questions, most of which are not going to be addressed in this study, and the Discussion also is far-ranging. I would encourage the authors to more tightly refocus the text on the questions re: timing and targets that they actually tried to address here.

3. Figure 1, Missing data details

Figure 1B - images are representative of HOW MANY animals examined at each timepoint? This info should be in the legend.

Figure 1C - these blots are representative of how many experimental replicates? This info should be in the legend (preferably) or in the Methods.

4. Figure 2, Missing data details

Figure 2B - these blots are representative of how many experimental replicates?

Figure 2D - Please provide some quantification of the arrest phenotype under the various regimens. Are all of the animals arrested after the early (2hr) AID shift?

5. Line 172: "molting defects were low (0-5%) when animals were shifted to auxin between 2-4 hours post release (Fig. 2D)." Does "2-4 hours" correspond to "2 hours" and/or "4 hours" in the figure? This is confusing because the numbers in text and figure don't seem to match.

6. Lines 175-176, related to Figure 2. "These data could suggest that NHR-23 may not be necessary for the initiation of the molt but required for completion of the molt."

It would be helpful if the authors were more explicit about how they define "molt initiation". Lethargus? Apolysis? Those are the classic criteria, but perhaps outdated. Expression of transient sheath components and early/furrow collagens begins in the middle of each larval stage, and perhaps this is the real beginning of the molt. This last idea is discussed in recent reviews (https://pubmed.ncbi.nlm.nih.gov/33036165/ and (https://pubmed.ncbi.nlm.nih.gov/33992153/).

7. Related to Figures 2 and 3 and point 6. Not enough data are provided to know if arrest is due to failure to enter the molt in the first place, or if animals are stuck at an early step of the molt, before apolysis. There are behavioral and morphological features (e.g. activity levels, pharyngeal pumping, the buccal plug) and molecular markers (e.g. mlt-10::GFP::pest, others) that could distinguish these. (major point 3)

The nlp-29::GFP result (Fig. 3) suggests to me that some amount of cuticle disassembly has begun in the arrested animals, which would mean that animals have initiated the molt, but it would be better to assess this more directly.

8. Table 1 and Lines 196-201: "Only depletion using the vha-8p::TIR1 transgene was able to phenocopy the pan-somatic TIR1 allele (eftp::TIR1) (Table 1). These data could reflect: i) incomplete depletion of NHR-23 in hypodermis, intestine, neurons, or seam cells; ii) NHR-23 is not necessary in those cell types; iii) NHR-23 activity is required in multiple tissues, with single-tissue depletion being insufficient to disrupt molting."

No n= information is provided here and the data are not informative. Table could be removed or made supplemental.

This is where I suspect a degron approach might not be ideal for a nuclear protein. But the models and issues of cell autonomy vs. non-autonomy might be distinguishable using complementary tissue-specific rescue experiments or mosaic analysis. Otherwise, unfortunately there is not much we can conclude from the data. (major point 2)

© 2023. Published by The Company of Biologists under the terms of the Creative Commons Attribution License (https://creativecommons.org/licenses/by/4.0/).

9. Figure 3, Missing data details

Figure 3A - images are representative of HOW MANY animals examined? What do the asterisks mean? Are the "early shift" animals the L2 arrested animals after 2hr shift? I'm not sure how this relates to Fig. 2.

Figure 3B - these blots are representative of how many experimental replicates?

As mentioned above, activation of the cuticle damage sensor is consistent with idea that, under both "early" and "late" regimens, animals initiate the molt and then get stuck. More markers of the different steps in the molt cycle could get at what is happening. (major point 3)

10. Figure 4, Missing data details

Figure 4C - what do the asterisks mean and what statistical test was used for comparisons?

Figure 4E - images are representative of HOW MANY animals examined?

11. Related to Figure 4. Could developmental arrest be caused by occlusion of the buccal cavity so that the animals can't feed? The Frand lab has described a "plug" that forms there early in the molt (unfortunately unpublished). The Karp lab described a nice fluorescent bead assay for feeding: https://pubmed.ncbi.nlm.nih.gov/27172224/

12. related to Figures 4 and 5 - The magnification is too low to see the vulva clearly, but even without nhr-23::AID, the TIR1 animals look abnormal, with advanced embryos inside. What's going on there?

13. Lines 263-265: What is mid late L4? (typo?)

14. Figure 5 and Lines 274-315: "Larval arrest following NHR-23 depletion is independent of the insulin signaling pathway"

There is too much space dedicated to this negative result - it is the sort of thing that belongs in the supplemental material

Figure 5A - what do the asterisks mean and what statistical test was used for comparisons?

15. Figure 6 and Table S1 (re-analysis/synthesis of published RNA profiling and ChIP data) contain some of the most interesting data in the paper, but presentation is not as useful as it could be.

Fig. 6B ChIP analysis would be more valuable if confidently-bound target genes were plotted as a comparison to Fig. 6C or integrated into the annotation in Table S1 (major point 1)

How do the nhr-23-enriched GO-terms differ from those of oscillating genes as a whole? (a large proportion of oscillating genes are matrix-related, but are you seeing anything particularly unique in nhr-23-enriched vs. non-enriched oscillating genes?)

For Fig. 6C, consider subdividing "aECM components" to tie into the emerging biology of assembly/disassembly. e.g., Where do transient sheath factors, furrow collagens, and other classic "early" vs "late" cuticle collagens show up here?

Finally, could the authors look at some representative targets from these classes (e.g. with existing reporter fusions) to more precisely determine where the block is in their different phenotypic classes of nhr-23 knockdown animals? (major point 3)

16. Fig. 6B, modENCODE should be cited in main text and legend as the source of the ChIPseq data. The prior text sentence seems to cite modENCODE for oscillation, not for ChIPseq.

© 2023. Published by The Company of Biologists under the terms of the Creative Commons Attribution License (https://creativecommons.org/licenses/by/4.0/).

17. Lines 334-336. "There are also several sets of genes involved in apical extracellular matrix biogenesis and remodeling, such as lipocalins, proteases, protease inhibitors, fibrillin, PAN and ZP domain-containing proteins, and leucine-rich repeat proteins. "

This is the first mention of most of these proteins/domains, so acronyms should be defined. It would be helpful to provide references or other information to support the statement that these genes/proteins are involved in matrix biogenesis, or to help reader understand how they relate to cuticle (most are not actually part of the cuticle, they are associated with the transient sheath).

18. Table S1: would be much more useful if ChIP data were also annotated here. (major point 1)

19. There are 4 places in the manuscript where "unpublished data" are cited. All data should be either shown or not mentioned.

20. Figure S1: DIC and DIC+GFP labels are reversed

21. Figure S3: It would be useful if definitions for "early", "mid", and "late" L4 were included in this legend rather than referring to Fig. 4.

22. Figure S4: The data in Figure 5 could go here.

23. Figure S8 and Table S2 feel quite peripheral to this story

24. line 874 space missing

Reviewer 3

Advance Summary and Potential Significance to Field

The manuscript by Johnson et al. describes oscillations in expression of apical ECM components seen during larval molt in Caenorhabditis elegans. The authors attempt to connect these oscillations to oscillation in the protein level expression of a nuclear hormone receptor, NHR-23. The oscillations for nhr-23, during larval molts, have been reported at transcript levels previously. The author show oscillation at the protein levels using an NHR-23::GFP reporter.
Following this, the auathors use an auxin inducible degron system to deplete NHR-23 during and past the first larval molt. This allows authors to indicate the requirement of NHR-23 in a narrow time window, nicely done. Following this the authors propose that NHR-23 is needed to maintain cuticle barrier, however this is a weak part of the study (see major comments and suggestions) because of use of nlp-29 as reporter for barrier function. The authors suggest that the molting defect of nhr-23 depletion is not linked to DAF-16. The authors also mine CHIP and gene expression data from previous studies (from various labs) to correlate the oscillation in NHR-23 expression with oscillations in expression of genes encoding apical ECM components, collagens etc. I commend the authors on representation of the data in the first part of Figure 6. This however falls short of confirmation without experimental evidence that preventing oscillation of NHR-23 affects oscillations in aECM components. Doing that will make it a really nice and wholesome study beyond correlations. The manuscript is written nicely, it is easy to understand and follow.

Comments for the author

Major comments
Figure 1. Number of animals examined for showing oscillation of NHR-23::GFP is not indicated. Biological and technical replicated should be indicated. How many animals at a certain time point show oscillations? These numbers would boost confidence in the data. Table 1 fails to mention number of experiments, population size, faction of animals with phenotypes etc. Depletion of NHR-23 leads to a defective cuticle barrier: I disagree with the use of Pnlp-29::GFP as a reporter for cuticle barrier dysfunction. This reporter can not be used as proxy for barrier function defect. NLP-29 is believed to be an antimicrobial factor, induced in response to various assaults to the epidermis including including infection, chemicals and physical injury. It responds both to chronic damage as well as acute stress. Breach of this barrier is unlikely to have happened during osmotic

© 2023. Published by The Company of Biologists under the terms of the Creative Commons Attribution License (https://creativecommons.org/licenses/by/4.0/).

stress or tunicamycin treatment. Authors must use a direct measure for barrier function such as Hoechst stain permeability, AFM or SEM analysis of the cuticle. Authors show correlation etween the wave of NHR-23 protein expression and molting (Figure 6). To conclude that NHR-23 oscillation is responsible for aECM formation at each molt, authors must disrupt the oscillations of NHR-23 and maintain it at high levels. This can be done by keeping NHR-23 ON under a non oscillating promoter such as vha-8p. If authors hypothesis is correct, keeping NHR-23 ON should lead to loss of oscillation in aECM components as well. This experiment is absolutely essential for this study.
Authors should make use transcript and reporter (for aECM components) analysis for these assays.

Other Comments

NHR-23 is required in hypodermis, intestine, neurons, or seam cells. This should be discussed. Are authors invoking non cell autonomous events and communication between these tissues. When does this happen?

Scale bar in Figure 1 should be mentioned in the legend.

Is the oscillation of NHR-23 dependent on oscillation of BLMP-1 expression or function?

Line 51: This group of pathogens infect over one billion humans and devastate crops and livestock, yet few drugs exist to counter them and resistance is emerging (Ward, 2015b).
This can be split and reworded, for example to "parasitic nematodes cause over a billion human infections each year and loss of livestock and crops. However, very few drugs exist and resistance to those drugs is emerging rapidly.

In Auxin exposure regime, animals were maintained on auxin until imaging. Why aren't the abnormalities seen in that case even on continuous exposure to auxin?
Later it is mentioned that animals with this much exposure show developmental arrest. Rescue experiments by removing the animals from auxin after early exposure and late exposure to confirm stage specific effects.

Data in Fig 2D can be better represented as a histogram.

The significance is shown in terms of CI and sometimes as p value. It is confusing. Representation as p-value will help in easy interpretation.

First revision

Author response to reviewers' comments

We thank the reviewers for their helpful and constructive suggestions, which drastically improved this manuscript. We have provided a point-by-point response to their comments below.

>>>

Reviewer 1 Comments for the Author:
Essential comments:
The authors make the interesting observation that NHR-23 depletion beginning early in a given larval stage produces less severe defects than depletion beginning late in that stage. This is surprising given that once the auxin-induced depletion regimen was started in these experiments, it was maintained until the end of this larval stage. Therefore, animals depleted early should also be depleted late. While it is clear that auxin treatment leads to rapid NHR-23 depletion (Figure 2B), the authors should show using similar western blot analysis that NHR-23 levels remain low in these early auxin-treated L1 animals throughout the remainder of this larval stage. If so, I hope that the authors would speculate in the discussion on possible mechanisms for this surprising observation.

© 2023. Published by The Company of Biologists under the terms of the Creative Commons Attribution License (https://creativecommons.org/licenses/by/4.0/).

>>> We have performed a time-course western blot which demonstrated both a rapid depletion of NHR-23 and continued low levels after up to 16 hours on auxin. This data is in Figure 2. We have added additional data and discussion regarding the surprising observation that early NHR- 23 depletion produced milder molting defects. We suggest that early NHR-23 depletion causes severe developmental delay and it took multiple days for animals to attempt to molt, at which point we see similar defects as in the late auxin shift experiments.

There were also formatting problems with the supplementary Tables S2 and S3 in the pdf of the Supplementary Material. This made it difficult to follow the section in the Discussion beginning on line 458, and I suggest including these Tables as Excel worksheets (similar to Table S1).
>>> We removed Table S2 per Reviewer 2's suggestion and provided what was Table S3 in the original submission as an excel worksheet.

Additional minor comments:
The authors state in the abstract and elsewhere that C. elegans essentially build a new epidermis or skin at each larval stage, whereas the epidermis is actually remodeled to produce a new cuticle. I feel a more precise description of these events would be beneficial.
>>>Reviewer 2 also commented on this point and we have re-worked the introduction to more clearly describe the apical ECM structure.

Figure 1: The NHR-23::AID::3xFLAG protein bands seem to migrate at a higher MW than I expected, given that the MW of the untagged NHR-23 isoforms are predicted to be ~40-65 kDal. How big is the AID::3x FLAG tag?
>>> We appreciate the reviewer catching this issue. The AID*::3xFLAG tag is ~ 9 kDa so the predicted NHR-23 isoforms would then 49-74 kDa. The upper band is indeed migrating higher than expected. We have added text to the Results to discuss the size discrepancy and reduced the discussion about the NHR-23e isoform. We have also clarified the predicted isoform size and AID*::3xFLAG tag size in Fig 1 and it's legend. Given the uncertainty about what isoforms the bands represent, we have removed the section about the isoforms from the Discussion.

Figure 2B: The time labels in the auxin treated samples are misaligned in the pdf. It looks like it takes 30 minutes for NHR-23 to be degraded.
>>> We have corrected this issue.

Results section beginning line 185 Table 1: The results for gut TIR expression using ges-1p are not shown in Table 1. Could you examine larval arrest and molt defects in strains expressing TIR in combinations or tissues (e.g., gut and seam cells) in strains with multiple TIR expressing constructs?
>>>We have switched to a tissue-specific RNAi approach to address where nhr-23 activity is necessary (Fig. 8). These data suggest that NHR-23 is necessary in hypodermal and seam cells.

Figure 3B: Add labels indicating early or late auxin treatment. I think that's why there are 2 lanes for each condition, but it's not clear.
>>> Thank you for catching this omission; we have corrected this issue. This data is now in Figure 7C.

Figure 4B: It's hard to distinguish between some of the larval stages in the bar graph using the different color shades of blue. Maybe use a different set of colors?
>>>We based our color coding on those used by the Walhout lab (MacNeil et al., 2013) in profiling the role of NHR-23 in diet-induced developmental acceleration. We'd prefer to keep the color coding for consistency in the literature.

Figure 6A: not referred to in the text.
>>> We have added a reference to this Figure, which is now Fig 4A.

Figure 6B: Indicate what the TSP and TES abbreviations mean in the figure legend. I saw that you did this in the supplemental figures.
>>> We have corrected this issue.

Figure S1: The DIC and DIC+GFP labels are switched in the figure.

© 2023. Published by The Company of Biologists under the terms of the Creative Commons Attribution License (https://creativecommons.org/licenses/by/4.0/).

>>> We have corrected this issue.

Reviewer 2 Comments for the Author:
As detailed below, there are some issues with data documentation and clarity that should be easily addressable by the authors. Most experiments appear to be performed rigorously, with appropriate controls, as I would expect from the senior author's track record. My main concern, especially with respect to this journal, is that the advance is still fairly small and doesn't yet allow strong conclusions to be drawn about when, where, or how NHR-23 functions to promote molting progression. As the authors point out, their tools will be valuable for future studies, but tackling one or more of those key questions here would increase impact.

Major points

1. When? The data here confirm that NHR-23 protein levels peak in the middle of each larval stage, and that mRNAs for most NHR-23-regulated genes peak sometime after that, as should be expected. However, it would be nice if the ChIPseq analyses were better integrated with the other data (e.g. in Figure 6 and Table S1), so that we could see which NHR-23 regulated genes are most likely to be direct vs. indirect targets. This might give us a clearer idea of whether NHR-23 directly regulates different biologically related sets of genes at different timepoints (as the authors seem to speculate in Discussion), and also help with interpretation of the phenotypes seen in the depletion experiments (related to point 3).

>>> We have analyzed where the ChIP-seq peaks are relative to all coding genes and to NHR- 23-regulated genes, examining:
1. Within the gene body
2. Within promoter 1kb upstream from TSS
3. From 1kb to 3kb upstream of TSS
4. 3kb to 5kb upstream
These data are in Tables S2 and S5

2. Where? nhr-23 is expressed fairly broadly, and the authors don't address cell autonomy vs. non-autonomy or where nhr-23 acts to affect developmental progression or molting. This seems particularly important given some puzzling negative data with the degron allele. That data should probably be removed if the explanation can't be determined. Tissue-specific rescue experiments or mosaic analysis could get at the question.
>>> We have addressed where NHR-23 acts through tissue-specific RNAi (Fig. 8). These data suggest that nhr-23 is necessary in the seam or hypodermal cells for developmental progression, molting, and for BLI-1 expression.

3. How? The phenotypes seen after nhr-23 depletion are not examined in much detail, and although a variety of nhr-23 targets have been known for years (and some particularly good candidates are identified here), the authors do not demonstrate failure to express any of them to try to tie any target gene(s) to the different morphological phenotypes observed after nhr-23 knockdown. This makes different parts of the paper feel a bit disconnected or speculative and hampers a clear take home message. Examining some target genes after the different knockdown regimens could add a lot to the paper.
>>> We have addressed how NHR-23 acts through a nas-37 promoter reporter (Fig. 5) and BLI- 1, ROL-6, and NOAH-1 translational fusions (Fig. 6). NHR-23 depletion causes a reduction of nas-37 expression and a loss of reporter oscillation. NHR-23 depletion also causes aberrant localization of NOAH-1 and ROL-6 translational fusions and a barrier defect. NHR-23 depletion or knockdown causes reduction of BLI-1 expression and loss of BLI-1 localization to struts.

4. There are many places where n= information for images or Western blot replicates is missing from the legend, statistical tests are unexplained, or where data are described as "not shown". Specific examples are listed in the detailed comments below.
>>> We have addressed these points as described below.

Specific points (in order of appearance)

1. Lines 24-25 (Abstract): "Nematode molting is a remarkable process where the animals must

© 2023. Published by The Company of Biologists under the terms of the Creative Commons Attribution License (https://creativecommons.org/licenses/by/4.0/).

essentially build a new epidermis underneath the old skin and then rapidly shed the old skin."

This sentence is misleading and should be modified - the cuticle is an extracellular matrix distinct from the cellular epidermis - and I am not aware of any evidence that the cells in the epidermis must be re-built.
>>> We have re-worked this sentence to make it accurate.

2. The introduction raises many questions, most of which are not going to be addressed in this study, and the Discussion also is far-ranging. I would encourage the authors to more tightly refocus the text on the questions re: timing and targets that they actually tried to address here.
>>> We have re-worked the text to tighten it up and re-focus it as suggested.

3. Figure 1, Missing data details

Figure 1B - images are representative of HOW MANY animals examined at each timepoint? This info should be in the legend.
>>> We have added these data to the figure legend.

Figure 1C - these blots are representative of how many experimental replicates? This info should be in the legend (preferably) or in the Methods.
>>> These blots are representative of three experimental repeats and this info has been added to the legend.

4. Figure 2, Missing data details

Figure 2B - these blots are representative of how many experimental replicates?
>>> These blots are representative of three experimental repeats and this info has been added to the legend.

Figure 2D - Please provide some quantification of the arrest phenotype under the various regimens. Are all of the animals arrested after the early (2hr) AID shift?
>>> For the early depletion timepoints (2hr and 4 hr) the "arrested" animals were the animals that did not have an unshed cuticle (so 95% at hr and 72% at 4hr). We left the "arrest" phenotypic class out of the figure and discuss arrest vs severe developmental delay in the following paragraph.

5. Line 172: "molting defects were low (0-5%) when animals were shifted to auxin between 2-4 hours post release (Fig. 2D)." Does "2-4 hours" correspond to "2 hours" and/or "4 hours" in the figure? This is confusing because the numbers in text and figure don't seem to match.
>>> We have re-worked this text to match the data in Figure 2D.

6. Lines 175-176, related to Figure 2. "These data could suggest that NHR-23 may not be necessary for the initiation of the molt but required for completion of the molt."

It would be helpful if the authors were more explicit about how they define "molt initiation". Lethargus? Apolysis? Those are the classic criteria, but perhaps outdated. Expression of transient sheath components and early/furrow collagens begins in the middle of each larval stage, and perhaps this is the real beginning of the molt. This last idea is discussed in recent reviews (https://pubmed.ncbi.nlm.nih.gov/33036165/ and (https://pubmed.ncbi.nlm.nih.gov/33992153/).
>>> We have removed this line as much of the added data in revision is suggesting severe developmental delay rather than an arrest. At this point in the paper, we used more descriptive language and discuss more about the molting processes that might be defective later in the paper, once we introduce the translational fusions.

7. Related to Figures 2 and 3 and point 6. Not enough data are provided to know if arrest is due to failure to enter the molt in the first place, or if animals are stuck at an early step of the molt, before apolysis. There are behavioral and morphological features (e.g. activity levels, pharyngeal pumping, the buccal plug) and molecular markers (e.g. mlt-10::GFP::pest, others) that could distinguish these. (major point 3)

© 2023. Published by The Company of Biologists under the terms of the Creative Commons Attribution License (https://creativecommons.org/licenses/by/4.0/).

>>>This point is addressed by the above response.

The nlp-29::GFP result (Fig. 3) suggests to me that some amount of cuticle disassembly has begun in the arrested animals, which would mean that animals have initiated the molt, but it would be better to assess this more directly.

>>> Given the added Hoechst data (Fig 3) and ROL-6::mNG and BLI-1::mNG data, we feel that the nlp-29p::GFP reporter indicates a barrier defect, but animals are not arresting. Rather they are just progressing incredibly slowly through the larval stage and eventually attempt to molt.

8. Table 1 and Lines 196-201: "Only depletion using the vha-8p::TIR1 transgene was able to phenocopy the pan-somatic TIR1 allele (eftp::TIR1) (Table 1). These data could reflect: i) incomplete depletion of NHR-23 in hypodermis, intestine, neurons, or seam cells; ii) NHR-23 is not necessary in those cell types; iii) NHR-23 activity is required in multiple tissues, with single-tissue depletion being insufficient to disrupt molting."

No n= information is provided here and the data are not informative. Table could be removed or made supplemental.

This is where I suspect a degron approach might not be ideal for a nuclear protein. But the models and issues of cell autonomy vs. non-autonomy might be distinguishable using complementary tissue-specific rescue experiments or mosaic analysis. Otherwise, unfortunately there is not much we can conclude from the data. (major point 2)
>>> We have removed the tissue-specific auxin-inducible degron data and replaced it with tissue-specific RNAi data which suggest that NHR-23 activity is necessary in seam and/or hypodermal cells.

9. Figure 3, Missing data details

Figure 3A - images are representative of HOW MANY animals examined? What do the asterisks mean? Are the "early shift" animals the L2 arrested animals after 2hr shift? I'm not sure how this relates to Fig. 2.
>>> We have added the replicate info to the figure legend. The asterisks were a leftover from a draft where we had two types of auxin used (IAA and KNAA) are were to specify which auxin was used. As we only used IAA in this version, we have removed those asterisks and apologize for the confusion. The early shifted animals should be late L1 larvae based on the M cell data. We have tried to clarify this point in the revised manuscript.

Figure 3B - these blots are representative of how many experimental replicates?
>>> These blots are representative of two experimental replicates. This information has been added to the figure legend.

As mentioned above, activation of the cuticle damage sensor is consistent with idea that, under both "early" and "late" regimens, animals initiate the molt and then get stuck. More markers of the different steps in the molt cycle could get at what is happening. (major point 3)
>>> We have addressed this point with the additional transcriptional and translational reporter data.

10. Figure 4, Missing data details

Figure 4C - what do the asterisks mean and what statistical test was used for comparisons?
Figure 4E - images are representative of HOW MANY animals examined?
>>> We have added the statistical test information and number of animals we examined and for which the images in Fig 4E are representative. Note Fig 4C is now in supplemental

11. Related to Figure 4. Could developmental arrest be caused by occlusion of the buccal cavity so that the animals can't feed? The Frand lab has described a "plug" that forms there early in the molt (unfortunately unpublished). The Karp lab described a nice fluorescent bead assay for feeding: https://pubmed.ncbi.nlm.nih.gov/27172224/
>>> This suggestion was great and we tried it several times. There seemed to be quite a lot of

© 2023. Published by The Company of Biologists under the terms of the Creative Commons Attribution License (https://creativecommons.org/licenses/by/4.0/).

variability in the result. In several cases NHR-23-depleted animals were not feeding. In some repetitions they seemed to feed. Given the additional data we've added to strengthen the paper and that we are no suggesting developmental delay as opposed to an arrest, we are not including this data in the paper. I think that a careful study of when NHR-23-depleted animals stop feeding would be very interesting and something we might pursue for another study.
12. related to Figures 4 and 5 - The magnification is too low to see the vulva clearly, but even without nhr-23::AID, the TIR1 animals look abnormal, with advanced embryos inside. What's going on there?
>>> Good eyes, Reviewer 2! We've repeated the experiments with the TIR1 control strain from Figure 4 on auxin and ethanol several times, and not seen advanced embryos again (over 100 animals examined over 3 experiments). We've replaced in the image in Figure 4 (now Fig 3) and removed the daf-9 overexpression data from the revised manuscript, so the images from the original Fig 5 are no longer relevant.

13. Lines 263-265: What is mid late L4? (typo?)
>>>This line should have read late L4 and we have corrected this typo.

14. Figure 5 and Lines 274-315: "Larval arrest following NHR-23 depletion is independent of the insulin signaling pathway"

There is too much space dedicated to this negative result - it is the sort of thing that belongs in the supplemental material
>>>We completely agree. With the added data, we have drastically cut Fig 5 and moved its content into the supplemental material

Figure 5A - what do the asterisks mean and what statistical test was used for comparisons?
>>>We used a Student's two-tailed T-test and added this information to the figure legend.

15. Figure 6 and Table S1 (re-analysis/synthesis of published RNA profiling and ChIP data) contain some of the most interesting data in the paper, but presentation is not as useful as it could be.

Fig. 6B ChIP analysis would be more valuable if confidently-bound target genes were plotted as a comparison to Fig. 6C or integrated into the annotation in Table S1 (major point 1)
>>>We had added this data to Table S2 (formally Table S1) and added Table S5 which contains our mapping data to all genes.

How do the nhr-23-enriched GO-terms differ from those of oscillating genes as a whole? (a large proportion of oscillating genes are matrix-related, but are you seeing anything particularly unique in nhr-23-enriched vs. non-enriched oscillating genes?)
>>>This is a great point. We've performed this analysis and provided it as a supplementary table and also replaced the original NHR-23-regulated oscillating gene GO analysis with a table. We used "function" instead of "process" in this version of the GO analysis and NHR-23- regulated genes seem to be specifically enriched in structural components of the cuticle and in regulation of protease activity. We build on this observation in the revised discussion

For Fig. 6C, consider subdividing "aECM components" to tie into the emerging biology of assembly/disassembly. e.g., Where do transient sheath factors, furrow collagens, and other classic "early" vs "late" cuticle collagens show up here?
>>>We have created a new Figure with a subset of aECM components with subdivided function which is now Fig 4E.

Finally, could the authors look at some representative targets from these classes (e.g. with existing reporter fusions) to more precisely determine where the block is in their different phenotypic classes of nhr-23 knockdown animals? (major point 3)
>>>We used a nas-37 promoter reporter and added NOAH-1, BLI-1, and ROL-6 translational fusions.
16. Fig. 6B, modENCODE should be cited in main text and legend as the source of the ChIPseq data. The prior text sentence seems to cite modENCODE for oscillation, not for ChIPseq.
>>> We have corrected this issue.

© 2023. Published by The Company of Biologists under the terms of the Creative Commons Attribution License (https://creativecommons.org/licenses/by/4.0/).

17. Lines 334-336. "There are also several sets of genes involved in apical extracellular matrix biogenesis and remodeling, such as lipocalins, proteases, protease inhibitors, fibrillin, PAN and ZP domain-containing proteins, and leucine-rich repeat proteins. "

This is the first mention of most of these proteins/domains, so acronyms should be defined. It would be helpful to provide references or other information to support the statement that these genes/proteins are involved in matrix biogenesis, or to help reader understand how they relate to cuticle (most are not actually part of the cuticle, they are associated with the transient sheath).
>>>We appreciate this point and have re-worked the introduction to provide this information.

18. Table S1: would be much more useful if ChIP data were also annotated here. (major point 1)
>>>We have added this data to Table S2 (formally Table S1) and provided more nuanced data in Table S5.

19. There are 4 places in the manuscript where "unpublished data" are cited. All data should be either shown or not mentioned.
>>> We have corrected this issue and removed all mentions of unpublished data.

20. Figure S1: DIC and DIC+GFP labels are reversed
>>> We have corrected this issue.

21. Figure S3: It would be useful if definitions for "early", "mid", and "late" L4 were included in this legend rather than referring to Fig. 4.
>>> We have adjusted the Figure S3 legend accordingly.

22. Figure S4: The data in Figure 5 could go here.
>>> We have removed the original Figure S4 and moved the data in the original Figure 5 into supplemental (now Fig S4).

23. Figure S8 and Table S2 feel quite peripheral to this story
>>> We agree, especially given the data we have added. We have removed this figure and Tables S2 and S3 the discussion related to them.

24. line 874 space missing
>>> We have corrected this issue.

Reviewer 3 Comments for the Author:
Major comments

Figure 1. Number of animals examined for showing oscillation of NHR-23::GFP is not indicated. Biological and technical replicated should be indicated. How many animals at a certain time point show oscillations? These numbers would boost confidence in the data.
>>> We have added the number of biological replicates, animals scored, and the number of animals at which timepoint for which the images are representative to the figure and legend.

Table 1 fails to mention number of experiments, population size, faction of animals with phenotypes etc.
>>> As reviewer 2 also noted some limitations in claims that the data in Table 1 supports. We have replaced the data in Table 1 with more robust tissue-specific RNAi data.

Depletion of NHR-23 leads to a defective cuticle barrier: I disagree with the use of Pnlp-29::GFP as a reporter for cuticle barrier dysfunction. This reporter can not be used as proxy for barrier function defect. NLP-29 is believed to be an antimicrobial factor, induced in response to various assaults to the epidermis including including infection, chemicals and physical injury. It responds both to chronic damage as well as acute stress. Breach of this barrier is unlikely to have happened during osmotic stress or tunicamycin treatment. Authors must use a direct measure for barrier function such as Hoechst stain permeability, AFM or SEM analysis of the cuticle.
>>> We appreciate this point and have added Hoechst staining to the figure and removed the nlp-29 data. NHR-23 depletion caused robust Hoechst staining which, along with the aECM

© 2023. Published by The Company of Biologists under the terms of the Creative Commons Attribution License (https://creativecommons.org/licenses/by/4.0/).

defects in ROL-6 and BLI-1 localization, supports a barrier defect.

Authors show correlation between the wave of NHR-23 protein expression and molting (Figure 6). To conclude that NHR-23 oscillation is responsible for aECM formation at each molt, authors must disrupt the oscillations of NHR-23 and maintain it at high levels. This can be done by keeping NHR-23 ON under a non oscillating promoter such as vha-8p. If authors hypothesis is correct, keeping NHR-23 ON should lead to loss of oscillation in aECM components as well.
This experiment is absolutely essential for this study.Authors should make use transcript and reporter (for aECM components) analysis for these assays.
>>> This experimental suggestion is fantastic but is proving challenging. We tried to use heat-shock promoters to induce NHR-23 expression. We were never able to get lines from extrachromosomal arrays. We managed to generate lines using single-copy integration and it seems like overexpression of NHR-23 can drive a nas-37 promoter reporter. However, reporter activity is also increased in the absence of heat-shock potentially due to leaky expression, complicating the results. NHR-23 depleted animals with the reporter might be getting further in development, but this needs more careful examination. As we re-worked the manuscript this experiment did not seem to fit the new flow and seems worthy of a separate, deep analysis of whether NHR-23 oscillation is required for developmental progression, molting, aECM remodeling, and oscillating target expression. We hope that the Reviewer feels that the extensive added data (demonstrating developmental delay, nas-37 promoter reporter, Hoechst staining, translational fusions and tissue-specific RNAi) have shifted the message of the paper towards when and where NHR-23 acts and how it promotes aECM regeneration.

Other Comments
NHR-23 is required in hypodermis, intestine, neurons, or seam cells. This should be discussed. Are authors invoking non cell autonomous events and communication between these tissues. When does this happen?

Scale bar in Figure 1 should be mentioned in the legend.
>>>This information has been added to the legend.

Is the oscillation of NHR-23 dependent on oscillation of BLMP-1 expression or function?
>>>This question is a fantastic one and certainly one for future exploration. Given BLMP-1's role as a pioneer factor, it would be intriguing to see if nhr-23 was regulated by BLMP-1. However, we have not done these experiments as we aware of two other labs actively pursuing this line of experimentation in depth and we do not wish to scoop them.

Line 51: This group of pathogens infect over one billion humans and devastate crops and livestock, yet few drugs exist to counter them and resistance is emerging (Ward, 2015b). This can be split and reworded, for example to ''parasitic nematodes cause over a billion human infections each year and loss of livestock and crops. However, very few drugs exist and resistance to those drugs is emerging rapidly.
>>>We have adjusted the text as suggested.

In Auxin exposure regime, animals were maintained on auxin until imaging. Why aren't the abnormalities seen in that case even on continuous exposure to auxin? Later it is mentioned that animals with this much exposure show developmental arrest. Rescue experiments by removing the animals from auxin after early exposure and late exposure to confirm stage specific effects.
>>>We have addressed this point with additional data. We found that it took multiple days for animals to attempt to molt, at which point we see similar defects as in the late auxin shift experiments.

Data in Fig 2D can be better represented as a histogram.
>>>We appreciate this comment and have tried multiple histograms and more graphical approaches to represent this data over the life of this project. In each case the figure was difficult to interpret as it involved two strains with two treatment regiments and the take-home message was not intuitive. The table format has consistently been the most effective way to convey the data. Given this data and our attempts to represent the data in the histogram format, we hope that the reviewer will find leaving the table in Fig 2D to be acceptable.

© 2023. Published by The Company of Biologists under the terms of the Creative Commons Attribution License (https://creativecommons.org/licenses/by/4.0/).

The significance is shown in terms of CI and sometimes as p value. It is confusing. Representation as p-value will help in easy interpretation.
>>>We have removed Cis and replaced them with p-values.

Resubmission

First decision letter

MS ID#: DEVELOP/2022/201085

MS TITLE: NHR-23 activity is necessary for developmental progression and apical extracellular matrix structure and function

AUTHORS: Londen C. Johnson, An A. Vo, John C. Clancy, Joseph Aguilera, Max T. Levenson, Chloe Wohlenberg, Andreas Rechtsteiner, James Matthew Ragle, and Jordan D. Ward

I have now received all the referees' reports on the above manuscript, and have reached a decision. As you will see, the referees think that the revised manuscript had substantial work which improves the study. However, the reviewers also suggest recommendations to significantly clarify the manuscript textually, since a lot of the writing in currently dense, which makes the flow of logic difficult to follow. In addition, at several places in the manuscript the text please caveat the findings, when the data is not strong enough to support the conclusion. For example, the conclusion that nrh-23 functions in the seam cells to regulate molting is not entirely supported by the data presented. Thus, this conclusion will need to be softened in the manuscript. The reviewers provide several similar suggestions for improving clarity and scholarship in the manuscript. If you are able to revise the manuscript along the lines suggested, which may involve further experiments, I will be happy to receive a revised version of the manuscript. Your revised paper will be re-reviewed by one or more of the original referees. Please also note that Development will only allow one more round of major revision.

Please attend to all of the reviewers' comments and ensure that you clearly highlight all changes made in the revised manuscript. Please avoid using 'Tracked changes' in Word files as these are lost in PDF conversion. I should be grateful if you would also provide a point-by-point response detailing how you have dealt with the points raised by the reviewers in the 'Response to Reviewers' box. If you do not agree with any of their criticisms or suggestions please explain clearly why this is so.

Reviewer 1

Advance summary and potential significance to field

The authors responded to the prior review comments by providing a substantial amount of new data, including more careful analysis of nhr-23 mutant phenotypes with expression analysis for multiple potential targets, tissue-specific RNAi experiments to address site of action, and more detailed cross-annotation of the published RNA profiling and ChIP data. The manuscript is substantially improved from the original.

I don't think that the new data address the main concern of Reviewer 3 about whether NHR-23 specifically drives target oscillation, nor do they really change my "big picture" view re the role of NHR-23. But they do add some nice tools and pieces to the puzzle including likely direct targets, show clear effects on target expression levels and matrix organization, and hint at a special role for the seam in molt cycle progression.

Comments for the author

I have a number of questions and suggestions to improve aspects of the presentation that I found difficult to understand.

© 2023. Published by The Company of Biologists under the terms of the Creative Commons Attribution License (https://creativecommons.org/licenses/by/4.0/).

Major comments:

1. Re "when" and the nhr-23 depletion experiments in Figs. 2, S4, and 3: Please be more explicit in the text about what is a new conclusion beyond that in MacNeil et al, 2013, who concluded that NHR-23 affects the rate of developmental progression.

2. Re "where" and interpretation of the tissue-specific RNAi expts in Fig. 8: The Abstract overstates that "nhr-23 acts primarily in seam cells", whereas the main text is more careful to say "seam and hypodermal cells". It would actually be quite interesting if nhr-23 was acting in seam cells to control developmental progression and matrix remodeling in the whole body, but I don't think the data are that clear yet.

Also, the method for tissue-specific RNAi should be better described; the current Methods text doesn't actually say that the RNAi is tissue specific and neither the Results or Methods describe the rde-1 rescue strategy.

3. Re "how" and annotation of nhr-23 targets: The added data regarding nas-37 and collagens nicely demonstrate expression changes in some predicted targets. The revised Table S2 and Figure 4D are also useful for getting a handle on likely targets, but in Fig 4D it's pretty confusing to see the "late" collagens peaking earlier than the "early" or "intermediate" collagens. Is that correct?? An explanatory note in the legend would be helpful, e.g. if you think that the original "late collagen" nomenclature was wrong.

Also perhaps related, Table S2 legend says: "Meeuse et al. 2021 and Hendriks et al. 2015 datasets had a phase difference of ~160° which puts the molt in the Meeuse dataset between 340° and 70°. We set the molt to 0°". What do you actually mean by molt, and how can it be so precisely defined in your phasing whereas it occurs over several hours in the original dataset phasing? Could some artifact of setting a new phase/hour relationship explain how late collagens got put in the "early" hour bin?

4. Collagens and other knock-ins: Several fusions are newly described here and they are written as though tags are at extreme C-terminus. Is this really the case? If not, please clarify in text or add cartoons. Tags at the C-term could be mis-representative since matrix proteins often get their C-terms cleaved off before matrix incorporation.

5. The writing could be streamlined, especially in the Results sections about the degron allele. Remember that you are under no obligation to present your narrative historically, with all its confusing twists and turns. Prioritize clarity of the take-home messages! Furthermore, data details such as specific %s of animals don't need to be listed out in the text if shown in the figures.

6. In general, I don't find that take home messages are easy to glean from the figures. Better labels could help with this. The numbers on the figures can be confusing (e.g. when both control and expt say 40/40, but the numerators are referring to different properties and the results are different). Use of strain names as graph labels is meaningless to most of us and requires cross-referencing with a table to understand.

Detailed comments (in order of appearance):

7. Figure 1A: Please cite basis for showing L1-L2 lethargus (purple) starting at ~8 hours. It would be helpful if this plot included the hours used for experiments in the rest of the figure (e.g. Molt 1 at 16 hours).

8. line 74: "thought to be secreted by the excretory gland". There is no need to repeat this speculation, which has propagated through the literature despite the fact that it is not supported by any data in the cited papers.

9. line 162: It would be good to define "post-release" in the results if you are going to use this term throughout. I'm sure you mean after release from L1 starvation arrest used for synchronization, but the results don't actually allude to this method.

© 2023. Published by The Company of Biologists under the terms of the Creative Commons Attribution License (https://creativecommons.org/licenses/by/4.0/).

10. Figure 2 and lines 145-184. This way of presenting the data is very confusing, because it gives the impression that most of the earliest shifted animals are paradoxically normal. There is nothing in the figure or text paragraph to say otherwise. In fact, Figure 2A visually implies that those animals have progressed past Molt 1. Please restore %arrest (or %arrest/delay) to 2D and tell us outright in the text that the early shifted animals were still small and appeared delayed! Or even better, why not condense this section and merge with the next (NHR-23 depletion causes severe developmental delay) to get right to the point?

11. lines 180-182 "We repeated the 16 hour shift experiment and scored animals 48 hours later. Based on size, gonadal morphology, and vulval morphology, animals were L2 and L3 larvae (Figure S3)." This sentence feels tacked on and not explained, and it raises many questions. First, the sentence is only interpretable if compared to controls (which were mostly adult?). Second, does this result mean that nhr-23 is not required for L2 progression or the L2-to-L3 molt? Or is the freshly made NHR-23 in L2 not getting degraded?

12. Figure 3C and 3E graphs show experimental data without paired control data.

13. Figure3D: "Adult with oocytes" has lots of embryos inside too, but its normal for adults to have oocytes and embryos. The point is not coming through.

14. Figure 4B legend: the word "late" is missing after 240°-360°.

15. Figure 4C: It appears that some non-Osc genes have lots of NHR-23 peaks - Thoughts? I didn't see this discussed.

16. Figures 4C vs 4D: The hours on the X-axis of these two graphs both run from 0-9 but don't mean the same thing, which is a little confusing - why not keep them consistent?

17. Figure S6 and lines 305-308: "In noah-1::mNG::3xFLAG, nhr-23::AID; TIR1 lysates there was a band of the expected size (~150 kDa) as well as a lower ~50 kDa band. noah-1 is only predicted to have a single isoform so this might reflect a cleavage or degradation product." Please clarify where the tag is. If the mNG tag is at the C-terminus, then you would expect a band just larger than mNG due to cleavage of NOAH-1 after its ZP domain (as happens with almost all ZP proteins) and you might not actually be assessing matrix with your fusion.

18. Figure 6B and lines 314-315: How can you tell if ROL-6 is labelling annuli vs. furrows?

19. Figure 6G and lines 338-339: How can you tell those are lysosomes?

20. Figure 7A: These data could be shown with fewer photos and a bar graph for quantitation. The auto-fluorescence in the body images is distracting and gives the impression that controls have more staining than the experimental worms.

21. Figure 7B: no n= info here

22. Figure 7D: % surviving animals under what regimen?

23. Figure 8A: In addition to the spatial expression of these promoters, the temporal expression is no doubt important for RNAi efficiency (at least some of them are oscillatory and/or are predicted nhr-23 targets) - it would be helpful to indicate that information too. Also, please define SCMp, which is non-standard nomenclature - this is your most effective driver and people should be able to understand what it is.

24. Figure 8C: Is there a label missing from X axis?

25. Figure 8D: The control and expt images appear to be completely different body regions and focal planes. "L4440(RNAi)" = "Control RNAi" elsewhere, be consistent.

© 2023. Published by The Company of Biologists under the terms of the Creative Commons Attribution License (https://creativecommons.org/licenses/by/4.0/).

26. line 412: "a set of pre-cuticle, early collagen, protease inhibitor genes" missing word "and"?

27. Line 448: " When we depleted NHR-23 early in L1, animals appeared wild type". But aren't they delayed in progression and still in L1?? Rephrase this, it contributes to the overall confusion surrounding the depletion experiments.

28. Table S3: Why are there 3 columns of P values, what are they? The last column doesn't look like P values. The table key is not clearly connected to what is shown. Overall this is very confusing.
29. References: Meeuse 2022 reference appears incomplete (biorxiv)

Reviewer 2

Advance summary and potential significance to field

This manuscript by Johnson et al. describes significant advancements in understanding the function of oscillating expression of the nuclear hormone receptor NHR-23 in C. elegans larval development. NHR-23 was previously shown to be essential for embryonic development and larval molting, but the temporal requirement for NHR-23 was not explored. Here the authors provide compelling evidence that pulses of NHR-23 are necessary for molting and progression to the next larval stage (from my previous review).

The revised manuscript by Johnson et al. is much improved. The authors address my major concern of the original submission regarding the effects of continuous auxin-mediated depletion of NHR-23 during the L1-stage using western blots to demonstrate that NHR-23 levels remain low throughout the L1-stage in auxin treated animals (Figure 2B), and they add some appropriate speculation regarding why larvae depleted of NHR-23 in early L1 stage exhibit less severe molting defects than those treated in later in the L1 stage. They also carefully addressed each of my more minor concerns. This revised version is more clearly written and much easier to read.

Comments for the author

Thanks for the careful revisions, and your point-by-point response to the earlier reviews.

Reviewer 3

Advance summary and potential significance to field

How extracellular matrices are assembled remains poorly understood. C. elegans serves as an excellent model to explore the mechanism by which apical extracellular matrices (aECMs) are assembled, because the nematode repeatedly reforms aECMs during every molt and their integrity can be easily visualized by fluorescently-tagged proteins. Taking advantage of these features, this paper shows that the nuclear hormone receptor NHR-23/NR1F1 promotes appropriate assembly of components of aECMs by coordinating expression of genes involved in molting, lipid transport/metabolism and aECM components. This study suggests that strictly timed transcription of aECM components plays a key role in robust assembly of aECMs.

Comments for the author

The authors have appropriately provided point-by-point responses to comments previously raised by reviewers. I have only minor comments below.

Minor Comments:
1) Line 210: This paragraph starts with “To distinguish between larval arrest and developmental delay,..” and eventually ends by stating “these data suggest that NHR-23 depletion causes developmental delay and 233 animals die due to a failed molt.”

Does the last sentence claim that depletion of NHR-23 does NOT cause larval arrest? "wild type" in Fig 3EF is confusing; The size of the “wild type” animal in Fig 3F suggests that the “wild type” animals in Fig 3E are all L1 arrested animals, which are easily recognized by the L1 alae, instead of normally growing larvae/adults. If one would not see any progression of the event of interest during

© 2023. Published by The Company of Biologists under the terms of the Creative Commons Attribution License (https://creativecommons.org/licenses/by/4.0/).

the defined time window though the penetrance is low, I believe one should mention that the event is “arrested (at least during XX hours)” instead of “delayed”.
2) Related to the above, Fig S4 and Fig 3EF can be integrated. Auxin exposure at hour 0 as described for Fig 3EF may allow the authors to see the earliest arrest point of M lineage in the “wild type” fraction instead of variable arrest shown in Fig S4C. That arrest point would be the rate-limiting, developmental step regulated by nhr-23, and may overlap with the time window during which the reporter is detectable (Fig1 B).

3) Line 212: “We performed depletion experiments shifting early L3 animals onto auxin and monitored development by scoring vulva morphology 24 hours later.”
Line 1090: “Animals of the indicated genotype were synchronized and shifted onto auxin at 25 hours post-release and imaged 23 hours later (48 hours post-release).”

The main text states the phenotype was scored 24 hours after auxin administration. However, the legend says 23 hours later. Which is true?

4) Line 261: Descriptions of Fig 4A, B appear after those of Fig 4D, E. The order of the figures should be changed to avoid confusion.

5) Line 277 and Fig 4C: What is the “NHR-23 peaks”? In Fig 4C, the NHR-23 peaks for the x axis seems to mean peaks in expression level. But I do not understand what NHR-23 peaks means for y axis.

6) Fig 4E was not labelled.

7) Line 281 and Figure 5: At what developmental time point were these animals “released” onto the auxin plate?

8) Fig 6 legend: Explain the numbers on the upper left corner of photos.

9) Line 1098: Were the pics of animals taken 48 or 72 hours after the auxin exposure?

First revision

<u>Author response to reviewers' comments</u>

>>> We thank the reviewers for their helpful and constructive suggestions, which drastically improved this manuscript. We have provided a point-by-point response to their comments below. We hope that these revisions address the previous concern of the reviewers.

Reviewer 1 Advance Summary and Potential Significance to Field:
The authors responded to the prior review comments by providing a substantial amount of new data, including more careful analysis of nhr-23 mutant phenotypes with expression analysis for multiple potential targets, tissue-specific RNAi experiments to address site of action, and more detailed cross-annotation of the published RNA profiling and ChIP data. The manuscript is substantially improved from the original.

I don't think that the new data address the main concern of Reviewer 3 about whether NHR-23 specifically drives target oscillation, nor do they really change my "big picture" view re the role of NHR-23. But they do add some nice tools and pieces to the puzzle including likely direct targets, show clear effects on target expression levels and matrix organization, and hint at a special role for the seam in molt cycle progression.

>>>We acknowledge that the last revision did not address Reviewer 3’s point about whether NHR-23 drives target gene oscillation. Below we have provided our response to Reviewer 3 for the last submission, which satisfied their concern. Addressing NHR-23 oscillation in relation to target gene oscillation is challenging and will likely require a separate manuscript to address in depth.

© 2023. Published by The Company of Biologists under the terms of the Creative Commons Attribution License (https://creativecommons.org/licenses/by/4.0/).

Previous response
"This experimental suggestion is fantastic but is proving challenging. We tried to use heat-shock promoters to induce NHR-23 expression. We were never able to get lines from extrachromosomal arrays. We managed to generate lines using single-copy integration and it seems like overexpression of NHR-23 can drive a nas-37 promoter reporter.
However, reporter activity is also increased in the absence of heat-shock potentially due to leaky expression, complicating the results. NHR-23 depleted animals with the reporter might be getting further in development, but this needs more careful examination. As we re-worked the manuscript this experiment did not seem to fit the new flow and seems worthy of a separate, deep analysis of whether NHR-23 oscillation is required for developmental progression, molting, aECM remodeling, and oscillating target expression. We hope that the Reviewer feels that the extensive added data (demonstrating developmental delay, nas-37 promoter reporter, Hoechst staining, translational fusions and tissue-specific RNAi) have shifted the message of the paper towards when and where NHR-23 acts and how it promotes aECM regeneration."

Reviewer 1 Comments for the Author:
I have a number of questions and suggestions to improve aspects of the presentation that I found difficult to understand.

Major comments:

1. Re "when" and the nhr-23 depletion experiments in Figs. 2, S4, and 3: Please be more explicit in the text about what is a new conclusion beyond that in MacNeil et al, 2013, who concluded that NHR-23 affects the rate of developmental progression.
>>>We have clarified the text to highlight which aspects of our data reproduce observations from MacNeil et al., 2013 and Patel et al., 2022.

2. Re "where" and interpretation of the tissue-specific RNAi expts in Fig. 8: The Abstract overstates that "nhr-23 acts primarily in seam cells", whereas the main text is more careful to say "seam and hypodermal cells". It would actually be quite interesting if nhr-23 was acting in seam cells to control developmental progression and matrix remodeling in the whole body, but I don't think the data are that clear yet.

Also, the method for tissue-specific RNAi should be better described; the current Methods text doesn't actually say that the RNAi is tissue specific and neither the Results or Methods describe the rde-1 rescue strategy.
>>>We have corrected the oversight in the abstract and aligned it with the main text to be clear that our data support roles for nhr-23 in hyp and seam cells. We have added text to the Methods describing the tissue-specific RNAi.

3. Re "how" and annotation of nhr-23 targets: The added data regarding nas-37 and collagens nicely demonstrate expression changes in some predicted targets. The revised Table S2 and Figure 4D are also useful for getting a handle on likely targets, but in Fig 4D it's pretty confusing to see the "late" collagens peaking earlier than the "early" or "intermediate" collagens. Is that correct?? An explanatory note in the legend would be helpful, e.g. if you think that the original "late collagen" nomenclature was wrong.

Also perhaps related, Table S2 legend says: "Meeuse et al. 2021 and Hendriks et al. 2015 datasets had a phase difference of ~160° which puts the molt in the Meeuse dataset between 340° and 70°. We set the molt to 0°". What do you actually mean by molt, and how can it be so precisely defined in your phasing whereas it occurs over several hours in the original dataset phasing? Could some artifact of setting a new phase/hour relationship explain how late collagens got put in the "early" hour bin?
>>>We reached out to the Grosshans lab to clarify their meaning of molt in the datasets. In the original dataset from Hendriks et al. they observed animals in a timecourse and defined molt as the onset of lethargus to the end of ecdysis. They then correlated the gene expression data in Meeuse et al. to find an equivalent gene expression signature. They have recently refined this estimate (now published in Meeuse et al., 2023) with 45° being the onset of lethargus and 135° being the end of ecdysis. This difference between how we set our initial phasing and when ecdysis likely occurs explains why a late collagen was depicted early in a larval stage. *ram-2* (classified as

© 2023. Published by The Company of Biologists under the terms of the Creative Commons Attribution License (https://creativecommons.org/licenses/by/4.0/).

a late collagen) now comes up later in the molt, as opposed to at the beginning. We've clarified the figure legend and methods to note exactly how the molt was classified. This new estimate of when the molt occurs also agrees with our NHR-23 expression data.

4. Collagens and other knock-ins: Several fusions are newly described here and they are written as though tags are at extreme C-terminus. Is this really the case? If not, please clarify in text or add cartoons. Tags at the C-term could be mis-representative since matrix proteins often get their C-terms cleaved off before matrix incorporation.
>>>We apologize for the lack of clarity on these strains. rol-6::mNG::3xFLAG is knock-in at the extreme C-terminus, while the noah-1 and bli-1 knock-ins are internal. We have added schematics to Figures 5 and 6 to clarify where the knock-ins are. We have also adjusted our strain genotype nomenclature adding an "(int)" at the end for internal tags, similar to Vuong-Brender et al., to make the nature of the insertion clearer. Since, as the reviewer notes, several of these fusions are new we've shifted the western blots to the main figure and added L4 expression timecourses to characterize expression of these new fusions.

5. The writing could be streamlined, especially in the Results sections about the degron allele. Remember that you are under no obligation to present your narrative historically, with all its confusing twists and turns. Prioritize clarity of the take-home messages! Furthermore, data details such as specific %s of animals don't need to be listed out in the text if shown in the figures.
>>>We have cut a lot of detail, particularly for the timed depletion experiments and removed data details where appropriate. We hope that these changes help clarify take home messages.

6. In general, I don't find that take home messages are easy to glean from the figures. Better labels could help with this. The numbers on the figures can be confusing (e.g. when both control and expt say 40/40, but the numerators are referring to different properties and the results are different). Use of strain names as graph labels is meaningless to most of us and requires cross-referencing with a table to understand.
>>>We have worked to clarify the figures and move the numbering that was confusing into the legends where it can be more clearly explained. We removed strain names from graphs. This latter point was likely specific to Fig 8 and we have presented that data as a table in the revised version.

Detailed comments (in order of appearance):

7. Figure 1A: Please cite basis for showing L1-L2 lethargus (purple) starting at ~8 hours. It would be helpful if this plot included the hours used for experiments in the rest of the figure (e.g. Molt 1 at 16 hours).
>>>We have removed Figure 1A based on conversations with the Grosshans lab. As mentioned in point 3, they provided a revised version of where lethargus and molting are occurring in their RNA-seq timecourse, which shifted the nhr-23 mRNA peak earlier in development. This peak is earlier than that described in the Gissendanner paper which we note. However, the Grosshans data do agree with our GFP images and western blotting, where NHR-23 protein appears to peak earlier in larval stages. These data also agree with a recent preprint from the Hammell lab, using an nhr-23::mScarlet strain that we developed. We confirmed that NHR-23 peaks early in L4 with new data (Fig 1D,E). I dug into our initial molting estimates in Figure 1 from the past submissions and my student based those estimates on published developmental timing tables. During revisions, we performed timecourse analysis with these strains to score lethargus. Our strains were entering lethargus later than the commonly used table from Byerly et al., 1976. KRY88 has a slight developmental delay, but even our WT strain is entering lethargus later. The source of this variation is unclear, it's possible that our MYOB plates or some variation in our lab conditions or media is causing this developmental rate. We have clearly stated these points in the paper.

8. line 74: "thought to be secreted by the excretory gland". There is no need to repeat this speculation, which has propagated through the literature despite the fact that it is not supported by any data in the cited papers.
>>>We have removed this statement and appreciate the reviewer highlighting the issue with it being propagated through the literature.

© 2023. Published by The Company of Biologists under the terms of the Creative Commons Attribution License (https://creativecommons.org/licenses/by/4.0/).

9. line 162: It would be good to define "post-release" in the results if you are going to use this term throughout. I'm sure you mean after release from L1 starvation arrest used for synchronization, but the results don't actually allude to this method.
>>>The reviewer is correct about what we meant by “post-release” and we have now clarified this point in the text.

10. Figure 2 and lines 145-184. This way of presenting the data is very confusing, because it gives the impression that most of the earliest shifted animals are paradoxically normal. There is nothing in the figure or text paragraph to say otherwise. In fact, Figure 2A visually implies that those animals have progressed past Molt 1. Please restore %arrest (or %arrest/delay) to 2D and tell us outright in the text that the early shifted animals were still small and appeared delayed! Or even better, why not condense this section and merge with the next (NHR-23 depletion causes severe developmental delay) to get right to the point?
>>>We have streamlined this section by removing the original Figure S3 and combined Figures 2 and 3 under the heading of “NHR-23 depletion causes severe developmental delay”. We condensed the sections covering those figures, as the reviewer suggested.

11. lines 180-182 "We repeated the 16 hour shift experiment and scored animals 48 hours later. Based on size, gonadal morphology, and vulval morphology, animals were L2 and L3 larvae (Figure S3)." This sentence feels tacked on and not explained, and it raises many questions. First, the sentence is only interpretable if compared to controls (which were mostly adult?). Second, does this result mean that nhr-23 is not required for L2 progression or the L2-to- L3 molt? Or is the freshly made NHR-23 in L2 not getting degraded?
>>>We have removed this text and Figure S3. What we think was occurring was that we had scored the animals 48 hours after the 16 hour auxin shift. Based on our more recent results suggesting that NHR-23 depletion caused severe developmental delay, animals made it through the L1 molt as there was no NHR-23 to deplete since we were past the peak of expression. NHR-23 was then degraded in L2 larvae which resulted in some animals remaining in L2. But as we saw in our L4 depletion experiments, animals can continue to develop and attempt to molt, thus some animals may have reached L3. I think that the point is made more cleanly in the L3/L4 depletion dataset as we can more precisely stage the animals with vulval development. The L2 molt may be worth more careful future study as work from the Fay lab on the nekl-2 and nekl-3 kinases (Binti et al., 2022) indicates that there is something different about the L2d decision point, so it’s possible that the role of NHR-23 in this molt may differ.

12. Figure 3C and 3E graphs show experimental data without paired control data.
>>>Paired control data has been added to these graphs and they are now in Figure 2 (Fig 2G and E, respectively).

13. Figure3D: "Adult with oocytes" has lots of embryos inside too, but its normal for adults to have oocytes and embryos. The point is not coming through.
>>>These images have been moved to supplement and clarified to make the point that some of the *nhr-23::AID; TIR1* animals grown on auxin for 72 hours have reached adulthood.

14. Figure 4B legend: the word "late" is missing after 240˚-360˚.
>>>We have added this word to the legend. With the revised timing of lethargus and ecdysis from the Grosshans lab, we have adjusted the ranges classified as early, middle, and late.

15. Figure 4C: It appears that some non-Osc genes have lots of NHR-23 peaks - Thoughts? I didn't see this discussed.
>>>Great point! We have added few sentences about this observation to the Discussion.

16. Figures 4C vs 4D: The hours on the X-axis of these two graphs both run from 0-9 but don't mean the same thing, which is a little confusing - why not keep them consistent?
>>>We apologize for the confusion. We’d initially set the nhr-23 mRNA peak as our 0 in Fig 4D and then plotted the number of NHR-23 ChIP-seq peaks (flanking and within each regulated gene) in 1 hr bins after the peak. The goal was to show that there was a trend of more NHR-23 peaks in target genes closer to the nhr-23 mRNA peak. However, we recognize that the difference in X-axes between Figures 4C and 4D was confusing and have adjusted the 4D axis to match the 4C axis. There is still a clear trend of more NHR-23 ChIP-seq peaks in and surrounding genes that

© 2023. Published by The Company of Biologists under the terms of the Creative Commons Attribution License (https://creativecommons.org/licenses/by/4.0/).

peak in expression close to the nhr-23 mRNA peak in expression. Note these data are now in Fig 3.

17. Figure S6 and lines 305-308: "In noah-1::mNG::3xFLAG, nhr-23::AID; TIR1 lysates there was a band of the expected size (~150 kDa) as well as a lower ~50 kDa band. noah-1 is only predicted to have a single isoform so this might reflect a cleavage or degradation product." Please clarify where the tag is. If the mNG tag is at the C-terminus, then you would expect a band just larger than mNG due to cleavage of NOAH-1 after its ZP domain (as happens with almost all ZP proteins) and you might not actually be assessing matrix with your fusion.
>>>We appreciate the reviewer bringing the cleavage after the ZP domain in almost all ZP proteins to our attention. Our knock-in is right after the ZP domain. We aimed to insert the mNeonGreen::3xFLAG cassette in roughly the same location as the mCherry knock-in generated by Vuong-Brender et al. Though the repair oligos and crRNA wasn't described in that papers, we reached out to the Labouesse lab and our knock-in is in essentially the same position.

18. Figure 6B and lines 314-315: How can you tell if ROL-6 is labelling annuli vs. furrows?
>>>We have added expression timecourses for NOAH-1, ROL-6, and BLI-1 translational fusions. We adjusted our language to indicate that the ROL-6 localization pattern resembles furrows initially (narrow bands) and then redistributes to a pattern resembling annuli (thick bands).

19. Figure 6G and lines 338-339: How can you tell those are lysosomes?
>>>We initially had made the claim based on morphology as we'd colocalized MLT-11 to lysosomes in a preprint using a SCAV-3::GFP marker. To strengthen the assertion that NOAH-1 is in lysosomes, we've added colocalization data between NOAH-1::mNG and a NUC-1::mCherry translational fusion. We have also provided a Manders' co- efficient to analyze the extent of co-localization.

20. Figure 7A: These data could be shown with fewer photos and a bar graph for quantitation. The auto-fluorescence in the body images is distracting and gives the impression that controls have more staining than the experimental worms.
>>>We have removed the photos and simply provided a bar graph.

21. Figure 7B: no n= info here
>>>We have added this information.

22. Figure 7D: % surviving animals under what regimen?
>>>We have added this information.

23. Figure 8A: In addition to the spatial expression of these promoters, the temporal expression is no doubt important for RNAi efficiency (at least some of them are oscillatory and/or are predicted nhr-23 targets) - it would be helpful to indicate that information too. Also, please define SCMp, which is non-standard nomenclature - this is your most effective driver and people should be able to understand what it is.
>>>We have added a column with the temporal expression information for each gene and table footnotes with a description of SCMp and an indication of which genes are nhr-23 targets (only wrt-2).

24. Figure 8C: Is there a label missing from X axis?
>>>Yes, one of the strain names was indeed missing. Based on the comment about the strain names not being informative we have removed the graphs that were the original Fig 8B and C and added all these data to Fig 8A.

25. Figure 8D: The control and expt images appear to be completely different body regions and focal planes. "L4440(RNAi)" = "Control RNAi" elsewhere, be consistent.
>>>We have corrected this issue and provided matched images. We have also ensured that we used control RNAi throughout.

26. line 412: "a set of pre-cuticle, early collagen, protease inhibitor genes" missing word "and"?
>>>"And" was missing and has been added.

27. Line 448: " When we depleted NHR-23 early in L1, animals appeared wild type". But aren't

© 2023. Published by The Company of Biologists under the terms of the Creative Commons Attribution License (https://creativecommons.org/licenses/by/4.0/).

they delayed in progression and still in L1?? Rephrase this, it contributes to the overall confusion surrounding the depletion experiments.
>>>We have rephrased this point to clarify it.

28. Table S3: Why are there 3 columns of P values, what are they? The last column doesn't look like P values. The table key is not clearly connected to what is shown. Overall this is very confusing.
>>>We apologize for the confusion. We have simplified the table with a clearer definition of Enrichment, and just provided the q value which is a p value adjusted for false discovery rate.

29. References: Meeuse 2022 reference appears incomplete (biorxiv)
>>>We have corrected this reference.

Reviewer 2 Advance Summary and Potential Significance to Field:
This manuscript by Johnson et al. describes significant advancements in understanding the function of oscillating expression of the nuclear hormone receptor NHR-23 in C. elegans larval development. NHR-23 was previously shown to be essential for embryonic development and larval molting, but the temporal requirement for NHR-23 was not explored. Here the authors provide compelling evidence that pulses of NHR-23 are necessary for molting and progression to the next larval stage (from my previous review).

The revised manuscript by Johnson et al. is much improved. The authors address my major concern of the original submission regarding the effects of continuous auxin-mediated depletion of NHR-23 during the L1-stage using western blots to demonstrate that NHR-23 levels remain low throughout the L1-stage in auxin treated animals (Figure 2B), and they add some appropriate speculation regarding why larvae depleted of NHR-23 in early L1 stage exhibit less severe molting defects than those treated in later in the L1 stage. They also carefully addressed each of my more minor concerns. This revised version is more clearly written and much easier to read.

Reviewer 2 Comments for the Author:
Thanks for the careful revisions, and your point-by-point response to the earlier reviews.
>>>We thank the reviewer!

Reviewer 3 Advance Summary and Potential Significance to Field:
How extracellular matrices are assembled remains poorly understood. C. elegans serves as an excellent model to explore the mechanism by which apical extracellular matrices (aECMs) are assembled, because the nematode repeatedly reforms aECMs during every molt and their integrity can be easily visualized by fluorescently-tagged proteins. Taking advantage of these features, this paper shows that the nuclear hormone receptor NHR-23/NR1F1 promotes appropriate assembly of components of aECMs by coordinating expression of genes involved in molting, lipid transport/metabolism and aECM components. This study suggests that strictly timed transcription of aECM components plays a key role in robust assembly of aECMs.

Reviewer 3 Comments for the Author:
The authors have appropriately provided point-by-point responses to comments previously raised by reviewers. I have only minor comments below.

Minor Comments:
1) Line 210: This paragraph starts with “To distinguish between larval arrest and developmental delay,..” and eventually ends by stating “these data suggest that NHR-23 depletion causes developmental delay and 233 animals die due to a failed molt.”

Does the last sentence claim that depletion of NHR-23 does NOT cause larval arrest? "wild type" in Fig 3EF is confusing; The size of the “wild type” animal in Fig 3F suggests that the “wild type” animals in Fig 3E are all L1 arrested animals, which are easily recognized by the L1 alae, instead of normally growing larvae/adults. If one would not see any progression of the event of interest during the defined time window, though the penetrance is low, I believe one should mention that the event is “arrested (at least during XX hours)” instead of “delayed”.
>>>We have clarified this section and used the word “arrested” to describe the phenotype at several points.

© 2023. Published by The Company of Biologists under the terms of the Creative Commons Attribution License (https://creativecommons.org/licenses/by/4.0/).

2) Related to the above, Fig S4 and Fig 3EF can be integrated. Auxin exposure at hour 0 as described for Fig 3EF may allow the authors to see the earliest arrest point of M lineage in the "wild type" fraction, instead of variable arrest shown in Fig S4C. That arrest point would be the rate-limiting, developmental step regulated by nhr-23, and may overlap with the time window during which the reporter is detectable (Fig1 B).
>>>In line with the reviewer's suggestion, in response to a comment from Reviewer 1 we have combined Figures 2 and 3, removed the previous Figure S3, and condensed Figure S4 by removing the *daf-16* data. We hope that the section describing the effect of NHR-23 depletion on developmental progression and morphology is shorter and clearer.

3) Line 212: "We performed depletion experiments shifting early L3 animals onto auxin and monitored development by scoring vulva morphology 24 hours later."
Line 1090: "Animals of the indicated genotype were synchronized and shifted onto auxin at 25 hours post- release and imaged 23 hours later (48 hours post-release)."

The main text states the phenotype was scored 24 hours after auxin administration. However, the legend says 23 hours later. Which is true?
>>>We apologize for the confusion. Scoring 23 hours after auxin treatment is correct and we have adjusted the main text.

4) Line 261: Descriptions of Fig 4A, B appear after those of Fig 4D, E. The order of the figures should be changed to avoid confusion.
>>>We have reworked this Figure (now Fig 3) so that the order of elements matches the order they are discussed.

5) Line 277 and Fig 4C: What is the "NHR-23 peaks"? In Fig 4C, the NHR-23 peaks for the x axis seems to mean peaks in expression level. But I do not understand what NHR-23 peaks means for y axis.
>>>We have re-worked this figure. With the correction to the phasing after discussion with the Grosshans lab (See response to Reviewer 1, main point #3) we no longer refer to the "NHR-23 expression peak" on the X-axis. The y-axis now refers to NHR-23 ChIP-seq peak and the X gives larval time. In the legend we clarify where the nhr-23 mRNA expression phase is (peak in expression).

6) Fig 4E was not labelled.
>>>We have corrected this omission (now part of Fig 3).

7) Line 281 and Figure 5: At what developmental time point were these animals "released" onto the auxin plate?
>>>We have clarified that these were synchronized L1 larvae that were released.

8) Fig 6 legend: Explain the numbers on the upper left corner of photos.
>>>In response to a similar critique from Reviewer 1, we have removed those numbers from the images in Figs 5 and 6 and added a clearer explanation in the figure legend.

9) Line 1098: Were the pics of animals taken 48 or 72 hours after the auxin exposure?
>>>These images were taken 72 hours after auxin exposure. We have moved the images to supplemental, removed the 48 hour timepoint, and focused on the 72 hour timepoint.

Second decision letter

MS ID#: DEVELOP/2022/201085

MS TITLE: NHR-23 activity is necessary for C. elegans developmental progression and apical extracellular matrix structure and function

© 2023. Published by The Company of Biologists under the terms of the Creative Commons Attribution License (https://creativecommons.org/licenses/by/4.0/).

AUTHORS: Londen C. Johnson, An A. Vo, John C. Clancy, Krista M Myles, Murugesan Pooranachithra, Joseph Aguilera, Max T. Levenson, Chloe Wohlenberg, Andreas Rechtsteiner, James Matthew Ragle, Andrew D Chisholm, and Jordan D. Ward

I have now received all the referees reports on the above manuscript, and have reached a decision. The referees' comments are appended below, or you can access them online: please go to BenchPress and click on the 'Manuscripts with Decisions' queue in the Author Area.

The overall evaluation is positive and we would like to publish a revised manuscript in Development, once the referees' comments are satisfactorily addressed. I do not expect to send the manuscript back to the reviewers, but please do attend to all of the reviewers' comments in your revised manuscript and detail them in your point-by-point response.

Reviewer 2

Advance summary and potential significance to field

Previous summary of advances:
This manuscript by Johnson et al. describes significant advancements in understanding the function of oscillating expression of the nuclear hormone receptor NHR-23 in C. elegans larval development. NHR-23 was previously shown to be essential for embryonic development and larval molting, but the temporal requirement for NHR-23 was not explored. Here the authors provide compelling evidence that pulses of NHR-23 are necessary for molting and progression to the next larval stage. For these experiments, they use both constitutive and tissue-specific auxin-induced degradation of the protein product of a novel nhr-23 allele. They further demonstrate that NHR-23 functions independently of the well characterized DAF-16/insulin signaling pathway during larval development. Finally, they mine existing datasets of gene expression and NHR-23 ChIP assays to identify a large set of putative NHR-23 targets that also show oscillating expression. The work is generally very well done, and it will impact our understanding of the genes involved in periodic tissue remodeling.

Comments for the author

I feel the authors gave careful attention to and addressed the constructive comments from earlier reviews. In particular, the revised manuscript text and figures clarify the authors’ conclusions. While I felt the previous revision was good, I feel that the authors’ responses to other reviewers points have been thorough and very clear, and they have improved the manuscript.

Reviewer 3

Advance summary and potential significance to field

How extracellular matrices around animal cells are assembled remains poorly understood. Molting of C. elegans serves as an excellent model to explore the mechanism by which apical extracellular matrices (aECMs) are remodeled. This paper shows that the nuclear hormone receptor NHR-23/NR1F1 is essential for coordinating expression of genes involved in molting, lipid transport/metabolism and aECM components and promoting appropriate assembly of components of aECMs. This study suggests that strictly timed expression of aECM components at the appropriate level conferred by NHR-23 plays a key role in robust assembly of aECMs during molting.

The revised manuscript is much improved. Though I was still confused by some parts (as suggested below), I could follow the story of the paper much more easily, compared to the previous one. The authors appropriately responded to each of my previous comments.

Comments for the author

Summary of the advance made in this paper and its potential significance to the field.
How extracellular matrices around animal cells are assembled remains poorly understood. Molting of C. elegans serves as an excellent model to explore the mechanism by which apical extracellular matrices (aECMs) are remodeled. This paper shows that the nuclear hormone receptor NHR-

© 2023. Published by The Company of Biologists under the terms of the Creative Commons Attribution License (https://creativecommons.org/licenses/by/4.0/).

23/NR1F1 is essential for coordinating expression of genes involved in molting, lipid transport/metabolism and aECM components and promoting appropriate assembly of components of aECMs. This study suggests that strictly timed expression of aECM components at the appropriate level conferred by NHR-23 plays a key role in robust assembly of aECMs during molting.

The revised manuscript is much improved. Though I was still confused by some parts (as suggested below), I could follow the story of the paper much more easily, compared to the previous one. The authors appropriately responded to each of my previous comments.

Suggestions to authors. Please structure your report as follows:

The parts of the text where I got little confused are described below. It would be better to reword/revise them unless I misread these.

1) The sentences start with line 250 and line 253 somewhat contradict with each other. The former counts "average NHR-23 peaks flanking and within their gene body." On the other hand, the latter counts "average number of NHR-23 peaks/promoter." Both sentences refer to Fig 3E, but the regions ("flanking and within their gene body" vs "/promoter") covered to calculate the average # of peaks seem different.

2) Line 465: In this paragraph, the authors raise three possibilities (line 473, 478 and 484). However, I could not understand what these possibilities are trying to explain. Do they discuss about line 466 "What is driving NHR-23-depleted animals to eventually attempt to molt?" or line 472-473" The pre-cuticle component, NOAH-1, and early collagen, ROL-6 are both expressed and secreted but display localization defects." The second possibility does not seem to explain either.
"A third possibility is that another timer eventually promotes molting. The molting timer and heterochronic cycles can be uncoupled, causing animals to attempt molting before completion of cell division and differentiation, leading to death (Ruaud and Bessereau, 2006)." General readers could understand better what this sentence means if the authors could explain al little more about what these "timer" s are.

3) Line 493:" (Figure 6)" should be "(Fig. 5 and 6)."

4) Strains used in this study: Some letters that describe genotypes are bold.

5) Materials and Methods: line 542. "IDT Cas9" sounds too casual, since they have sold several kinds of Cas9.

Second revision

Author response to reviewers' comments

>>>We thank both the reviewers for their supportive comments. We have addressed all of reviewer 3's points and hope that the manuscript is now suitable for publication in *Development*.

Reviewer 2 Advance Summary and Potential Significance to Field:
Previous summary of advances:
This manuscript by Johnson et al. describes significant advancements in understanding the function of oscillating expression of the nuclear hormone receptor NHR-23 in C. elegans larval development. NHR-23 was previously shown to be essential for embryonic development and larval molting, but the temporal requirement for NHR-23 was not explored. Here the authors provide compelling evidence that pulses of NHR-23 are necessary for molting and progression to the next larval stage. For these experiments, they use both constitutive and tissue-specific auxin-induced degradation of the protein product of a novel nhr-23 allele. They further demonstrate that NHR-23 functions independently of the well characterized DAF-16/insulin signaling pathway during larval development. Finally, they mine existing datasets of gene expression and NHR-23 ChIP assays to identify a large set of putative NHR-23 targets that also show oscillating expression. The

© 2023. Published by The Company of Biologists under the terms of the Creative Commons Attribution License (https://creativecommons.org/licenses/by/4.0/).

work is generally very well done, and it will impact our understanding of the genes involved in periodic tissue remodeling.

Reviewer 2 Comments for the Author:
I feel the authors gave careful attention to and addressed the constructive comments from earlier reviews. In particular, the revised manuscript text and figures clarify the authors' conclusions. While I felt the previous revision was good, I feel that the authors' responses to other reviewers points have been thorough and very clear, and they have improved the manuscript.

Reviewer 3 Comments for the Author:
Summary of the advance made in this paper and its potential significance to the field. How extracellular matrices around animal cells are assembled remains poorly understood. Molting of C. elegans serves as an excellent model to explore the mechanism by which apical extracellular matrices (aECMs) are remodeled. This paper shows that the nuclear hormone receptor NHR-23/NR1F1 is essential for coordinating expression of genes involved in molting, lipid transport/metabolism and aECM components and promoting appropriate assembly of components of aECMs. This study suggests that strictly timed expression of aECM components at the appropriate level conferred by NHR-23 plays a key role in robust assembly of aECMs during molting. The revised manuscript is much improved. Though I was still confused by some parts (as suggested below), I could follow the story of the paper much more easily, compared to the previous one. The authors appropriately responded to each of my previous comments.

Suggestions to authors. Please structure your report as follows:
The parts of the text where I got little confused are described below. It would be better to reword/revise them unless I misread these.

1) The sentences start with line 250 and line 253 somewhat contradict with each other. The former counts "average NHR-23 peaks flanking and within their gene body." On the other hand, the latter counts "average number of NHR-23 peaks/promoter." Both sentences refer to Fig 3E, but the regions ("flanking and within their gene body" vs "/promoter") covered to calculate the average # of peaks seem different.
>>>We thank the reviewer for catching this apparent contradiction. The former (NHR-23 peaks flanking and within gene bodies) is correct and we have corrected the 2nd sentence.

2) Line 465: In this paragraph, the authors raise three possibilities (line 473, 478 and 484). However, I could not understand what these possibilities are trying to explain. Do they discuss about line 466 "What is driving NHR-23-depleted animals to eventually attempt to molt?" or line 472-473" The pre-cuticle component, NOAH-1, and early collagen, ROL-6 are both expressed and secreted but display localization defects." The second possibility does not seem to explain either. "A third possibility is that another timer eventually promotes molting. The molting timer and heterochronic cycles can be uncoupled, causing animals to attempt molting before completion of cell division and differentiation, leading to death (Ruaud and Bessereau, 2006)." General readers could understand better what this sentence means if the authors could explain al little more about what these "timer" s are.
>>>We appreciate the reviewer flagging this confusing passage. We have removed the sentence "The pre-cuticle component, NOAH-1, and early collagen, ROL-6 are both expressed and secreted but display localization defects." As it did not add to the discussion. We added some text to build on the first possibility of low NHR-23 levels being sufficient to initiate but not execute molting. We removed the discussion about the 2nd possibility (a regulatory cascade) as the other two models were stronger and had clearer rationale. The 2nd model didn't clearly address why NHR-23-depleted animals attempt to molt eventually. We expanded on the third possibility (other timers), giving more background to help general readers.

3) Line 493:" (Figure 6)" should be "(Fig. 5 and 6)."
>>>We have made this correction.

4) Strains used in this study: Some letters that describe genotypes are bold.
>>>We have removed the bold in the genotypes.

5) Materials and Methods: line 542. "IDT Cas9" sounds too casual, since they have sold several

© 2023. Published by The Company of Biologists under the terms of the Creative Commons Attribution License (https://creativecommons.org/licenses/by/4.0/).

kinds of Cas9.
>>>We have added the specific Cas9 used from IDT (Alt-R™ S.p. Cas9 Nuclease V3).

Third decision letter

MS ID#: DEVELOP/2022/201085

MS TITLE: NHR-23 activity is necessary for C. elegans developmental progression and apical extracellular matrix structure and function

AUTHORS: Londen C. Johnson, An A. Vo, John C. Clancy, Krista M Myles, Murugesan Pooranachithra, Joseph Aguilera, Max T. Levenson, Chloe Wohlenberg, Andreas Rechtsteiner, James Matthew Ragle, Andrew D Chisholm, and Jordan D. Ward
ARTICLE TYPE: Research Article

I am happy to tell you that your manuscript has been accepted for publication in Development, pending our standard ethics checks.

© 2023. Published by The Company of Biologists under the terms of the Creative Commons Attribution License (https://creativecommons.org/licenses/by/4.0/).
